# Potential role of the regulatory *miR1119*-*MYC2* module in wheat (*Triticum aestivum* L.) drought tolerance

**DOI:** 10.3389/fpls.2023.1161245

**Published:** 2023-05-30

**Authors:** Roohollah Shamloo-Dashtpagerdi, Amir Ghaffar Shahriari, Aminallah Tahmasebi, Ramesh R. Vetukuri

**Affiliations:** ^1^ Department of Agriculture and Natural Resources, Higher Education Center of Eghlid, Eghlid, Iran; ^2^ Department of Agriculture, Minab Higher Education Center, University of Hormozgan, Bandar Abbas, Iran; ^3^ Department of Plant Breeding, Swedish University of Agricultural Sciences, Lomma, Sweden

**Keywords:** noncoding RNAs, miRNA, transcription factor, systems biology, expressed sequence tag, abiotic stress, drought

## Abstract

MicroRNA (miRNA)-target gene modules are essential components of plants’ abiotic stress signalling pathways Little is known about the drought-responsive miRNA-target modules in wheat, but systems biology approaches have enabled the prediction of these regulatory modules and systematic study of their roles in responses to abiotic stresses. Using such an approach, we sought miRNA-target module(s) that may be differentially expressed under drought and non-stressed conditions by mining Expressed Sequence Tag (EST) libraries of wheat roots and identified a strong candidate (*miR1119-MYC2*). We then assessed molecular and physiochemical differences between two wheat genotypes with contrasting drought tolerance in a controlled drought experiment and assessed possible relationships between their tolerance and evaluated traits. We found that the *miR1119-MYC2* module significantly responds to drought stress in wheat roots. It is differentially expressed between the contrasting wheat genotypes and under drought versus non-stressed conditions. We also found significant associations between the module’s expression profiles and ABA hormone content, water relations, photosynthetic activities, H_2_O_2_ levels, plasma membrane damage, and antioxidant enzyme activities in wheat. Collectively, our results suggest that a regulatory module consisting of *miR1119* and *MYC2* may play an important role in wheat’s drought tolerance.

## 1 Introduction

Abiotic stresses like drought compromise plant survival and significantly reduce their growth and yield (Pereira, 2016). Drought stress can result in various physiological and biochemical reactions in plants (Ilyas et al., 2021). It leads to a substantial change in plant water relations, which drives osmotic and oxidative stresses that cause the partial closure of stomata and reductions in photosynthesis, generation of reactive oxygen species (ROS), activation of plant antioxidant system (AOS), and altered cell homeostasis (Rane et al., 2022). To adjust these reactions and to minimize the adverse effects of drought stress, plants recruit multiple and sometimes overlapping signalling pathways. These pathways provide highly variable genotype-dependent tolerance of drought stress, and associated variation in survival and productivity (Shamloo-Dashtpagerdi et al., 2019; Shamloo-Dashtpagerdi et al., 2022b; Zhang et al., 2022b). The regulatory components located in the heart of signalling pathways are the leading players in controlling responses to drought (Shinozaki and Yamaguchi-Shinozaki, 2007; Zhang et al., 2022b). Thus, identifying and characterizing the key regulatory components is crucial for efforts to improve crops’ drought tolerance.

Plant miRNAs (typically 20-24 nucleotides long) play key roles in post-transcriptional gene regulation (Nogoy et al., 2018). Genes encoding them are transcribed by RNA polymerase II. Processing of the transcripts by 5′ capping, splicing, and polyadenylation at the 3′end yields pri-miRNA. Further processing results in precursor RNA (pre-miRNA) with a stem-loop structure, which is cleaved by DCL1, giving rise to mature miRNA-miRNA* duplexes with a two nucleotide overhang at the 3′ end (Tiwari et al., 2021a; Tiwari et al., 2021b). Mature miRNA binds to target messenger RNA (mRNA) and negatively regulate target genes (Hou et al., 2019). miRNAs and their target genes form regulatory modules that are key components of abiotic stress signalling pathways that fine-tune various adaptive stress responses in plants (Millar, 2020; Pagano et al., 2021; Zhang et al., 2022a). Clearly, identifying miRNA-target modules involved in responses to abiotic stresses is highly desirable because it can provide important new insights into plants’ stress tolerance mechanisms. Thus, both experimental and computational methods have been used in efforts to identify and characterize modules involved in the responses of diverse plant species to various environmental stresses. For instance, Liu et al. (2019) showed that several miRNA-target modules (including *miR164*-*MYB*, *miR164*-*NAC*, *miR159*-*MYB*, *miR156*-*SPL* and *miR160*- *ARF*) are differentially regulated in maize inbred lines with contrasting drought sensitivities. Water deficits also reportedly reduce expression of the miRNA-target modules *miR159*-*MYB*, *miR396*-*GRF*, *miR535*-*SPL*, *miR166b*-*HD-ZIP* III and *miR167*-*ARF* in cardamom (*Elettaria cardamomum* Maton) (Anjali et al., 2019). In addition, the *miR168a*-*OMT1* module contributes to the salinity tolerance of *Brassica rapa* L., mainly through the regulation of melatonin biosynthesis (Shamloo-Dashtpagerdi et al., 2022). There is also some evidence that the *miR1118*-*PIP1;5* module participates in wheat plants’ salinity tolerance through adjustment of their water status and ion homeostasis, and mitigation of membrane damage (Shamloo-Dashtpagerdi et al., 2022c).

Bread wheat is one of the world’s most important cereal crops, that drought stress significantly reduces its productivity, thus threatening global food security (Juliana et al., 2019; Sallam et al., 2019). This is because drought reduces plants’ Relative Water Content (RWC) and induces stomatal closure, thereby reducing stomatal conductance and photosynthetic rates, often accompanied by chloroplast damage, chlorophyll photo-oxidation, and metabolic distortions that further limit photosynthesis rates and reduce grain yields (Sallam et al., 2019). Moreover, peroxidation of cell membranes through ROS production, changes in morphological characteristics, and nutrient uptake reduction have been frequently reported in wheat exposed to drought stress (Ahmad et al., 2018; Sallam et al., 2019). Therefore, identifying the genes controlling such physiological and biochemical traits, and elucidating the regulation of wheat genes under drought stress, is crucial to accelerate the genetic improvement of wheat’s drought tolerance. Little is known about the drought-modulated miRNAs, their target genes, and related physiochemical responses in wheat. However, advances in systems biology approaches involving combinations of computational biology, statistical and experimental methods, have enabled detailed study of miRNA-target modules and related physiochemical changes in responses to abiotic stresses (Baltoumas et al., 2021; Pagano et al., 2021). Therefore, we designed the study presented here to identify possible miRNA-target module(s) involved in wheat responses to drought stress. For this purpose, we first mined EST libraries of wheat exposed to drought stress using various computational and bioinformatics methods to identify possible drought-responsive miRNA-target modules and identified a strong candidate (*miR1119-MYC2*). Next, we characterized molecular and physiochemical differences between two genotypes with different degrees of stress tolerance in a controlled drought experiment and explored the relationships between drought tolerance and evaluated characteristics. The results indicate that the *miR1119*-*MYC2* module might contribute to wheat drought tolerance by regulating multiple physiochemical responses.

## 2 Materials and methods

### 2.1 Computational analysis

#### 2.1.1 Data sources

EST data were retrieved from resources available *via* the website of the US wheat genome project (http://wheat.pw.usda.gov/NSF/data.html) (Chao et al., 2006). These included 5′EST libraries obtained from roots of hexaploid wheat (*Triticum aestivum* L. cv Chinese Spring) under both unstressed conditions (TA058E1X, containing 1025 sequences, named NS), and the drought conditions (TA055E1X, containing 1310 sequences, named DS) (Zhang et al., 2004). Briefly, the experimental procedure was as follows:

Plants of genotype Chinese Spring were subjected to drought conditions so that RWC values in their leaves reached between 60 and 80%. Then, total RNA was extracted from roots of control and treated plants at the full tillering stage. Extracted total RNA samples were mixed and used for cDNA synthesis and sequencing (https://harvest.ucr.edu/).

#### 2.1.2 EST processing

EST sequences of the NS and DS libraries were subjected to stepwise processing using the EGassembler online bioinformatics service (http://egassembler.hgc.jp) (Masoudi-Nejad et al., 2006). Vector contaminants were removed, and sequences similar to plastids and repetitive sequences were also excluded. If the trimmed sequences were less than 100 bp or had more than 4% ambiguous bases they were excluded from further analysis (Masoudi-Nejad et al., 2006; Bouck and Vision, 2007). The remaining high-quality ESTs were used for downstream analysis.

#### 2.1.3 *In silico* gene expression analysis

All high-quality ESTs of the NS and DS libraries identified in the previous steps were clustered and assembled into unigenes (contigs and singletons), using EGassembler with an overlap percent identity cutoff >80%. A Python script was developed to quantify expression levels for each contig (gene) based on the number of ESTs of each library contributing to it. To identify differentially expressed genes (DEGs) under the drought and control treatments, Audic and Claverie (AC) statistical test (Audic and Claverie, 1997) implemented in ACDtool (http://www.igs.cnrs-mrs.fr/acdtool/) (Claverie and Ta, 2019) was used. A gene was considered to be differentially expressed if the *p*-value obtained with Benjamini–Hochberg False Discovery Rate (FDR) (Benjamini and Hochberg, 1995) was ≤ 0.05.

#### 2.1.4 Functional annotation of DEGs

BLASTN search against National Center for Biotechnology Information (NCBI) Non-redundant nucleotide sequences (nt) database and IWGCS RefSeq v2.1 (https://www.wheatgenome.org/) were done for identified DEGs using CLC Genomics Workbench 9.5 software and an E-value ≤ 10^-5^. In addition, BLASTX search vs NCBI Non-redundant protein sequences (nr), UniProt, and The Arabidopsis Information Resource (TAIR) protein databases were performed using the same software and the same E-value. The DEGs were then subjected to gene set enrichment analysis using GeneCodis4 (https://genecodis.genyo.es/) (Garcia-Moreno et al., 2022). In GeneCodis4, the hypergeometric statistical test, the minimum number of genes in each category (*n* = 3) and *p*-value ≤ 0.05 were selected. In addition, all informative ESTs from the two libraries (2335 sequences that yielded a significant hit in the BLASTX searches) were used as the background set. Statistically significant functional categories were illustrated by bubble plot using the SRplot online tool (http://www.bioinformatics.com.cn/srplot).

#### 2.1.5 Computational identification of differentially expressed miRNA-target modules

Potential genes encoding miRNAs in the identified DEGs were predicted using the miRkwood online tool (https://bioinfo.cristal.univlille.fr/mirkwood/mirkwood.php) (Guigon et al., 2019). Therefore, putative coding sequences, tRNAs, and rRNAs among the DEGs were excluded using BLAST searches implemented in miRkwood (E-value=1E-5). The Minimal Folding Free Energy Index (MFEI) of pre-miRNAs was adjusted to less than or equal to -0.6 (Zhang et al., 2006).

A set of the revised criteria of plant miRNAs provided by Axtell and Meyers (2018) was also applied to strengthen our computational predictions, by:

- Excluding miRNA/miRNA* duplexes containing secondary stems or large loops
- Allowing up to five mismatched positions in miRNA/miRNA* duplexes, with at most three in asymmetric bulges
- Including only mature miRNAs that were 20 to 24 nucleotides long
The psRNATarget server (https://www.zhaolab.org/psRNATarget/) (Dai et al., 2018) was used to find the putative target gene(s) of identified miRNAs among the DEGs, with the following default parameters:
- Maximum expectation for complementarity, 5
- Penalty for G:U pair, 0.5
- Penalty for other mismatches, 1
- Extra weight in seed region, 1.5
- Seed region, 2-13 Nt
- Number of mismatches allowed in seed region, 2
- Length of scoring region for complementary analysis (HSP size), 19
- Bulge (gap), allowed

Therefore, the predicted miRNA(s) identified in the previous step were searched against all sequences of the identified DEGs. Genes with a lower expectation value and target accessibility-maximum energy to unpair the target site (UPE) were selected as target genes (Dai and Zhao, 2011; Dai et al., 2018). Accordingly, based on the applied parameters, the most reliable miRNA-target module was selected for downstream analysis.

#### 2.1.6 Pathway analysis

All identified DEGs with significant BLAST scores along with the predicted miRNA were categorized into significant pathways using the Pathway Studio software (https://www.pathwaystudio.com/) (Nikitin et al., 2003) with the following parameters:

- Neighbour groups to assess: small molecules, functional class, complex, protein
- Connection type: expression, regulation, molecular transport, promoter binding, molecular synthesis, and chemical reaction
- Enrichment *p*-value cut-off: 0.05

Then significantly associated pathways were networked using the same software with a hierarchical layout.

### 2.2 Drought stress experiment

#### 2.2.1 Plant material and drought treatment

Seeds of the drought stress-tolerant wheat genotype Sirvan and susceptible genotype Moghan3 (Riasat et al., 2018; Mehrabad Pour-Benab et al., 2019; Shamloo-Dashtpagerdi et al., 2022a) were surface-sterilized and stratified in Petri dishes on soaked filter paper at 4 ^°^C for three days to synchronize germination. The resulting seedlings were transferred to pots, each containing 2 kg of sandy-clay soil. The pots were then kept in a controlled greenhouse with 24 ^°^C/18 ^°^C day/night temperatures, 10–12 h natural photoperiods and irrigation based on the soil field capacity (FC), from late December to early February 2021. The pots were divided into non-stressed (control) and stressed groups at the 5-leaf growth stage. The former were fully irrigated with tap water and the latter were subjected to drought stress by withholding irrigation and keeping them at 60% FC until the end of the experiment. Roots were sampled at three time points (3, 24, and 48 h after the drought stress treatment was initiated) from five replicates (pots) with a single plant per treatment and time point.

#### 2.2.2 qPCR analyses of the identified miRNA-target module

Total RNA was extracted from root samples of both wheat genotypes under drought conditions using a Column RNA isolation kit (DENAzist Asia, Iran) following the manufacturer’s recommended protocols. The quality and quantity of RNA were checked by separating the major ribosomal RNA bands in 2% agarose gels and spectrophotometry at two wavelength ratios of A_260/230 _and A_260/280 _nm (NanoDrop, Technologies Inc). Possible genomic DNA contamination was eliminated with RNase-free DNase I (Jena Bioscience, Germany) treatment following the manufacturer’s recommendations.

Specific stem-loop RT primers for predicted miRNA expression profiling were designed according to Varkonyi-Gasic et al. (2007) (**Table S1**) and applied to reverse transcribe the total RNA to cDNA using an Easy cDNA Synthesis Kit (Pars, Iran), following the manufacturer’s instructions. They were then used in quantitative polymerase chain reaction (qPCR) assays with a Bioer thermal cycler system (Bioer Technology Co., Ltd., China), 2X SYBR Green Real-Time PCR kit (Pars, Iran) and five biological and two technical replicates per sample. The *T. aestivum* rRNA26 homolog was used as the internal standard of qPCR to normalize the miRNA expression (**Table S1**). Relative gene expression values were calculated using the 2^-ΔΔCt^ method (Livak and Schmittgen, 2001).

To evaluate the expression profile of the predicted target gene, 2 mg samples of DNase-treated RNA were used to synthesize the first strand cDNA using an Easy cDNA Synthesis Kit (Pars, Iran) following the manufacturer’s recommendations. Gene-specific primers for predicted target gene were designed according to the corresponding contig sequence using AlleleID software 7.7. Its expression levels were then assessed by qPCR, again using the Bioer thermal cycler system, a 2X SYBR Green Real-Time PCR kit (Pars, Iran), five biological and two technical replicates per sample, and the 2^-ΔΔCt^ method (Livak and Schmittgen, 2001). However, the *Actin* gene (GenBank: AB181991.1) was used as an internal standard (**Table S1**).

#### 2.2.3 Determination of ABA content

Roots of untreated and drought-treated plants were thoroughly washed with tap water to remove soil, then samples were extracted with 80% ethanol in a 1:10 *w*/*v* ratio at room temperature. The resulting extract was separated by filtration, evaporated to an aqueous residue, and diluted with distilled water. Then the residues were acidified to pH 2.5 with dilute HCl (ratio of organic to aqueous phases being 3:1). The resulting solutions were shaken with three volumes of saturated NaHCO_3_ solution (pH 8-9) to neutralize acidic compounds, then ABA was extracted from the aqueous phase with diethyl ether (Vysotskaya et al., 2004), and quantified by enzyme-linked immunoassay (ELISA) using specific antibodies as described by Arkhipova et al. (2020).

#### 2.2.4 Evaluation of photosynthetic parameters

Photosynthetic parameters were evaluated using the method described by Figueroa-Bustos et al. (2020) with a few modifications. Briefly, fully expanded leaves of each genotype were used to evaluate their photosynthesis rate (*A*_N_; net CO_2_ assimilation), transpiration rate (*E*), and stomatal conductance (*g*_s_), with a Li-6400 portable photosynthesis system (LI-COR Inc., Lincoln, NE, USA). All measurements were carried out between 10:00 am and 1:00 pm, with a leaf temperature of 26 ^°^C and photon flux density of 500 mmol m^-2^ s^-1^. The flow rate through the chamber was 500 mmol s^-1^. The leaves’ chlorophyll contents were determined using a Portable Minolta SPAD 502 Plus chlorophyll meter (Delta T, UK) in terms of SPAD values. In addition, the Intrinsic Water Use Efficiency (WUEi) was calculated as the ratio between *A*_N_ and *g*_s_ (*A*_N_/*g*_s_; μmol CO_2_ mol^-1^ H_2_O) (Franco-Navarro et al., 2016).

#### 2.2.5 Evaluation of antioxidant enzyme activities

Portions of sampled roots were ground and homogenized in liquid nitrogen, then extracted with 50 mM sodium phosphate buffer (pH 7.0) containing 2 mM EDTA, 5 mM β-mercaptoethanol and 4% (*w*/*v*) PVP. The resulting extracts were centrifuged at 30,000 g for 30 min at 4 ^°^C and the supernatants were immediately used to determine activities of antioxidant enzymes as follows. Superoxide dismutase (SOD; EC 1.15.1.1) activity was determined from the inhibition of nitro blue tetrazolium (NBT) reduction under light (Stewart and Bewley, 1980). Peroxidase (POD; EC 1.11.1.7) activity was measured by determining guaiacol oxidation from changes in absorbance at 470 nm after adding H_2_O_2_ in a spectrophotometer (Shah and Nahakpam, 2012). Catalase (CAT; EC 1.11.1.6) activity was measured by monitoring absorbance at 240 nm to follow the decomposition of H_2_O_2_ to H_2_O (Patra et al., 1978).

#### 2.2.6 Physiochemical assays

The RWC of leaves of both wheat genotypes was measured using the protocol described by Sade et al. (2015). The proline content of the root samples was evaluated according to Bates et al. (1973). H_2_O_2_ contents were measured by detecting titanium-peroxide complex formation at 415 nm spectrophotometrically (Yu et al., 2019). Cell membrane damage due to stress was measured by monitoring electrolyte leakage (EL) from roots of the wheat genotypes, following Sairam and Srivastava (2002).

#### 2.2.7 Statistical analysis

The acquired expression and physiochemical data were subjected to analysis of variance (ANOVA) with comparisons of means by Duncan’s multiple range test (*p*-value ≤ 0.01) using STATISTICA v. 12 software (https://www.statistica.com/en/). To investigate the relationships among the measured characteristics, including expression profiles of the identified miRNA-target module, ABA content, photosynthetic properties, antioxidant activities and physiochemical parameters, Principal Component Analysis (PCA) was performed based on Pearson correlations with varimax rotation (Corner, 2009) implemented in XLSTAT software 2019.2.2 (https://www.xlstat.com).

## 3 Results

### 3.1 EST sequences analysis and identification of DEGs

Of the 2335 raw EST sequences of wheat roots in the non-stress (NS) and drought stress (DS) libraries, 238 were trimmed during sequence processing by EGassembler, and no sequence was removed. These high-quality sequences, with an average length of 637 bp, were clustered and assembled into 1462 unigenes (475 contigs and 987 singletons). The minimum, maximum, and average lengths of the contigs were 120, 2243, and 675 bp, respectively (**Table S2**). The number of ESTs in contigs ranged from 2 (279 contigs) to 67 (one contig), indicating different expression levels of genes.

**Figure 1:**
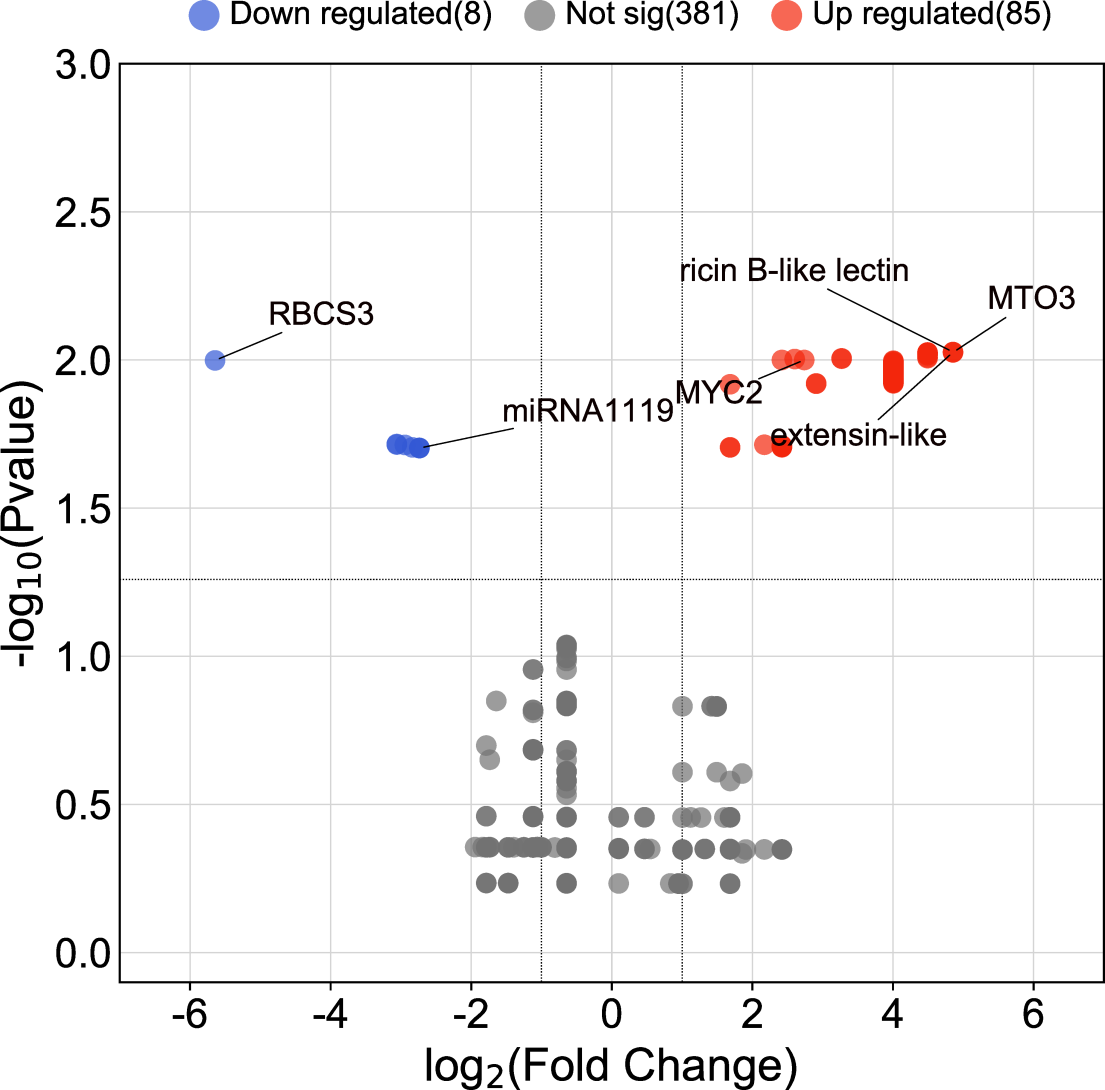
Volcano plot of DEGs between roots of drought-stressed and non-stressed wheat plants. Genes with the highest expression changes, the identified miRNA, and predicted target gene are shown.

The AC test revealed that a total of 93 contigs (genes) were differentially expressed in response to drought (FDR ≤ 0.05). Of those, 85 genes were upregulated, and 8 genes were downregulated under the drought treatment. Contig 455 (encoding MTO3 S-adenosylmethionine synthetase), contig 399 (encoding extensin-like), and contig 392 (encoding ricin B-like lectin) were upregulated most strongly (4.85-fold), and contig 330 encoding ribulose bisphosphate carboxylase small subunit, chloroplastic 3 was most strongly downregulated (-5.64 folds) (**Figure 1** and **Supplementary File 1**).

### 3.2 Functional interpretation of DEGs

Identified DEGs were annotated according to results of BLAST searches against the NCBI nt, NCBI nr, Swiss-Prot, and TAIR databases. These revealed that 98.9, 75.3, 95.7, 89.2 and 84.9% of the DEGs (92, 70, 89, 83, and 79 genes) had significant hits in the NCBI nt, IWGCS, NCBI nr, Swiss-Prot, and TAIR databases, respectively (**Table S3**).

**Figure 2:**
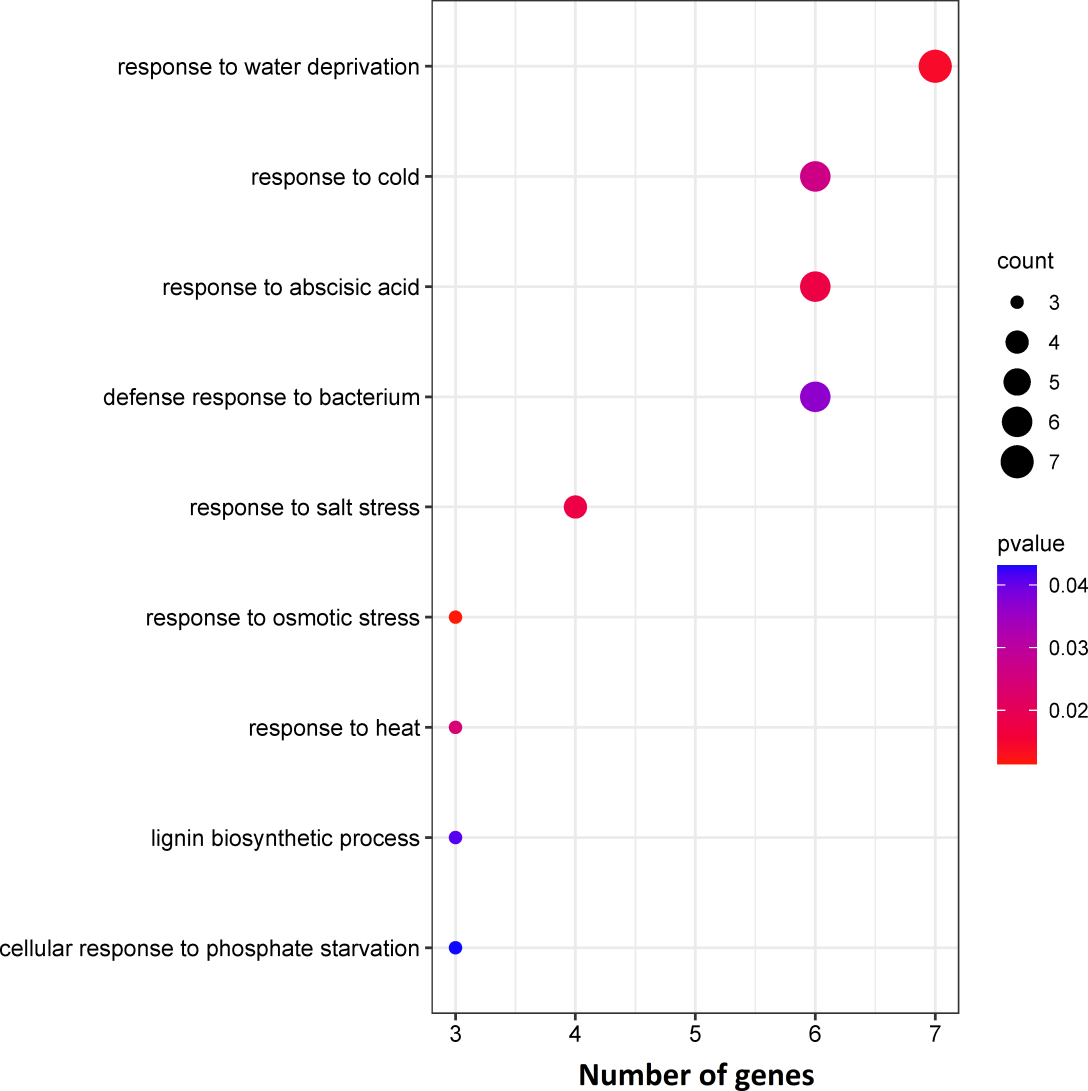
Results of enrichment analysis of identified DEGs based on biological processes using the GeneCodis tool.

The DEGs were assigned to 10 statistically significant biological process categories (*p*-value ≤ 0.05) (**Figure 2**), most frequently: “response to water deprivation”, “response to cold”, “response to abscisic acid”, “defence response to bacterium” and “response to salt stress” (7, 6, 6, 6 and 4 genes, respectively) (**Figure 2**). Three genes were also assigned to each of the remaining categories: “leaf development”, “cellular response to phosphate starvation”, “response to osmotic stress”, “response to heat” and “lignin biosynthetic process” (**Figure 2**). These results indicate that most identified enriched DEGs are involved in abiotic stress responses.

### 3.3 Computational identification of miRNA-target modules among DEGs

**Figure 3:**
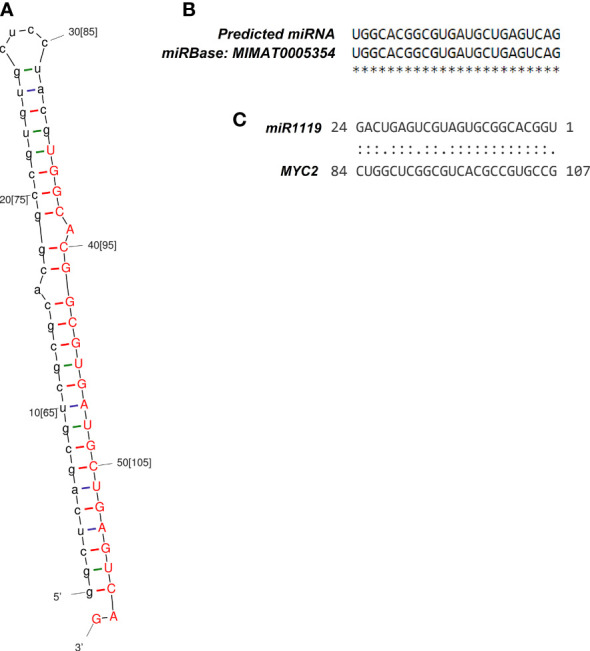
Structural characteristics of the identified miRNA-target module. **(A)** Stem-loop structure of identified wheat *miR1119*, with the 24-nt mature miRNA shown in red. **(B)** Alignment of the identified *miR1119* and *T. aestivum* L. *miR1119* from miRBase. **(C)** Binding site of the *miR1119-MYC2* module.

Only one DEG met the key criteria for consideration as a potential miRNA-encoding gene. Accordingly, miRNA identified on contig 113 with 207 bp in length had an MFEI index of -1.16. The mature miRNA was located at 42 to 65 nt of contig 113, with 24 bp lengths (**Figure 3A**), and significantly matched an entry in the miRbase database (*T. aestivum* L*. miR1119*) with no mismatch (**Figure 3B**).

Using the psRNATarget tool, one target gene among the DEGs was identified for *miR1119* with an expectation value of 1.5 and UPE value of 38.08. Moreover, psRNATarget predicted that *miR1119* regulates expression of its target gene by cleavage. The identified target gene corresponded to contig 247, encoding MYC2 protein. The binding region of *miR1119* to the target gene is shown in **Figure 3C**.

Based on EST sequences analysis, the expression profile of the identified miRNA and its target gene was as expected, with downregulation of the identified miRNA and upregulation of the predicted target gene under drought conditions (**Figure 1**).

### 3.4 Pathway analysis

**Figure 4:**
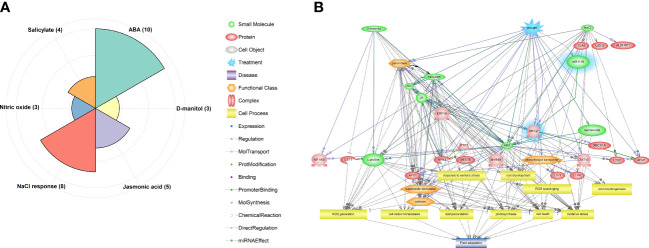
Results of pathway analysis of the identified DEGs, including the predicted *miR1119-MYC2* module. **(A)** Enriched pathways according to the hypergeometric test (*p*-value ≤ 0.05). **(B)** Hierarchical network of components of the enriched pathways, with the *miR1119-MYC2* module highlighted in blue.

As shown in **Figure 4A**, identified DEGs, including *miR1119* and *MYC2*, were enriched in six significant pathways (*p*-value ≤ 0.05), including ABA (10 genes), NaCl response (8 genes), Jasmonic acid (5 genes), Salicylate (4 genes), Nitric oxide (NO) (3 genes) and D-mannitol (3 genes). Hierarchical relationships among genes, small molecules, functional classes, and cell processes incorporated in significant pathways are shown in **Figure 4B**. This visualization revealed that various TFs and protein kinases (PKs) involved in ABA, JA, salicylate, and NO signalling pathways are recruited in drought responses. Moreover, it revealed that processes controlled by these signalling pathways, including photosynthesis, root development and morphology, redox homeostasis, lipid peroxidation, cell death, and activation of antioxidant enzymes, play key roles in wheat’s drought responses. The identified module also seems to be an important upstream component in the predicted network, corroborating the importance of the *miR1119*-*MYC2* module in these responses.

### 3.5 qPCR assay of identified miRNA-target module under drought conditions

**Figure 5:**
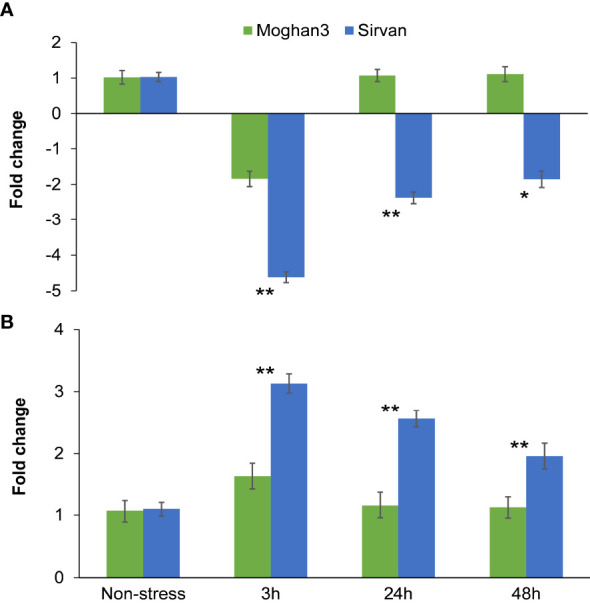
Expression profiles of the *miR1119-MYC2* module in roots of the Sirvan and Moghan3 wheat genotypes under drought conditions: **(A)** *miR1119* and **(B)** *MYC2*. The values are means ± SE (*n* = 5 biological replicates, each consisting of one plant with two technical replicates). Significant differences between the two genotypes according to Duncan’s multiple range test (*p*-value ≤ 0.01) are indicated by asterisks: *p ≤ 0.05, **p ≤ 0.01.

The qPCR results revealed that *miR1119* expression significantly differed between the wheat genotypes and under the two treatments (**Figure 5A**). Its expression was significantly lower (about 2-fold) at the 3 h time point under drought than under the non-stressed conditions in the susceptible genotype Moghan3 (**Figure 5A**). However, there were no significant differences in its expression at the later (24 and 48 h) time points in Moghan3 (**Figure 5A**). In contrast, in roots of the tolerant genotype Sirvan. *miR1119* was dramatically downregulated (about 4.5-fold) at 3 h under the drought treatment and although its expression subsequently increased it remained significantly lower under drought stress than under control conditions

In both genotypes, *MYC2* was conversely expressed relative to *miR1119*, suggesting that miR1119 regulates the target gene through a cleavage mechanism. *MYC2* was slightly, but significantly, upregulated under drought stress at 3 h in Moghan3, but there was no significant between-treatment difference in its expression in Moghan3 at the 24 and 48 h time points (**Figure 5B**). In contrast, *MYC2* expression was significantly higher under drought than under control conditions at all time points in the tolerant genotype, Sirvan (**Figure 5B**). Furthermore, *MYC2* expression was significantly higher in Sirvan than in Moghan3 under the drought treatment at all time points (**Figure 5B**). Maximum upregulation (up to 3-fold) of *MYC2* was also observed at the 3 h time point (**Figure 5B**), and MYC*2* transcript levels were consistently higher in the tolerant genotype than in the susceptible genotype under drought stress.

Taken together, the computational findings and expression patterns strongly indicate that *miR1119* may regulate expression of the *MYC2* gene.

### 3.6 Effects of drought on root ABA content

**Figure 6:**
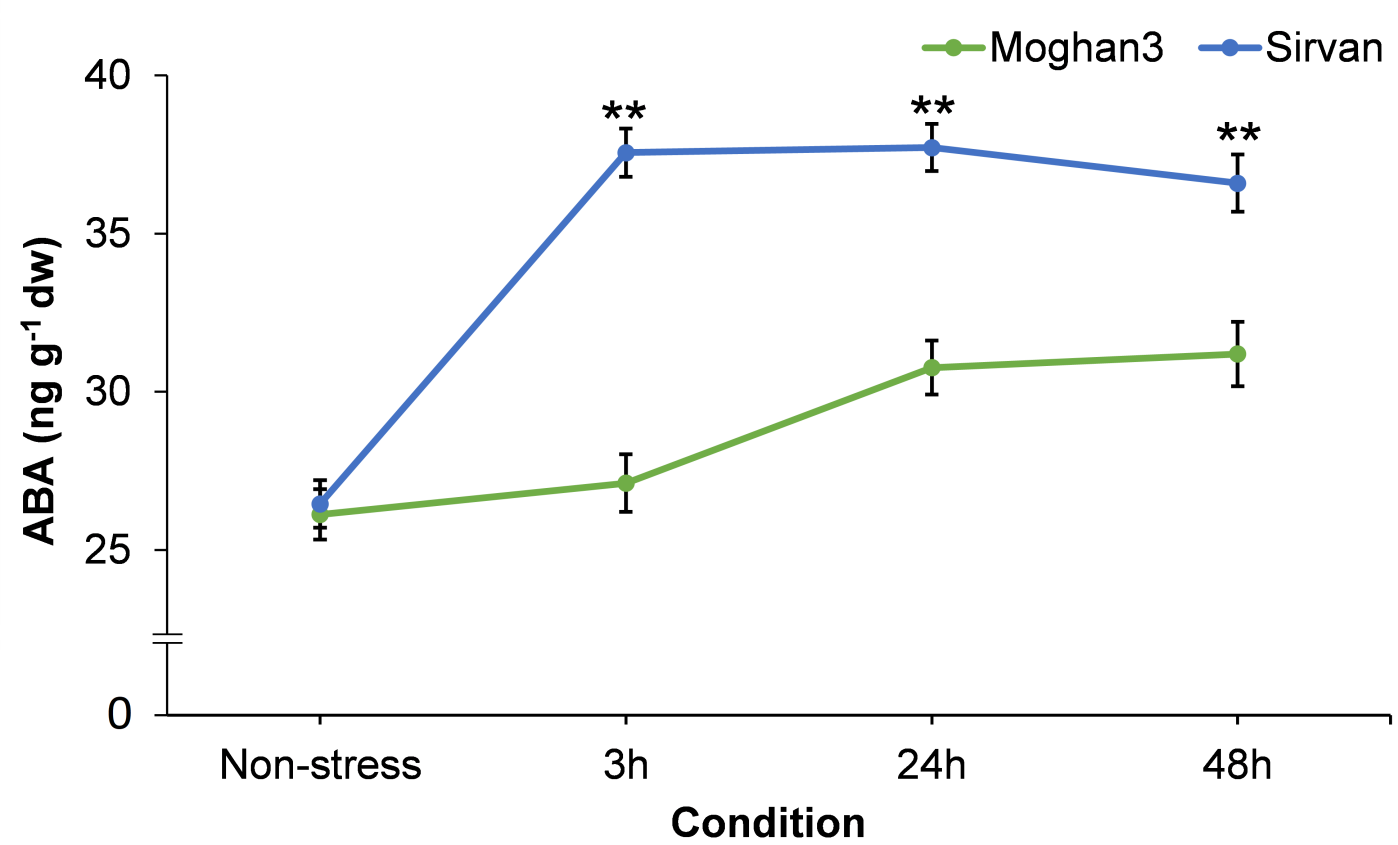
ABA contents in roots of the Sirvan and Moghan3 wheat genotypes measured under drought conditions. The values are means ± SE (*n* = 5 biological replicates). Significant differences between the genotypes according to Duncan’s multiple range test (*p*-value ≤ 0.01) are indicated by asterisks: *p ≤ 0.05, **p ≤ 0.01.

A considerable difference between the contrasting wheat genotypes was observed in root ABA content. In the tolerant genotype Sirvan it was significantly elevated after 3 h of the drought treatment (**Figure 6**) and its level did not significantly change from then until the end of the experiment (**Figure 6**). There was no significant between-treatment in the ABA content of roots of the susceptible wheat genotype Moghan3 at the 3 h time point (**Figure 6**). It was significantly higher under drought stress than in control conditions at the 24 and 48 h time points in Mogan3 roots, but still significantly lower than in Sirvan roots under drought at all time points (**Figure 6**).

### 3.7 Effects of drought on photosynthetic parameters

The drought treatment significantly reduced the photosynthetic rate (*A*_N_) in both wheat genotypes. However, the reduction was considerably stronger in Moghan3 than in Sirvan (**Table 1**). In both genotypes, the *E* and *g*_s_ were also significantly reduced, especially at the 48 h time point, under the drought treatment (**Table 1**). However, the reduction were significantly greater in Sirvan than in Moghan3 (**Table 1**). The SPAD value significantly decreased under drought conditions in both wheat genotypes and the tolerant genotype had substantially higher SPAD values than the susceptible genotype (**Table 1**). In all time points of drought treatment, WUEi in Sirvan was significantly higher than in Moghan 3. The difference was more considerable at the 48 h time point, under the drought treatment (**Figure S1**).

**Table 1:** Photosynthetic parameters (photosynthetic rate as net CO_2_ assimilation (*A*_N_), transpiration rate (*E*), stomatal conductance (*g*_s_) and Chlorophyll (Chl) content (SPAD)) of the Sirvan and Moghan3 wheat genotypes measured under non-stressed and drought conditions.

	*A*_N_ (µmol CO_2_ m^-2^ s^-1^)		*E* (mmol H_2_O m^-2^ s^-1^)		*g*_s_ (mol H_2_O m^-2^ s^-1^)		SPAD	
	Sirvan	Moghan3	Sirvan	Moghan3	Sirvan	Moghan3	Sirvan	Moghan3
Non-stress	16.310^a^	16.134^a^	3.805^a^	3.785^a^	0.097^a^	0.097^a^	33.397^a^	33.435^a^
3 h	15.842^b^	15.050^c^	2.696^c^	3.089^b^	0.09^c^	0.093^b^	32.761^b^	32.554^b^
24 h	13.314^d^	12.321^e^	2.070^e^	2.421^d^	0.081^e^	0.086^d^	30.875^c^	29.130^d^
48 h	10.349^f^	8.641^g^	1.014^g^	1.402^f^	0.056^g^	0.064^f^	27.284^e^	24.467^f^

Different letters indicate significant differences in means based on the Duncan test (p-value ≤ 0.01).

### 3.8 Effects of drought on antioxidant enzyme activities

**Figure 7:**
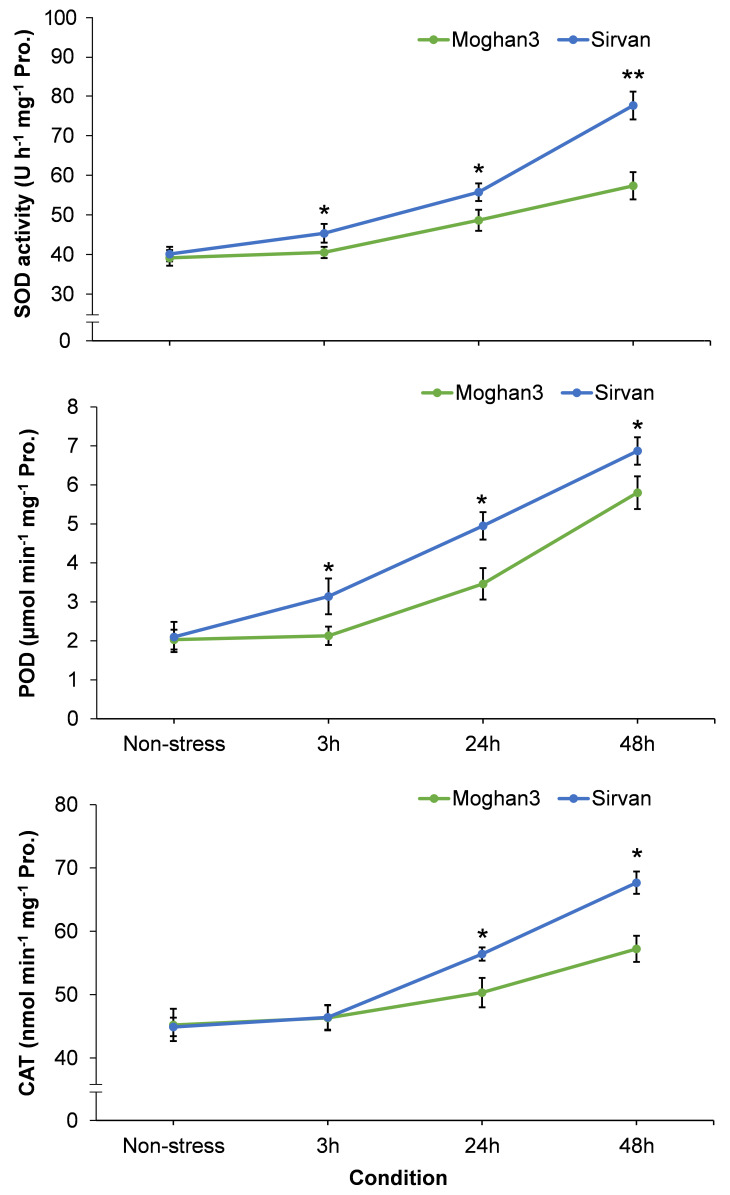
Activities of antioxidant enzymes (SOD, POD, and CAT) in roots of the Sirvan and Moghan3 wheat genotypes under drought conditions. The values are means ± SE (*n* = 5 biological replicates). Significant differences between the genotypes according to Duncan’s multiple range test (*p*-value ≤ 0.01) are indicated by asterisks: *p ≤ 0.05, **p ≤ 0.01.

The drought treatment resulted in significant increases in activities of all three studied antioxidant enzymes (SOD, POD, and CAT) in the Sirvan roots at all time points (except CAT at the 3 h time point) (**Figure 7**). Moreover, activities of all three enzymes increased with time in response to drought in this genotype. In contrast, no significant between-treatment differences were observed in antioxidant enzyme activities in Moghan3 roots at 3 h (**Figure 7**). In addition, although SOD, POD and CAT activities were significantly increased at later (24 and 48 h) time points of drought stress, their activities were considerably lower in Moghan3 than in Sirvan (**Figure 7**).

### 3.9 Physiochemical changes in response to drought

No significant changes were detected in the RWC of either wheat genotype after 3 h of the drought treatment (**Table 2**). At the 24 and 48 h time points, RWC was lower in both genotypes, but significantly higher in Sirvan than in Moghan3 (**Table 2**). The drought treatment also increased the proline content of roots of both wheat genotypes (**Table 2**), but it was higher in Sirvan than in Moghan3 at all time points except 3 h (**Table 2**). In both genotypes it was highest after 48 h of drought stress. H_2_O_2_ accumulated in both wheat genotypes under drought stress, but the accumulation was weaker in Sirvan than in Moghan3 at all time points (**Table 2**). The drought treatment significantly increased EL% in both genotypes, but there was less stress-derived damages (as reflected in EL%) in Sirvan than in Moghan3 (**Table 2**).

**Table 2:** Physiochemical properties of the Sirv_an_ and Moghan3 wheat genotypes measured under non-stressed and drought conditions.

	RWC%		Proline (µg g^-1^ fw)		H_2_O_2_ (µmol g^-1^ fw)		EL%	
	Sirvan	Moghan3	Sirvan	Moghan3	Sirvan	Moghan3	Sirvan	Moghan3
Non-stress	93.82^a^	93.74^a^	2.1^f^	2.05^f^	2.31^g^	2.23^g^	4.63^g^	4.67^g^
3 h	93.05^b^	92.89^b^	3.14^e^	3.08^e^	2.98^f^	3.42^e^	5.09^f^	5.84^e^
24 h	87.87^c^	83.65^d^	7.95^c^	6.64^d^	3.62^d^	4.78^c^	9.47^d^	11.09^c^
48 h	79.57^e^	70.35^f^	13.14^a^	10.32^b^	5.17^b^	8.67^a^	15.73^b^	19.35^a^

Different letters indicate significant differences in means according to the Duncan test (p-value ≤ 0.01).

Relative Water Content (RWC) was evaluated in their leaves while proline content, H_2_O_2_ content, and electrolyte leakage (EL) were measured in the roots..

### 3.10 Relationships among measured characteristics

**Figure 8:**
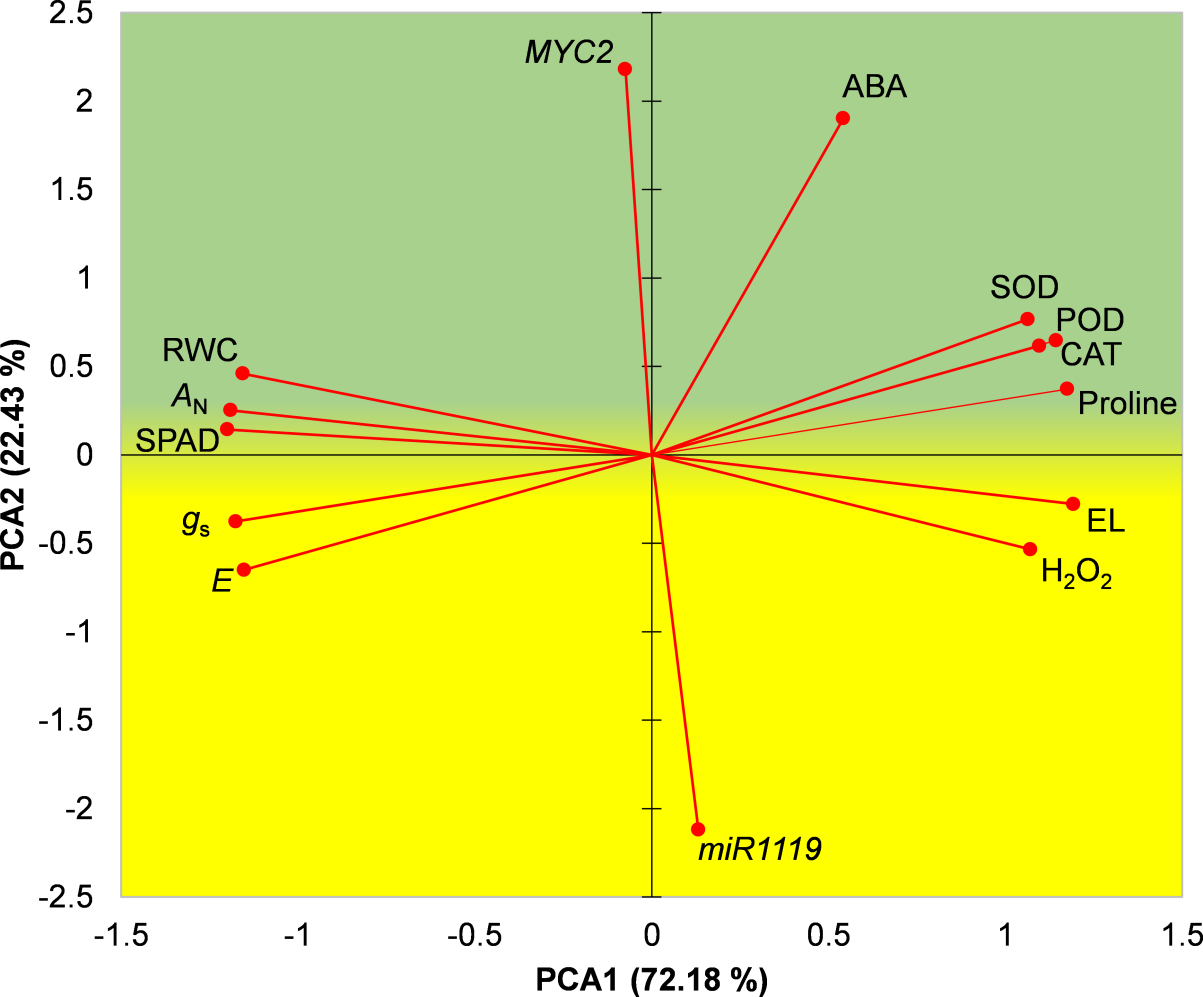
PCA biplot of measured characteristics of the Sirvan and Moghan3 wheat genotypes under drought conditions. The green and yellow regions correspond to drought stress tolerance and drought stress susceptibility, respectively. PCA1, first principal component; PCA2, second principal component.

A biplot of the first two principal components obtained in the PCA is shown in **Figure 8**. A biplot of the first two principal components obtained in the PCA is shown in **Figure 8**. Based on locations of drought-tolerant and drought-susceptible genotypes in the biplot it was divided into an upper ‘stress-tolerance’ (green) region and lower ‘stress-susceptibility’ (yellow) region where Sirvan and Moghan3 were located, respectively (**Figure 8**). *MYC2* expression, ABA content, activities of antioxidant enzymes, proline content, RWC, *A*_N_ and SPAD were located in the stress-tolerance region, indicating that high levels of these characteristics are important for drought tolerance in wheat. In contrast, *miR1119* expression, H_2_O_2_ content, EL%, *E*, and *g*_s_ were located in the stress-susceptibility region, indicating that high levels of these traits in drought conditions contribute to high susceptibility.

## 4 Discussion

Regulatory miRNA-target modules are essential contributors to plant stress responses and tolerance and provide an effective tool for manipulating crops’ stress tolerance (Zhang et al., 2022a). Systems biology approaches facilitate the identification of such regulatory modules in complex networks by combining computational and experimental methods (Cramer et al., 2011; Shamloo-Dashtpagerdi et al., 2022a). Thus, we adopted such an approach by combining analysis of EST sequences of wheat roots and physiochemical characteristics of wheat plants under drought and control conditions. The results provided new insights into drought responses of wheat, including the apparent involvement of a *miR1119-MYC2* module.

The first report on the responsiveness of *miR1119* of wheat to abiotic stresses was presented by Lu et al. (2011). A subsequent detailed investigation by Shi et al. (2018) revealed that under drought stress tobacco lines overexpressing wheat *miR1119* exhibited more drought tolerance than wild-type plants. They also showed that *miR1119* plays important roles in drought responses of both the monocot wheat and eudicot tobacco (Shi et al., 2018). Another study showed that *miR1119* is highly expressed in wheat roots and targets several *phenylalanine ammonia-lyase* (*PAL*) genes in wheat, which play key roles in plants’ adaptation, growth, and development (Rasool et al., 2021). However, despite the insights provided by such studies, many questions about *miR1119*’s function in wheat responses to drought and underlying physiochemical processes remained to be answered.

In wheat, numerous miRNAs are known to regulate expression of their targeted TFs, thus playing key roles in its drought tolerance mechanisms (Budak et al., 2015). Some of these miRNAs and their targets (shown in parentheses), which are differentially expressed in leaves of contrasting wheat genotypes under dehydration stress (Ma et al., 2015), included *miR159a,b* (*MYB3*), *miR156k* (*SBP*), *miR444c.1* (*MADS-box*), *miR160a* (*ARF*), *miR164b* (*NAC*), *miR166h* (*HD-ZIP4*), *miR169d* (*CCAAT-box TF*), *miR393b,i* (*TIR1*), *miR396a,c,g* (*GRF*), *miR444d* (*IF3*) and *miR827-5p* (*finger-like protein*). These miRNA-TF modules are involved in regulation of diverse drought tolerance pathways. For example, *miR159a,b*-MYB functions in auxin signalling and oligopeptide transport, *miR164b*-NAC participates in oxidative stress responses, *miR169d*-CCAAT-box regulates ABA-responsive transcription and miR397-Ice1 modulates responses to water deprivation (Budak et al., 2015). However, despite the versatility and importance of MYC TFs in plants’ growth, development, and environmental responses, there have been few reports regarding their functions in crop plants, especially economically important monocot crops, including wheat. In two previous studies, Chen et al. (2019) and Bai et al. (2019) respectively identified 26 and 27 non-redundant MYC TFs in the whole wheat genome. Specifically, expression profiling of identified *MYC* genes revealed that, like their dicot counterparts, the *MYC2* gene is differentially expressed in responses to differences in light quality, diverse hormone stimuli, and a wide array of abiotic stresses in wheat (Bai et al., 2019; Chen et al., 2019). These results clearly indicate that this gene participates in wheat’s response to abiotic stresses, including drought. However, to our knowledge no previously published studies provide information on miRNA-based regulation of MYC2 expression under abiotic stress conditions in any plants, except for suggestions by (Guo et al., 2017) that the *MYC2* gene may be a *miR172g-3p* target in phytohormone signalling in Oolong Tea (*Camellia sinensis* (L.) Kuntze). Thus, we believe that our study provides the first information on the relationship between *miR1119* and *MYC2* in wheat and the *miR1119*-*MYC2* module’s role under drought stress. Our computational analysis indicates with high confidence (expectation value 1.5) that *MYC2* is a potential target of *miR1119*, because with a stringent cut-off threshold (expectation ≤ 2), psRNATarget can identify target genes with high accuracy (up to 82%) and low false positive prediction rates (Dai and Zhao, 2011). Furthermore, studying the expression profile of miRNAs and their potential target genes might provide evidence about their functions. Our results show that the identified wheat *miR1119* and its predicted target gene *MYC2* were expressed differentially in response to drought treatments. Moreover, their expression differed between wheat genotypes with different degrees of drought tolerance, and there was a consistent negative correlation between the expression of *miR1119* and *MYC2* in both wheat genotypes. These findings strengthen computational predictions concerning *MYC2* as a putative target gene of *miR1119* and the hypothesis that *miR1119* and *MYC2* play important roles in wheat’s drought tolerance.

The transcription factor *MYC2* is a known master regulator of stress signalling (Song et al., 2022) that finetunes crosstalk between JA signalling pathways and those of other phytohormones, such as ABA, salicylic acid (SA), gibberellins (GAs), and auxin (IAA) (Kazan and Manners, 2013). *MYC2* participates in multiple complex processes in plants, including the regulation of biotic and abiotic stress responses, and their growth and development (Luo et al., 2023). Some biological processes related to drought responses of wheat in which *MYC2* is involved are shown in the gene network based on pathway analysis (**Figure 4**). *MYC2* targets a wide range of downstream genes (Kazan and Manners, 2013; López-Vidriero et al., 2021), so has expected involvement in several drought response processes. For example, *ADH1* (*ALCOHOL DEHYDROGENASE 1*), one of the *MYC2* target genes (Abe et al., 2003) that was present in the identified DEGs and predicted gene network (**Figure 4**), is reportedly involved in tolerance of biotic and abiotic stresses, including drought (Shi et al., 2017; Su et al., 2020). Our pathway analysis, physiochemical assays, and statistical analysis illuminate how the *miR1119*-*MYC2* module may contribute to wheat’s drought tolerance. PCA indicated that *miR1119* and *MYC2* expression is negatively and positively associated with ABA content, respectively. Moreover, ABA accumulation was faster and stronger under drought in the tolerant wheat genotype, Sirvan, than in the susceptible genotype, Moghan3. This is consistent with expectations, as regulation of ABA biosynthesis is one of the fastest responses of plants to abiotic stresses such as drought and salinity (Peleg and Blumwald, 2011), and upregulation of ABA biosynthetic genes in drought stress conditions has been observed in several plant species (Vishwakarma et al., 2017). It has also been demonstrated that *MYC2* positively regulates ABA biosynthesis and signalling (Verma et al., 2020), and that drought priming treatments induce drought tolerance in wheat by upregulating genes involved in ABA biosynthesis, including *MYC2* (Wang et al., 2021). Furthermore, *MYC2* positively regulates the transcription of *ABA2* and *NCED3* genes encoding two key enzymes in the ABA biosynthesis cascade (Verma et al., 2020). These observations, along with our results, support the hypothesis that *MYC2* is involved in wheat’s ABA biosynthesis and hence drought stress responses and tolerance. However, further research is needed to elucidate fully the participants in regulatory mechanisms of ABA biosynthesis in wheat roots involving *MYC2* and their precise roles.

ABA fundamentally regulates plants’ morphological and physiological activities in response to drought stress (Mukarram et al., 2021). We found that ABA content was positively associated with RWC, and negatively associated with *g*_s_, and *E* in wheat. In addition, RWC was significantly higher (but *g*_s_ and *E* lower) in Sirvan than in Moghan3. Under short-term water deficit, as in our experiment, ABA regulates stomatal closure and modulates mesophyll conductance without restraining CO_2_ fixation (Sorrentino et al., 2016; Brunetti et al., 2019). Accordingly, higher RWC during drought conditions leads to efficient photosynthesis activity (Aliakbari et al., 2021), which is reflected in more *A*_N_ in the tolerant genotype. This finding was further confirmed as our results showed that the tolerant genotype Sirvan maintained higher WUEi, i.e., higher ratios of *A*_N_ to *g*_s_. Improving WUE is an important subject to alleviate the deleterious effects of drought stress and to maintain a higher grain yield of wheat (Ahmed et al., 2019). Therefore, unsurprisingly, water uptake, transport, and loss seem to play key roles in wheat’s drought tolerance. Besides the role of ABA in leaf stomata aperture, which regulates WUE and energy partitioning of crops (Yu et al., 2004), previous studies have also shown that ABA regulates root hydraulic conductance (*L*p), resulting in adjustments of plants’ water relations and increases in WUE during drought (Kuromori et al., 2018). Moreover, increases in levels of endogenous ABA in roots of plants under drought stress boost adaptive responses, including increases in root elongation and reductions in lateral root formation, that increase root water uptake efficiency (Kuromori et al., 2018; Maurel and Nacry, 2020). Altogether, it can be postulated that the upregulation of *MYC2* due to the downregulation of *miR1119* in roots, and consequently greater ABA accumulation, contribute to increases in WUE and drought tolerance of wheat.

Responses to drought include ROS bursts induced by osmotic stress, which cause membrane oxidation and damage (Sachdev et al., 2021; Mansoor et al., 2022). Both enzymatic mechanisms (involving SOD, POD and CAT) and non-enzymatic mechanisms (involving proline and various antioxidants) detoxify ROS, maintain cellular ROS homeostasis (to varying genotype- and stress level-dependent degrees), and contribute to plants’ overall drought tolerance (Laxa et al., 2019; Shamloo-Dashtpagerdi et al., 2022; Shamloo-Dashtpagerdi et al., 2022a). ABA plays a major role in the induction of such adaptive responses in wheat to stresses, including drought (Bano et al., 2012; Yu et al., 2020; Wang et al., 2021). Activities of SOD, POD, and CAT enzymes and the accumulation of proline were all located in the tolerant region of the PCA biplot, and positively correlated to both *MYC2* expression and ABA content. In addition, the SPAD, EL% and H_2_O_2_ measurements indicate that chlorophyll degradation and membrane damage levels were lower in Sirvan than in Moghan3 under drought stress. Thus, Sirvan has more potent antioxidative activities than Moghan3 in drought conditions. Under salinity stress, *MYC2*-*like* overexpression reportedly enhances salt tolerance in transformants through several physiochemical responses, including increases in proline accumulation and SOD, POD, and CAT activities (Liu et al., 2022). Similarly, *TaMYC2* responds to abiotic (cold, drought, alkali and salt) stresses and increases tolerance in Caucasian Clover (*Trifolium ambiguum* M. Bieb.) by increasing antioxidant enzymes’ activities (Zhao et al., 2022). It has also found that *TaMYC2* overexpression significantly improved drought and cold tolerance of transgenic tobacco by increasing these activities. Similarly, MeJA, NaCl, and PEG treatments significantly increase *SmMYC2* expression in *Salvia miltiorrhiza* Bunge (Deng et al., 2022). Furthermore, overexpression of *SmMYC2* in transgenic Arabidopsis plants increased their SOD, POD, CAT enzyme activities, and proline content, but reduced their malondialdehyde (MDA) levels and ROS accumulation, indicating that it could enhance their slat tolerance (Deng et al., 2022). Accordingly, our results show that *MYC2* also confers protection against ROS accumulation by regulating antioxidant enzymes’ activities and proline accumulation in wheat roots and indicate that genotype-dependent increases in *MYC2* expression linked to the decrease in *mir1119* expression may contribute to wheat’s drought tolerance.

## 5 Conclusion

We have used a combination of computational, statistical, and experimental methods to investigate the potential miRNA-target gene modules involved in wheat’s drought responses and tolerance. Accordingly, we found that *miR1119* significantly contributes to wheat responses to drought stress. We also found clear associations between the expression patterns of the *miR1119*-*MYC2* module and a wide array of physiochemical changes of wheat under drought stress conditions. The module is differentially regulated in two contrasting wheat genotypes. It may contribute to wheat plants’ drought tolerance by adjusting their water relations and alleviating oxidative stress damage by regulating activities of antioxidant enzymes and proline content. Therefore, it may be a key biomarker of levels of wheat genotypes’ drought tolerance. Further functional characterization of *miR1119* and its target gene would provide more concrete evidence of the roles of these modules in regulating drought tolerance.

## Data availability statement

The original contributions presented in the study are included in the article/**Supplementary Material**. Further inquiries can be directed to the corresponding authors.

## Author contributions

RS-D conceived and planned the study, performed computational transcriptome analysis, statistically analysed the results and wrote the manuscript draft and discussion, AGS and AT carried out the drought experiment and wrote some parts of the manuscript. RRV supported the project and developed the methodology. All authors contributed to the article and approved the submitted version.

## References

[B1] Abe H. Urao T. Ito T. Seki M. Shinozaki K. Yamaguchi-Shinozaki K. (2003). Arabidopsis AtMYC2 (bHLH) and AtMYB2 (MYB) function as transcriptional activators in abscisic acid signaling. Plant Cell 15, 63–78. doi: 10.1105/tpc.006130 12509522 PMC143451

[B2] Ahmad Z. Waraich E. A. Akhtar S. Anjum S. Ahmad T. Mahboob W.. (2018). Physiological responses of wheat to drought stress and its mitigation approaches. Acta Physiologiae Plantarum 40, 1–13. doi: 10.1007/s11738-018-2651-6

[B3] Ahmed N. Zhang Y. Li K. Zhou Y. Zhang M. Li Z. (2019). Exogenous application of glycine betaine improved water use efficiency in winter wheat (*Triticum aestivum l.*) *via* modulating photosynthetic efficiency and antioxidative capacity under conventional and limited irrigation conditions. Crop J. 7, 635–650. doi: 10.1016/j.cj.2019.03.004

[B4] Aliakbari M. Cohen S. P. Lindlöf A. Shamloo-Dashtpagerdi R. J. P. P. (2021). Rubisco activase a (RcaA) is a central node in overlapping gene network of drought and salinity in barley (*Hordeum vulgare* l.) and may contribute to combined stress tolerance. Plant Physiol. Biochem. 161, 248–258. doi: 10.1016/j.plaphy.2021.02.016 33652257

[B5] Anjali N. Nadiya F. Thomas J. Sabu K. (2019). Identification and characterization of drought responsive microRNAs and their target genes in cardamom *(Elettaria cardamomum maton)* . Plant Growth Regul. 87, 201–216. doi: 10.1007/s10725-018-0462-9

[B6] Arkhipova T. N. Evseeva N. V. Tkachenko O. V. Burygin G. L. Vysotskaya L. B. Akhtyamova Z. A.. (2020). Rhizobacteria inoculation effects on phytohormone status of potato microclones cultivated *in vitro* under osmotic stress. Biomolecules 10, 1231. doi: 10.3390/biom10091231 32847137 PMC7564303

[B7] Audic S. Claverie J.-M. (1997). The significance of digital gene expression profiles. Genome Res. 7, 986–995. doi: 10.1101/gr.7.10.986 9331369

[B8] Axtell M. J. Meyers B. C. (2018). Revisiting criteria for plant microRNA annotation in the era of big data. Plant Cell 30, 272–284. doi: 10.1105/tpc.17.00851 29343505 PMC5868703

[B9] Bai J.-F. Wang Y.-K. Guo L.-P. Guo X.-M. Guo H.-Y. Yuan S.-H.. (2019). Genomic identification and characterization of MYC family genes in wheat (*Triticum aestivum* l.). BMC Genomics 20, 1–15. doi: 10.1186/s12864-019-6373-y 31888472 PMC6937671

[B10] Baltoumas F. A. Zafeiropoulou S. Karatzas E. Koutrouli M. Thanati F. Voutsadaki K.. (2021). Biomolecule and bioentity interaction databases in systems biology: a comprehensive review. Biomolecules 11, 1245. doi: 10.3390/biom11081245 34439912 PMC8391349

[B11] Bano A. Ullah F. Nosheen A. (2012). Role of abscisic acid and drought stress on the activities of antioxidant enzymes in wheat. Plant Soil Environ. 58, 181–185. doi: 10.17221/210/2011-PSE

[B12] Bates L. S. Waldren R. P. Teare I. (1973). Rapid determination of free proline for water-stress studies. Plant Soil 39, 205–207. doi: 10.1007/BF00018060

[B13] Benjamini Y. Hochberg Y. (1995). Controlling the false discovery rate: a practical and powerful approach to multiple testing. J. R. Stat. Society. Ser. B (Methodological), 57(1):289–300. doi: 10.1111/j.2517-6161.1995.tb02031.x

[B14] Bouck A. Vision T. J. M. E. (2007). The molecular ecologist's guide to expressed sequence tags. Mol. Ecol. 16, 907–924. doi: 10.1111/j.1365-294X.2006.03195.x 17305850

[B15] Brunetti C. Gori A. Marino G. Latini P. Sobolev A. P. Nardini A.. (2019). Dynamic changes in ABA content in water-stressed populus nigra: effects on carbon fixation and soluble carbohydrates. Ann. Bot. 124, 627–643. doi: 10.1093/aob/mcz005 30715123 PMC6821382

[B16] Budak H. Hussain B. Khan Z. Ozturk N. Z. Ullah N. (2015). From genetics to functional genomics: improvement in drought signaling and tolerance in wheat. Front. Plant Sci. 6, 1012. doi: 10.3389/fpls.2015.01012 26635838 PMC4652017

[B17] Chao S. Lazo G. You F. Crossman C. Hummel D. Lui N.. (2006). Use of a large-scale triticeae expressed sequence tag resource to reveal gene expression profiles in hexaploid wheat (*Triticum aestivum* l.). Genome 49, 531–544. doi: 10.1139/g06-003 16767178

[B18] Chen S. Zhao H. Luo T. Liu Y. Nie X. Li H. (2019). Characteristics and expression pattern of MYC genes in *triticum aestivum*, oryza sativa, and brachypodium distachyon. Plants 8, 274. doi: 10.3390/plants8080274 31398900 PMC6724133

[B19] Claverie J.-M. Ta T. N. (2019). ACDtool: a web-server for the generic analysis of large data sets of counts. Bioinformatics 35, 170–171. doi: 10.1093/bioinformatics/bty640 30020402

[B20] Corner S. (2009). Choosing the right type of rotation in PCA and EFA. JALT testing Eval. SIG Newslett. 13, 20–25.

[B21] Cramer G. R. Urano K. Delrot S. Pezzotti M. Shinozaki K. (2011). Effects of abiotic stress on plants: a systems biology perspective. BMC Plant Biol. 11, 1–14. doi: 10.1186/1471-2229-11-163 22094046 PMC3252258

[B22] Dai X. Zhao P. X. (2011). psRNATarget: a plant small RNA target analysis server. Nucleic Acids Res. 39, W155–W159. doi: 10.1093/nar/gkr319 21622958 PMC3125753

[B23] Dai X. Zhuang Z. Zhao P. X. (2018). psRNATarget: a plant small RNA target analysis server (2017 Release). Nucleic Acids Res. 46, W49–W54. doi: 10.1093/nar/gky316 29718424 PMC6030838

[B24] Deng H. Li Q. Cao R. Ren Y. Wang G. Guo H.. (2022). Overexpression of SmMYC2 enhances salt resistance in arabidopsis thaliana and salvia miltiorrhiza hairy roots. J. Plant Physiol. 280, 153862. doi: 10.1016/j.jplph.2022.153862 36399834

[B25] Figueroa-Bustos V. Palta J. A. Chen Y. Stefanova K. Siddique K. H. (2020). Wheat cultivars with contrasting root system size responded differently to terminal drought. Front. Plant Sci. 11, 1285. doi: 10.3389/fpls.2020.01285 32973844 PMC7466772

[B26] Franco-Navarro J. D. Brumós J. Rosales M. A. Cubero-Font P. Talón M. Colmenero-Flores J. M. (2016). Chloride regulates leaf cell size and water relations in tobacco plants. J. Exp. Bot. 67, 873–891. doi: 10.1093/jxb/erv502 26602947 PMC4737079

[B27] Garcia-Moreno A. López-Domínguez R. Villatoro-García J. A. Ramirez-Mena A. Aparicio-Puerta E. Hackenberg M.. (2022). Functional enrichment analysis of regulatory elements. Biomedicines 10, 590. doi: 10.3390/biomedicines10030590 35327392 PMC8945021

[B28] Guigon I. Legrand S. Berthelot J.-F. Bini S. Lanselle D. Benmounah M.. (2019). miRkwood: a tool for the reliable identification of microRNAs in plant genomes. BMC Genomics 20, 1–9. doi: 10.1186/s12864-019-5913-9 31253093 PMC6599362

[B29] Guo Y. Zhao S. Zhu C. Chang X. Yue C. Wang Z.. (2017). Identification of drought-responsive miRNAs and physiological characterization of tea plant (Camellia sinensis l.) under drought stress. BMC Plant Biol. 17, 1–20. doi: 10.1186/s12870-017-1172-6 29157225 PMC5696764

[B30] Hou J. Lu D. Mason A. S. Li B. Xiao M. An S.. (2019). Non-coding RNAs and transposable elements in plant genomes: emergence, regulatory mechanisms and roles in plant development and stress responses. Planta 250, 23–40. doi: 10.1007/s00425-019-03166-7 30993403

[B31] Ilyas M. Nisar M. Khan N. Hazrat A. Khan A. H. Hayat K.. (2021). Drought tolerance strategies in plants: a mechanistic approach. J. Plant Growth Regul. 40, 926–944. doi: 10.1007/s00344-020-10174-5

[B32] Juliana P. Poland J. Huerta-Espino J. Shrestha S. Crossa J. Crespo-Herrera L.. (2019). Improving grain yield, stress resilience and quality of bread wheat using large-scale genomics. Nat. Genet. 51, 1530–1539. doi: 10.1038/s41588-019-0496-6 31548720

[B33] Kazan K. Manners J. M. (2013). MYC2: the master in action. Mol. Plant 6, 686–703. doi: 10.1093/mp/sss128 23142764

[B34] Kuromori T. Seo M. Shinozaki K. (2018). ABA transport and plant water stress responses. Trends Plant Sci. 23, 513–522. doi: 10.1016/j.tplants.2018.04.001 29731225

[B35] Laxa M. Liebthal M. Telman W. Chibani K. Dietz K.-J. (2019). The role of the plant antioxidant system in drought tolerance. Antioxidants 8, 94. doi: 10.3390/antiox8040094 30965652 PMC6523806

[B36] Liu H. Cui P. Zhang B. Zhu J. Liu C. Li Q. (2022). Binding of the transcription factor MYC2-like to the ABRE of the OsCYP2 promoter enhances salt tolerance in oryza sativa. PLoS One 17, e0276075. doi: 10.1371/journal.pone.0276075 36240213 PMC9565382

[B37] Liu X. Zhang X. Sun B. Hao L. Liu C. Zhang D.. (2019). Genome-wide identification and comparative analysis of drought-related microRNAs in two maize inbred lines with contrasting drought tolerance by deep sequencing. PLoS One 14, e0219176. doi: 10.1371/journal.pone.0219176 31276526 PMC6611575

[B38] Livak K. J. Schmittgen T. D. (2001). Analysis of relative gene expression data using real-time quantitative PCR and the 2– ΔΔCT method. Methods 25, 402–408. doi: 10.1006/meth.2001.1262 11846609

[B39] López-Vidriero I. Godoy M. Grau J. Peñuelas M. Solano R. Franco-Zorrilla J. M. (2021). DNA Features beyond the transcription factor binding site specify target recognition by plant MYC2-related bHLH proteins. Plant Commun. 2, 100232. doi: 10.1016/j.xplc.2021.100232 34778747 PMC8577090

[B40] Lu W. Li J. Liu F. Gu J. Guo C. Xu L.. (2011). Expression pattern of wheat miRNAs under salinity stress and prediction of salt-inducible miRNAs targets. Front. Agric. China 5, 413–422. doi: 10.1007/s11703-011-1133-z

[B41] Luo L. Wang Y. Qiu L. Han X. Zhu Y. Liu L.. (2023). MYC2: a master switch for plant physiological processes and specialized metabolite synthesis. Int. J. Mol. Sci. 24, 3511. doi: 10.3390/ijms24043511 36834921 PMC9963318

[B42] Ma X. Xin Z. Wang Z. Yang Q. Guo S. Guo X.. (2015). Identification and comparative analysis of differentially expressed miRNAs in leaves of two wheat (*Triticum aestivum* l.) genotypes during dehydration stress. BMC Plant Biol. 15, 1–15. doi: 10.1186/s12870-015-0413-9 25623724 PMC4312605

[B43] Mansoor S. Ali Wani O. Lone J. K. Manhas S. Kour N. Alam P.. (2022). Reactive oxygen species in plants: from source to sink. Antioxidants 11, 225. doi: 10.3390/antiox11020225 35204108 PMC8868209

[B44] Masoudi-Nejad A. Tonomura K. Kawashima S. Moriya Y. Suzuki M. Itoh M.. (2006). EGassembler: online bioinformatics service for large-scale processing, clustering and assembling ESTs and genomic DNA fragments. Nucleic Acids Res. 34, W459–W462. doi: 10.1093/nar/gkl066 16845049 PMC1538775

[B45] Maurel C. Nacry P. (2020). Root architecture and hydraulics converge for acclimation to changing water availability. Nat. Plants 6, 744–749. doi: 10.1038/s41477-020-0684-5 32601421

[B46] Mehrabad Pour-Benab S. Fabriki-Ourang S. Mehrabi A.-A. (2019). Expression of dehydrin and antioxidant genes and enzymatic antioxidant defense under drought stress in wild relatives of wheat. Biotechnol. Biotechnol. Equip. 33, 1063–1073. doi: 10.1080/13102818.2019.1638300

[B47] Millar A. A. (2020). The function of miRNAs in plants. Plants 9, 198. doi: 10.3390/plants9020198 32033453 PMC7076417

[B48] Mukarram M. Choudhary S. Kurjak D. Petek A. Khan M. M. A. (2021). Drought: sensing, signalling, effects and tolerance in higher plants. Physiologia Plantarum 172, 1291–1300. doi: 10.1111/ppl.13423 33847385

[B49] Nikitin A. Egorov S. Daraselia N. Mazo I. (2003). Pathway studio–the analysis and navigation of molecular networks. Bioinformatics 19, 2155–2157. doi: 10.1093/bioinformatics/btg290 14594725

[B50] Nogoy F. M. Niño M. C. Song J. Y. Jung Y. J. Kang K. K. Nou I.. (2018). Plant microRNAs in molecular breeding. Plant Biotechnol. Rep. 12, 15–25. doi: 10.1007/s11816-018-0468-9

[B51] Pagano L. Rossi R. Paesano L. Marmiroli N. Marmiroli M. (2021). miRNA regulation and stress adaptation in plants. Environ. Exp. Bot. 184, 104369. doi: 10.1016/j.envexpbot.2020.104369

[B52] Patra H. Kar M. Mishra D.J.B.U.P.D.P. (1978). Catalase activity in leaves and cotyledons during plant development and senescence. Biochemie und Physiologie der Pflanzen 172, 385–390. doi: 10.1016/S0015-3796(17)30412-2

[B53] Peleg Z. Blumwald E. (2011). Hormone balance and abiotic stress tolerance in crop plants. Curr. Opin. Plant Biol. 14, 290–295. doi: 10.1016/j.pbi.2011.02.001 21377404

[B54] Pereira A. (2016). Plant abiotic stress challenges from the changing environment. Front. Plant Sci. 7, 1123. doi: 10.3389/fpls.2016.01123 27512403 PMC4962010

[B55] Rane J. Singh A. K. Tiwari M. Prasad P. Jagadish S. (2022). Effective use of water in crop plants in dryland agriculture: implications of reactive oxygen species and antioxidative system. Front. Plant Sci. 12 3098. doi: 10.3389/fpls.2021.778270 PMC8784697 35082809

[B56] Rasool F. Uzair M. Naeem M. K. Rehman N. Afroz A. Shah H.. (2021). Phenylalanine ammonia-lyase (PAL) genes family in wheat (*Triticum aestivum* l.): genome-wide characterization and expression profiling. Agronomy 11, 2511. doi: 10.3390/agronomy11122511

[B57] Riasat M. Pessarakli M. Niaz A. A. Saed-Moucheshi A.J.J.O.P.N. (2018). Assessment of different wheat genotypes with altered genetic background in response to different salinity levels. J. Plant Nutr. 41, 1821–1833. doi: 10.1080/01904167.2018.1462383

[B58] Sachdev S. Ansari S. A. Ansari M. I. Fujita M. Hasanuzzaman M. (2021). Abiotic stress and reactive oxygen species: generation, signaling, and defense mechanisms. Antioxidants 10, 277. doi: 10.3390/antiox10020277 33670123 PMC7916865

[B59] Sade N. Galkin E. Moshelion M. (2015). Measuring arabidopsis, tomato and barley leaf relative water content (RWC). Bio-protocol 5, e1451–e1451. doi: 10.21769/BioProtoc.1451

[B60] Sairam R. Srivastava G. (2002). Changes in antioxidant activity in sub-cellular fractions of tolerant and susceptible wheat genotypes in response to long term salt stress. Plant Sci. 162, 897–904. doi: 10.1016/S0168-9452(02)00037-7

[B61] Sallam A. Alqudah A. M. Dawood M. F. Baenziger P. S. Börner A. (2019). Drought stress tolerance in wheat and barley: advances in physiology, breeding and genetics research. Int. J. Mol. Sci. 20 3137. doi: 10.3390/ijms20133137 31252573 PMC6651786

[B62] Shah K. Nahakpam S. J. P. P. (2012). Heat exposure alters the expression of SOD, POD, APX and CAT isozymes and mitigates low cadmium toxicity in seedlings of sensitive and tolerant rice cultivars. Plant Physiol. Biochem. 57, 106–113. doi: 10.1016/j.plaphy.2012.05.007 22698753

[B63] Shamloo-Dashtpagerdi R. Aliakbari M. Lindlöf A. Tahmasebi S. (2022a). A systems biology study unveils the association between a melatonin biosynthesis gene, O-methyl transferase 1 (OMT1) and wheat (*Triticum aestivum* l.) combined drought and salinity stress tolerance. Planta 255, 1–15. doi: 10.1007/s00425-022-03885-4 35386021

[B64] Shamloo-Dashtpagerdi R. Lindlöf A. Niazi A. Pirasteh-Anosheh H. (2019). LOS2 gene plays a potential role in barley (*Hordeum vulgare* l.) salinity tolerance as a hub gene. Mol. Breed. 39, 119. doi: 10.1007/s11032-019-1026-z

[B65] Shamloo-Dashtpagerdi R. Lindlöf A. Tahmasebi S. (2022). Evidence that miR168a contributes to salinity tolerance of brassica rapa l. *via* mediating melatonin biosynthesis. Physiologia Plantarum 174, e13790. doi: 10.1111/ppl.13790 36169653

[B66] Shamloo-Dashtpagerdi R. Razi H. Alemzadeh A. Ebrahimie E. (2022b). Further insights into the association of the protein phosphatase gene ABI1 with drought and salinity stress responses in brassica species. J. Plant Biochem. Biotechnol., 32(1):1–15. doi: 10.1007/s13562-022-00786-1

[B67] Shamloo-Dashtpagerdi R. Sisakht J. N. Tahmasebi A. (2022c). MicroRNA miR1118 contributes to wheat (*Triticum aestivum* l.) salinity tolerance by regulating the plasma membrane intrinsic Proteins1; 5 (PIP1; 5) gene. J. Plant Physiol. 278, 153827. doi: 10.1016/j.jplph.2022.153827 36206620

[B68] Shi G.-Q. Fu J.-Y. Rong L.-J. Zhang P.-Y. Guo C.-J. Kai X. (2018). TaMIR1119, a miRNA family member of wheat (*Triticum aestivum*), is essential in the regulation of plant drought tolerance. J. Integr. Agric. 17, 2369–2378. doi: 10.1016/S2095-3119(17)61879-3

[B69] Shi H. Liu W. Yao Y. Wei Y. Chan Z. (2017). Alcohol dehydrogenase 1 (ADH1) confers both abiotic and biotic stress resistance in arabidopsis. Plant Sci. 262, 24–31. doi: 10.1016/j.plantsci.2017.05.013 28716417

[B70] Shinozaki K. Yamaguchi-Shinozaki K. (2007). Gene networks involved in drought stress response and tolerance. J. Exp. Bot. 58, 221–227. doi: 10.1093/jxb/erl164 17075077

[B71] Song C. Cao Y. Dai J. Li G. Manzoor M. A. Chen C.. (2022). The multifaceted roles of MYC2 in plants: towards transcriptional reprogramming and stress tolerance by JA signaling. Front. Plant Sci. 989. doi: 10.3389/fpls.2022.868874 PMC9082941 35548315

[B72] Sorrentino G. Haworth M. Wahbi S. Mahmood T. Zuomin S. Centritto M. (2016). Abscisic acid induces rapid reductions in mesophyll conductance to carbon dioxide. PloS One 11, e0148554. doi: 10.1371/journal.pone.0148554 26862904 PMC4749297

[B73] Stewart R. R. Bewley J.D.J.P.P. (1980). Lipid peroxidation associated with accelerated aging of soybean axes. Plant Physiol. 65, 245–248. doi: 10.1104/pp.65.2.245 16661168 PMC440305

[B74] Su W. Ren Y. Wang D. Su Y. Feng J. Zhang C.. (2020). The alcohol dehydrogenase gene family in sugarcane and its involvement in cold stress regulation. BMC Genomics 21, 1–17. doi: 10.1186/s12864-020-06929-9 PMC7392720 32727370

[B75] Tiwari M. Pandey V. Singh B. Bhatia S. (2021a). Dynamics of miRNA mediated regulation of legume symbiosis. Plant Cell Environ. 44, 1279–1291. doi: 10.1111/pce.13983 33347631

[B76] Tiwari M. Singh B. Yadav M. Pandey V. Bhatia S. (2021b). High throughput identification of miRNAs reveal novel interacting targets regulating chickpea-rhizobia symbiosis. Environ. Exp. Bot. 186, 104469. doi: 10.1016/j.envexpbot.2021.104469

[B77] Varkonyi-Gasic E. Wu R. Wood M. Walton E. F. Hellens R. P. (2007). Protocol: a highly sensitive RT-PCR method for detection and quantification of microRNAs. Plant Methods 3, 1–12. doi: 10.1186/1746-4811-3-12 17931426 PMC2225395

[B78] Verma D. Bhagat P. K. Sinha A. K. (2020). MKK3-MPK6-MYC2 module positively regulates ABA biosynthesis and signalling in arabidopsis. J. Plant Biochem. Biotechnol. 29, 785–795. doi: 10.1007/s13562-020-00621-5

[B79] Vishwakarma K. Upadhyay N. Kumar N. Yadav G. Singh J. Mishra R. K.. (2017). Abscisic acid signaling and abiotic stress tolerance in plants: a review on current knowledge and future prospects. Front. Plant Sci. 8, 161. doi: 10.3389/fpls.2017.00161 28265276 PMC5316533

[B80] Vysotskaya L. B. Arkhipova T. N. Timergalina L. N. Dedov A. V. Veselov S. Y. Kudoyarova G. R. (2004). Effect of partial root excision on transpiration, root hydraulic conductance and leaf growth in wheat seedlings. Plant Physiol. Biochem. 42, 251–255. doi: 10.1016/j.plaphy.2004.01.004 15051049

[B81] Wang X. Li Q. Xie J. Huang M. Cai J. Zhou Q.. (2021). Abscisic acid and jasmonic acid are involved in drought priming-induced tolerance to drought in wheat. Crop J. 9, 120–132. doi: 10.1016/j.cj.2020.06.002

[B82] Yu J. Cang J. Lu Q. Fan B. Xu Q. Li W.. (2020). ABA enhanced cold tolerance of wheat ‘dn1’via increasing ROS scavenging system. Plant Signaling Behav. 15, 1780403. doi: 10.1080/15592324.2020.1780403 PMC8570709 32619128

[B83] Yu Q. Zhang Y. Liu Y. Shi P. (2004). Simulation of the stomatal conductance of winter wheat in response to light, temperature and CO2 changes. Ann. Bot. 93, 435–441. doi: 10.1093/aob/mch023 14980969 PMC4242327

[B84] Yu Y. Zhou W. Liang X. Zhou K. Lin X. J. E. P. (2019). Increased bound putrescine accumulation contributes to the maintenance of antioxidant enzymes and higher aluminum tolerance in wheat. Environ. pollut. 252, 941–949. doi: 10.1016/j.envpol.2019.06.045 31252132

[B85] Zhang D. Choi D. Wanamaker S. Fenton R. Chin A. Malatrasi M.. (2004). Construction and evaluation of cDNA libraries for large-scale expressed sequence tag sequencing in wheat (*Triticum aestivum* l.). Genetics 168, 595–608. doi: 10.1534/genetics.104.034785 15514038 PMC1448820

[B86] Zhang B. Pan X. Cox S. Cobb G. Anderson T. (2006). Evidence that miRNAs are different from other RNAs. Cell. Mol. Life Sci. CMLS 63, 246–254. doi: 10.1007/s00018-005-5467-7 16395542 PMC11136112

[B87] Zhang F. Yang J. Zhang N. Wu J. Si H. (2022a). Roles of microRNAs in abiotic stress response and characteristics regulation of plant. Front. Plant Sci. 13, 919243. doi: 10.3389/fpls.2022.919243 36092392 PMC9459240

[B88] Zhang H. Zhu J. Gong Z. Zhu J.-K. (2022b). Abiotic stress responses in plants. Nat. Rev. Genet. 23, 104–119. doi: 10.1038/s41576-021-00413-0 34561623

[B89] Zhao Y. Yang Y. Jiang J. Zhang X. Ma Z. Meng L.. (2022). The caucasian clover gene TaMYC2 responds to abiotic stress and improves tolerance by increasing the activity of antioxidant enzymes. Genes 13, 329. doi: 10.3390/genes13020329 35205373 PMC8871790

