# Supplementary figures and images for: Potential role of the regulatory miR1119-MYC2 module in wheat (Triticum aestivum L.) drought tolerance
Source: Front Plant Sci. 2023 May 30;14:1161245. doi: 10.3389/fpls.2023.1161245 (PMC10266357; doi:10.3389/fpls.2023.1161245)

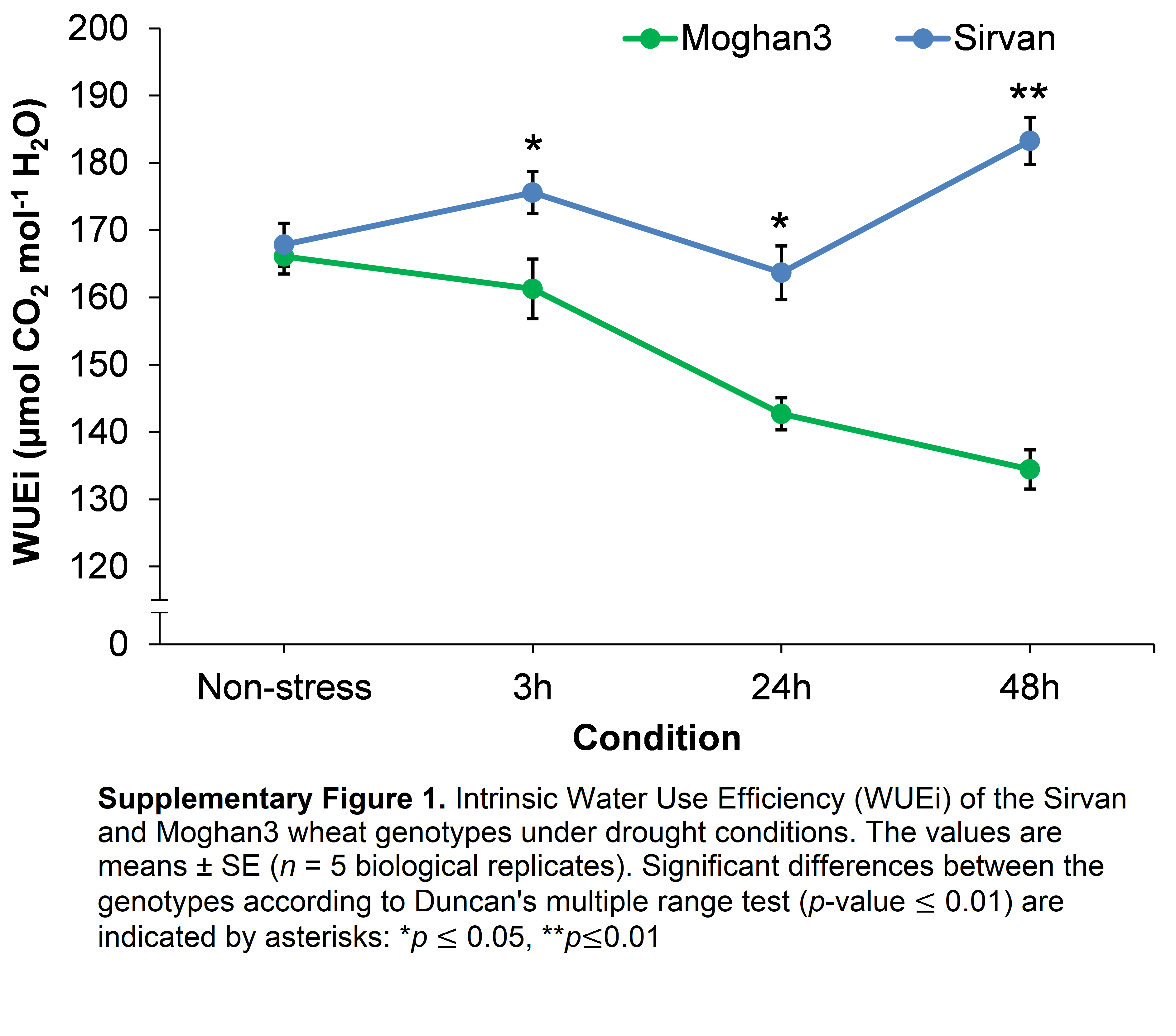

Supplement: Supplementary file 1 [file Image_1.tif]
