# Supplementary material for: Potential role of the regulatory miR1119-MYC2 module in wheat (Triticum aestivum L.) drought tolerance
Source: Front Plant Sci. 2023 May 30;14:1161245. doi: 10.3389/fpls.2023.1161245 (PMC10266357; doi:10.3389/fpls.2023.1161245)
Supplement: Supplementary file 2 [file Table_1.docx]

**Table S1**

List of primers used in qRT-PCR assays

| **Gene** | **Primer** |
| --- | --- |
| ***MYC2*** | F 5^′^- TGCTCTGGCTCGGCGTCAC-3^′^  R 5^′^-TCTCCGTGAGCGTGCTGGTG-3^′^ |
| ***Actin*** | F 5^′^-TTCTGTCCTTGTATGCCAGC-3^′^  R 5^′^-CATTAGATTATCCGTGAGGTC-3^′^ |
| ***miR1119*** | F 5′-GCGGCTGGCTCGGCGTGATGCTG-3′  RT 5′-GTCGTATCCAGTGCAGGGTCCGAGGTATTCGCACTGGATACGACCTGACT-3′ |
| ***rRNA 26*** | F 5′-CCGGTTGTTATGCCAATAGCA-3′  R 5′-GCGGCGCAGCAGTTCT-3′ |
