# Supplementary material for: Potential role of the regulatory miR1119-MYC2 module in wheat (Triticum aestivum L.) drought tolerance
Source: Front Plant Sci. 2023 May 30;14:1161245. doi: 10.3389/fpls.2023.1161245 (PMC10266357; doi:10.3389/fpls.2023.1161245)
Supplement: Supplementary file 5 [file Table_4.docx]

**Supplementary 1**

Fasta sequences of the identified differentially expressed genes

>Contig87

CTTAACCACACTCATCTGATCACCCACTAGCTAGCCAGCTAGCAGCTTAAGGAACTAGCT

TGCAGCTAGCGATCCATGGCTCGTCTCAACAGCAAGGCTGTGGCGGCCGCCGTGGTCCTG

GCGGCGGTGGTGCTGATGATGGCCGGCAGGGAGGCCTCGGCGGCGCTGTCGTGCGGGCAG

GTGGACTCCAAGCTCGCGCCGTGCGTGGCGTACGTGACGGGGAGGGCGTCCTCGATCAGC

AAGGAGTGCTGCTCCGGCGTGCAGGGGCTGAACGGCCTGGCCCGCAGCAGCCCGGACCGC

AAGATAGCGTGCAGGTGCCTCAAGAGCCTCGCCACCAGCATCAAGTCCATCAACATGGGC

AAGGTCTCCGGCGTGCCCGGCAAGTGCGGCGTCAGCGTGCCCTTCCCCATCAGCATGTCC

ACCAACTGCAACAATGTCAACTAGTTCAATATATAATCCTTCCTACGTGCATGCATGGAC

GCGCTTGCGTGGAGCTTAATTTCTACGTTGATGGAGTGCTCATACGATGTTGAGCTACTA

AAAAATAAAATGAAGTTGCCTGTGTGTGTTATTTCAGTAGTGTGTCTATGTCTGTACCTT

GCACACACACTGTGTGTGCATATATATTTATGCGACATGTATGCACACGTCCGTATGCGT

GAGTGAACTGTCAACTCCTTGCTGTACTCCAGTCTAGCCGAGATGTGTACGTGTGATGTG

CCTGACCTACTTGTTCAGGCAATTAATGAATAGTAATTATTTTCCT

>Contig93

ACTCACTACCACTACTATTGCTAGCTTGATCGAGATGGCCCGTTCTGCTGTTGCTCAGGT

CGTGCTCGTCGCCGTGGTGGCCGCTATGCTCCTCGCAGTCACGGAGGCGGCTGTATCGTG

CGGTCAGGTGAGCTCTGCCTTGAGCCCCTGCATCTCCTATGCACGCGGCAACGGCGCCAG

CCCATCTGCGGCCTGCTGCAGCGGCGTTAGGAGTCTAGCCAGCTCAGCCCGGAGCACCGC

TGACAAGCAAGCGGCGTGCAAGTGCATCAAGAGCGCTGCTGCTGGGCTCAACGCTGGCAA

GGCCGCCGGCATCCCCACAAAGTGCGGCGTTAGCGTCCCCTACGCCATCAGCTCTTCGGT

CGACTGCTCTAAGATTCGCTGATCGAGCACTTGCTGCCATCGTTGTTGCTATCGTACGTC

CCCTACGCCATCGTTGCTGGATCTACGCTTAGTACGTTGAGGTCACACACACGCACACAC

ACATATATTTATGAATAAATGCTCTCATATTATCTCACTGCGTGAGAGAGAGACACACTG

TCCAAGCAGCTCTGCATGGCCGGCCACACTGTTGTATCGATGTTTGGTTGTTCCTCCACT

CCCCGAGGTTGCTGTACTTTGTACCATGTGTACTTTTGATATATGGATTGTATACACAGC

TGATCAGCTCAAAAAAAAA

>Contig113

CAGCTGCGGAGCGTCAGCGTGTGAGGCGCCAGCGCGCGCGGAGGAGGAGCTGGCTGGCTC

AGCGTCGCGCACGGCCGTGTGCTCCTACGTGGCACGGCGTGATGCTGAGTCAGCCAGTGG

AGTGGTGCTACTTTTTACTAGGTGTGGAGTGGGAGTGCTATGCGCGGGGTCGCGTGACGT

GGCAGTGGAGTGGTGCTACTTTTTACT

>Contig147

CACAGGTAGCTAGCAGTAACAATGGCGATGGCAAGGGTGCCCATCCTCATCGCGGTGGCT

CTCGTGGCCGCAGCGGCCTCCTGCAGCGCATGGGAGCCCACCATCCGTATGCCGACGGCG

GAGGCTGCGGCTGCTGCGGTCGACGACGCCGTGGCGCCGCTCATCCACGCACTGCGCCCG

CTGCTGGGCTCCAGCGGAGAGCTCGGCAGCCGCGGCGGCGTGCCGTGCGACAGCTGGAGG

CTGGGCGTGGAGGCGTACAACGTGCGTGACTGGAAGACTGTCCCCGCCAACTGCGAGGGC

TACGTGGGGCACTACATGCTCGGCAGCCACTTCCGGCGCGACTCCAAGGTCGTCATCGAC

CAGGCCATTGCCTACGTCGACAGCCTCAAGCTCGCCGGCAACGGCAAGGAGGTGTGGGTC

TTCGACATCGACGAGACCACGCTCTCCAACCTCCCTTACTACGCCACGCACGGCTTTGGG

GCTAGGCCATACAACGCGACGAGCTTCGACGCGTACGTGCTTGAGGGAACCGCGCCGGTG

CTGCCGGAGACGAAGCGGCTCTACTACAAGCTACTCAAGGTGGGCATCAAGCCGGTGTTC

ATCACAGGCCGGACGGAAGACAAGAGGGCCATCACCGTCGGAAACCTCCGCAGCCAGGGC

ATCTCCGGGTGGATGAACCTGACGCTCAAGCAGCCCGGGTTCCATGGCTCCGCGATATCC

TACAAGTCCGCCGAGAGGAAGAAGCTGCAGGACGCCGGGTACGTCATCGTCGGCAACATA

GGCGACCAGTGGAGCGATCTCCTCGGCGCGCCCGAGGGAGCTCGTACTTTCAAGCTGCCC

GACCCCCTGTACTACATCAGCTAAGCTTGCGGCCATTCATTGGACGAATGCTCGTGGTCC

ACGAATAAGCTCCATGCTCTTAATAATTATCTGCATGCTGGATGCATCAATAATAAGCTT

CTGTGTTGT

>Contig152

ATACATTACCATGGCCCCCGCCGTGATGGCTTCGTCGGCTACCACCGTCGCACCCTTCCA

GGGGCTCAAGTCCACAGCCGGTCTCCCCGTCAGCCGCCGCTCCAGCGGCAGCCTCGGCAG

CGTCAGCAATGGCGGAAGGATCAGGTGCATGCAGGTGTGGCCAATTGAGGGCATCAAGAA

GTTCGAGACCCTGTCTTACTTGCCACCCCTCTCCACGGAGGCCCTCCTGAAACAGGTCGA

CTACCTGATCCGCTCCAAGTGGGTGCCCTGCCTCGAGTTCAGCAAGGTTGGCTTCGTCTT

CCGTGAGCACAACAGCTCCCCCGGGTACTACGACGGCCGATACTGGACAATGTGGAAGCT

GCCTATGTTCGGGTGCACCGACGCCACGCAGGTGCTCAACGAGGTGGAGGAGGTCAAGAA

GGAGTACCCTGACGCCTATGTCCGTGTCATCGGCTTCGACAACCTGCGCCAGGTGCAGTG

CGTCAGCTTCATCGCCTTCAGGCCACCGGGTTGCGAGGAGTCCGGCAAGGCCTAAACTAC

GAGTTGAAACAATGGCCTATGTATGATGGTCTTTTGTGAACTTCACTTTTGTCCAAAACT

CTGCTTTCCGACCAGAGCATATTTTTCGTTCGGTATGATCTGTGTTTGTACGAACATAGG

TTGTGGAGCATGCATATGTAAATTCTAGCACCGGCTACACATATACAAC

>Contig156

ACACAGTCACACACAGACACACACCCCGATCCCTCACTCCCTCGCGTGTCTAGCAACCAA

GGGAAACAATGGCGCCGGTGAAGGTGTTCGGGCCGGCGATGTCGACCAACGTGGCCCGGG

TGCTGGTGTGCCTGGAGGAGGTCGGCGCCGAGTACGAGGTGGTCGACATCGATTTCAAGG

CCATGGAGCACAAGAGCCCCGAGCATCTCGTCAGAAACCCGTTCGGCCAAATCCCTGCCT

TCCAGGATGGGGATCTGCTTCTCTTCGAGTCACGCGCAATTGCGAGGTACGTGCTCCGCA

AGTACAAGAAGAACGAAGTGGACCTGCTGAGGGAAGGCGACCTCAAGGAGGCGGCGATGG

TGGACGTATGGACGGAGGTGGACGCGCACACCTACAACCCGGCCATCTCGCCGATCGTGT

ACGAGTGCCTCATCAACCCGCTCATGCGCGGCCTGCCGACCAACCAAACGGTGGTGGACG

AGAGCCTGGAGAAGCTCAAGAAGGTGCTGGAGGTCTACGAGGCGCGCCTGTCCAAGCACG

ACTACCTCGCCGGGGACTTCGTCAGCTTCGCGGACCTCAACCACTTCCCCTACACCTTCT

ACTTCATGGCCACGCCGCACGCGGCCCTCTTCGACTCGTACCCGCACGTCAAGGCCT

>Contig157

CTCTCTCCCCGTCCTCAAGATGCAGATCTTTGTGAAGACCCTCACCGGCAAGACCATCAC

CCTCGAGGTCGAGTCCTCGGACACCATCGACAACGTCAAGGCCAAGATCCAGGACAAGGA

GGGCATCCCTCCGGACCAGCAGCGTCTCATCTTCGCTGGCAAGCAGCTCGAGGATGGCCG

CACCCTGGCTGACTACAACATCCAGAAGGAGTCCACCCTCCACCTGGTGCTCAGGCTCCG

TGGTGGCATGCAGATCTTTGTCAAGACCCTCACCGGCAAGACCATCACCCTGGAGGTCGA

GTCCTCGGACACCATCGACAACGTCAAGGCCAAGATCCAGGACAAGGAGGGCATCCCCCC

GGACCAGCAGCGCCTCATCTTCGCTGGCAAGCAGCTCGAGGATGGCCGCACCCTTGCTGA

CTACAACATCCAGAAGGAGTCCACCCTCCACCTGGTGCTCAGGCTCCGTGGTGGCATGCA

GATATTTGTGAAGACCCTCACCGGCAAGACCATCACCCTGGAGGTCGAGTCCTCTGACAC

CATTGACAACGTCAAGGCCAAGATCCAGGACAAGGAGGGCATCCCACCGGACCAGCAGCG

CCTCATCTTTGCTGGCAAGCAGCTTGAGGATGGCCGCACCCTGGCGGACTACAACATCCA

GAAGGAGTCCACACTTCACTTGGTGCTCCGTCTCCGCGGTGGCCAGTAAGCTCCTGGCCA

TGGATCTGCTTCTGTCTCTGG

>Contig181

CCTTCGCCCTCGTCCTCCCCATTTCGCCAGCGGCGCAGCCCACCAACCACCCCCACCCGC

CGCCATGAGGGAGTGCATCTCGATCCACATCGGCCAGGCCGGCATCCAGGTCGGGAACGC

GTGCTGGGAGCTCTACTGCCTCGAGCATGGCATTCAGCCTGATGGCCAGATGCCCGGTGA

CAAGACCGTTGGGGGAGGTGATGATGCTTTCAACACCTTCTTCAGCGAGACTGGGGCTGG

GAAGCACGTCCCCCGTGCTGTCTTCGTAGATCTCGAGCCCACTGTGATTGATGAGGTGAG

GACTGGCGCTTACCGCCAGCTCTTCCACCCTGAGCAGCTTATCAGTGGCAAGGAGGATGC

AGCCAACAACTTCGCCCGTGGTCATTACACCATTGGCAAGGAGATTGTTGATCTGTGCCT

TGACCGTATCAGGAAGCTTTCAGACAACTGCACTGGTCTCCAGGGATTCCTTGTCTTCAA

CGCTGTTGGAGGTGGAACTGGCTCTGGCCTTGGTTCTCTTCTCCTGGAGCGCCTCTCTGT

TGACTATGGAAAGAAGTCCAAGCTTGGGTTCACAGTGTACCCATCACCCCAGGTCTCCAC

CTCTGTTGTTGAGCCATACAACAGTGTCCTGTCCACCCACTCACTCCTTGAGCACACTGA

TGTGTCTATCCTTCTTGACAATGAGGCCATCTATGACATCTGCCGCCGCTCCCTTGACAT

TGAGCGCCCAACATACACCAACCTCAACAGGCTTGTTTCTCAGGTCATTTCATCACTGAC

TGCTTCCCTGAGGTTTGATGGTGCTCTGAATGTTGATGTCAACGAGTTCCAGACCAACCT

GGTGCCCTACCCGAGGATCCACTTCATGCTTTCCTCCTATGCCCCAGTGATCTCAGCCGA

GAAGGCTTACCATGAGCAGCTGTCTGTTGCTGAGATCACCAACAGCGCCTTTGAGCCTTC

CTCCATGATGGCCAAGTGTGACCCCCGCCACGGCAAGTACATGGCCTGCTGCCTCATGTA

CCGTGGTGATGTTGTGCCCAAGGATGTCAACGCTGCTGTGGCCACCATCAAGACCAAGCG

CACTATCCAGTTTGTTGACTGGTGCCCCACTGGCTTCAAGTGTGGTATCAACTACCAGCC

ACCAGGTGTTGTCCCAGGCGGTGACCTTGCCAAGGTCCAGAGGGCTGTGTGCATGATCTC

CAACTCCACCAGTGTCGTCGAGGTCTTCTCCCGCATCGACCACAAGTTTGACCTGATGTA

CGCCAAGCGTGCCTTCGTCCACTGGTACGTGGGTGAGGGCATGGAGGAGGGAGAGTTCTC

TGAGGCCCGTGAGGATCTTGCTGCCCTGGAGAAGGACTATGAAGAAGTTGGTGCTGAGTT

CGACGAGGGTGAGGACGGTGACGAGGGCGACGAGTACTAGAGCCTGCCTCCTGGTGCTTT

CGCAAGGCGTGCTGCTGCTATCCCATGATCTGCCCGAGTGGCTTTATCTGTTATCTGTCT

GTTTGAATCTTTGCTTTGTGGTGTTTGTTTTACAACCTGTTGTGTTGTAAGACCCTTGTA

TCTTTGAACCTGTTATGCACCTTGGTTAATATGC

>Contig202

GCAAATCTAGCCATCTTATCATCTCCAGCTGAGCTCAGCATCACTAGTACTGCAAACTCG

ATCGGGATGGCTCGCGCTGCAGCTACTCAGCTCGTGCTGGTCGCCTTGGTGGCCGCTATG

CTCCTCGTAGCCACGGACGCGGCCATCTCCTGCGGTCAGGTGAGCTCTGCCTTGAGCCCC

TGCATCTCCTATGCCCGCGGCAATGGCGCCAACCCGACTGCGGCCTGCTGCAGCGGTGTC

AGGAGTCTGGCCGGCGCAGCCCGGAGCACCGCTGACAAGCAAGCGGCGTGCAAGTGCATC

AAGAGCGCTGCCGGTGGGCTCAACGCTGGCAAGGCCGCCGGCATCCCCTCCAAGTGCGGC

GTTAGCGTCCCATATGCCATCAGCGCTAACGTCGACTGCTCTAAGATTCGCTGATCAACC

ACTGCCCACCATGGCTGCCGCCCATAGCGCCATCCATCACCGCTGAGTATGCTGAGGTCT

CTAATACATATGAATAAATGCTCTGATCTGATCTCCATGTGAGGGAGAAAGGAGTGCGTA

CGCCGAGCTAGCTCTGCATGGCCGGCAACTATTGTACTACTACTATGGTTGTTTTTACTT

TCACTACCTTT

>Contig222

CTTCTCCCGTCCCGTCCCGTCCCCAGCCAACCTCCCATCTCCGTCTCGCGTCGCGGCGGC

AGTTCCACTCCCTCGCCATGGGCAAGATTAAGATCGGAATCAACGGTTTCGGAAGGATCG

GGAGGCTCGTCGCCAGGGTAGCCCTCCAGAGCGACGATGTCGAGCTCGTCGCCGTCAACG

ACCCCTTCATCACCACCGAGTACATGACCTACATGTTCAAGTACGACACCGTTCACGGCC

ACTGGAAGCACAGTGACATCAAGCTCAAGGACGACAAGACTCTGCTCTTTGGCGAGAAGC

CAGTTACCGTCTTCGGCGTCAGGAACCCTGAGGAGATCCCGTGGGGTGAGGCTGGTGCCG

ATTACGTTGTGGAGTCCACCGGTGTCTTCACTGACAAGGACAAGGCCGCTGCTCACTTGA

AGGGTGGTGCCAAGAAGGTGGTCATCTCTGCCCCAAGCAAAGATGCCCCTATGTTTGTGG

TTGGTGTCAATGAGGACAAGTACACCTCAGACGTTAACATTGTCTCAAATGCTAGCTGCA

CCACTAACTGCCTTGCTCCCCTAGCAAAGATCATTAATGACAACTTCGGTATTATTGAGG

GTTTGATGACCACTGTTCATGCCATCACTGCCACACAGAAGACCGTTGATGGTCCCTCGA

GCAAGGACTGGAGAGGTGGCAGGGCCGCAAGCTTTAACATCATTCCCAGCAGCACTGGTG

CTGCCAAGGCTGTTGGTAAGGTTCTTCCTGAGCTGAATGGCAAGCTTACCGGTATGTCTT

TCCGGGTTCCCACTGTGGATGTGTCAGTTGTTGATCTCACTGTTAGAACCGAGAAGGCTG

CATCATACGATGACATCAAGAAGGCTATAAAGGCTGCATCTGAGGGAAAACTCAAGGGGA

TCATGGGTTATGTCGAGGAAGATTTGGTCTCCACTGACTTTGTCGGTGACAGCAGGTCGA

GCATCTTTGATGCCAAGGCTGGAATTGCTCTGAACGACCATTTCGTCAAGCTCGTCTCGT

GGTACGACAACGAGTGGGGTTACAGCAACCGCGTTGTCGATCTGATCCGCCACATGGCCA

AGACTCAGTAGAGCGTTCTGATTTCCTTTGGTCAGGTGTCCCTGCTTTGTCTATGCGAAG

AATAAATGTGGATGGTGTCGCCAGACGACGCCTGGTTCATGTTAGTTGGACATGAGTTAC

CTTCTGTTTTCCGCATCAACTAGTACTTCTGTAATGGAGTTGCTAATCAAGTACTCTTTT

TTGGTTGTGGTTTGTGAAGATTATATAAATCGTTCAGTCCAGTACTTGTGTTTGCCTGAA

TAATCTTATTTCTCTTCCTTTC

>Contig243

GGCACGAGAACCCATGGCCCCCACTGTGATGGCTTCCTCGGCCACCTCCGTCGCACCCTT

CCAGGGTCTCAAGTCCACCGCGGGCCTGCCCGTCAGCCGCCGCTCCAGCAGCGCCGGCCT

CGGCAGCGTCAGCAACGGCGGAAGGATCAGGTGCATGCAGGTGTGGCCGATTGAGGGCAT

CAAGAAGTTCGAGACCCTGTCTTACTTGCCACCCCTCTCCACGGAGGCCCTCCTGAAGCA

GGTCGACTACTTGATCCGCTCCAAGTGGGTGCCCTGCCTCGAGTTCAGCAAGGTTGGCTT

CGTTTTCCGTGAGCACAACAGCTCCCCCGGGTACTACGATGGTCGATACTGGACAATGTG

GAAGCTGCCTATGTTCGGGTGCACCGACGCCACACAGGTGCTCAACGAGGTGGAGGAGGT

CAAGAAGGAGTACCCTGACGCCTATGTCCGCGTCATCGGCTTCGACAACCTGCGCCAGGT

GCAGTGCGTCAGCTTCATCGCCTTCAGGCCACCGGGTTGCGAGGAGTCTGGCAAGGCCTA

AACTACGAGTTGAAACAATGGCCTATGTATGAAGGTCTTTTGTGAACTTAACTTTTGTCC

ACCACTCTGCTGGACGACCAGAGCATATTTTTCATTCTATATGGTCTGTGTGTGTATGTA

CGAACA

>Contig247

GTTCACTTCCTGCGAGGTCCCCGAGCAATGGTTGCTCGGCGACGTCGTCGTGCCGGCAAA

GAGCGAGGACACGGGGGACCTGTGGCCCGCGGGGTCCCTGCTGTCCCCGGACTCCGAGCT

CTCGGAGCTCCCCGCGGCGAGCCTCCCUGGCUCGGCGUCACGCCGUGCCGGGCCGGCGGC

CAAGCGGCGTGGGAGGAAGCCCGGGCCACGCCCCGAGGGCCCCACTGTCAGCCACGTTGA

GGCCGAGCGGCAGCGCCGCGACAAACTGAACCGCCGGTTCTGCGACCTGCGCGCTGCAGT

CCCCACCGTGTCCCGCATGGACAAGGCCTCCCTCCTCGCCGACGCCGCCGCATACATCGC

CGAGCTGCGCGCCCGTGTGGCCCGGCTCGAGGACGAGAGCAAGCAGGCGGCCGCCGCGAG

GTGGGACACTAACTCGGCCTCCCTTGGCGGTGGCGGCGCCTCCTTCCAGAACTTTCTAGC

AGGTGACGAGACGGTGGAGGTGCGGATGGTGGGGCGAGACGCCGCCGCAGTGCGCGTGGC

CACGGCCGCGGGGTCCGCCCCGCACGCGCCGGCGCGGCTGATGAGCGCGCTCCGGTCGCT

GG

>Contig330

TTCGGCACGAGCTCCTCCTAGCAAGCTATATACCTACATAGTACAGCCATGGCCCCCACC

GTGATGGCCTCGTCGGCCACCTCCGTCGCTCCTTTCCAGGGGCTCAAGTCCACCGCCGGC

CTCCCCGTCAGCCGCCGCTCCAACGGCGCTAGCCTCGGCAGCGTCAGCAACGGTGGAAGG

ATCAGGTGCATGCAGGTGTGGCCCATCGAGGGCATCAAGAAGTTCGAGACCCTGTCCTAC

CTGCCACCGCTCAGCACGGAGGCCCTCCTCAAGCAGGTCGACTACCTGATCCGCTCCAAG

TGGGTGCCTTGCCTCGAGTTCAGCAAGGTTGGGTTCATCTTCCGTGAGCACAACGCATCC

CCTGGGTACTACGATGGCCGGTACTGGACAATGTGGAAGCTGCCTATGTTCGGGTGCACC

GACGCCACACAGGTGATCAACGAGGTGGAGGAGGTCAAGAAGGAGTACCCTGACGCGTAT

GTCCGCATCATCGGATTCGACAACATGCGCCAGGTGCAGTGCGTCAGCTTCATCGCCTTC

AAGCCACCGGGCTGCGAGGAGTCCGGCAAGGCCTAAACAGCTCACTCACCACGGGCCACA

TATAAAGTGCCATTGCAGTTTTGTCAACTCTGACATTGCTTTGGGTTTTCCTTCTCCATT

TATCTTTCTTATTTGTTCCTAAGAATATGTGTATGTCCATGTTCATGTACCAACATGGCT

CGAGAAAGCATGCTCGTATGTGAATGCTATCGGTGGCTATGTATATGCACGGACGCATAA

TATTATACAATATGTCAACTATATACGTTTTAATGGTACCC

>Contig354

GGCACGAGGCTCACACTTAAAACAACCACCAGCAGTAGCTCGGCTCCGGAGAGGCAGAGT

CAGAGGCAAACATGGGCTCCACCGCAGCCGACATGGCCGCCTCCGCCGACGAGGAGGCGT

GCATGTATGCTCTCCAGCTCGTCTCGTCGTCGATCCTCCCGATGACGCTCAAGAACGCCA

TCGAGCTGGGCCTCCTGGAGACCCTGGTGGCCGCCGGCGGCAAGCTGCTGACCCCCGCCG

AGGTGGCAGCCAAGCTCCCGTCCACGGCGAACCCCGCCGCGGCGGACATGGTGGACCGCA

TGCTCCGGCTGCTGGCCTCGTACAACGTGGTGTCGTGCACGATGGAGGAGGGCAAGGACG

GCCGCCTGTCCCGGCGGTACGGCGCCGCGCCCGTGTGCAAGTTCCTCACCCCCAACGAAG

ACGGCGTCTCCATGGCGGCGCTCGCGCTCATGAACCAGGACAAGGTCCTCATGGAGAGCT

GGTACTACCTGAAGGACGCGGTCCTTGACGGCGGCATCCCGTTCAACAAGGCGTACGGGA

TGTCGGCGTTCGAGTACCACGGCACGGACCCGCGCTTCAACCGCGTCTTCAACGAGGGGA

TGAAGAACCACTCCATCATCATCACCAAGAAGCTCCTCGAGGTCTACAAGGGCTTCGAGG

GCCTCGGCACCATCGT

>Contig356

CCCTCCGAATTTCCCGGCCAGAATGGGGGAGTCCAGCGCGCTTCAGTCCATCCTCTACGA

CCGCGGCGCGCTTCGCCTCCTCGATCAGAGGAAGCTGCCCCTCGAGGAGGTCTACATCGA

TGTCAAGGACTCCGCGGATGGATGGAATGCGATCAGAGACATGGTTGTTCGTGGTGCTCC

TGCCATAGCCATAGCGGCAGCACTATCATTGGCTGTGGAAGTTAATGATCACGATTTCAC

TGGTACACCTGTGGAAGCAGCCTCATTCGTTTCCAAGAAGTTGGAATACCTTGTATCCAG

CCGGCCCACAGCAGTGAACCTGTCTGATGCTGCTACAAAGCTTCAGAACTTAGTGTCAAG

AACAGCTGAAACAGCAAAAGATGCCAAATCTATCTTTCAGGTCTTTATTGAAGCTGCAGA

GGCTATGCTAGTTGATGATGTGGCCGATAATAAGGCAATTGGCTTGCATGGAGCTGAATT

CCTTCAACGCCAGCTTGGAAGTTCGAGAAACATCTCTGTTCTAACCCATTGTAATACTGG

CAGCCTTGCAACTGCTGGTTACGGAACTGCCCTAGGGGTCATTCGTGCTCTTCACTCTGG

AGGAGTTTTGGAGAAGGCCTTCTGCACTGAAACTCGTCCATTTAACCAGGGTTCCAGGCT

TACAGCCTTCGAGTTAGTTCATGACAAAATACCTGCGACGCTGATAGCAGACTCTGCTGC

AGCTGCACTTATGAATAATGGACAAGTTCAAGCTGTAATTGTGGGTGCTGATCGTATAGC

TGCAAATGGTGACACAGCCAACAAGATTGGCACATACAACCTCTCGATTTCTGCAAAGCA

TCACGGCGTGCAGTTTTACGTGGCCGCACCAGTAACTTCGATTGATCTCTCCCTCCCGTC

TGGGAAGCAAATCGTCATAGAAGAAAGATCTCCAAAGGAATTGTTGAACTCTGAAGGTGG

TCTAGGAAAGCAAGTTGCCGCGTCAGGTATATCAGTCTGGAACCCCGCCTTCGACGTTAC

TCCAGCAAATCTAATTACTGCAATCATTACAGAAAAGGGTGTGATCACAAAGTCTGGTCC

TAATGGAAGTTTCGACATCAAAGGTTTCATCGAGCGTGCCAAGTGATGCCCTCCAGTGAG

CATAGGTTGGCATGAATGCAGTGGAACATCCTTCTCACACAATTGATGATAGGATGCCCA

TGTTGTACTCCCAGCAATAAGATCTTTCGAAGTGAGGGAAAAATAAAGTCGCGTAC

>Contig362

GCACGAGGCTCCTCCTCCCCGTCCGTCCCCACACACACACGCACGCGTTTGACATTTTCC

GCCCACCACCGCCGGAGGGAGCTCCAGCCATGGCCGCCAAGTGCTACCCGACGGTCAGCG

ACGAGTACCTGGCCGCCGTCGCCAAGGCCAAGCGCAAGCTCCGCGGCTTCATCGCCGAGA

AGAACTGCGCGCCCCTTATGCTCCGCCTCGCGTGGCACTCGGCCGGGACCTTCGACGTGG

CCACCAAGACCGGCGGGCCCTTCGGCACCATGAAGTGCCCCGCGGAGCTCGCGCACGGCG

CCAACGCCGGCCTCGACATCGCCGTCAGGCTGCTCGAGCCCATCAAGGAGCAGTTCCCCA

TCCTCTCCTACGCCGACTTCTACCAGCTCGCTGGAGTCGTCGCCGTCGAGGTCACCGGCG

GGCCCGAGGTTCCCTTCCACCCGGGGAGACAGGACAAGCCCGAGCCTCCTCCAGAAGGCC

GTCTTCCTGATGCTACCCAAGGCTCTGACCACCTCAGGCAGGTGTTTTCCACTCAGATGG

GTTTGAGTGACCAGGACATTGTTGCTCTTTCTGGTGGTCACACCCTGGGAAGATGCCACA

AGGAGAGATCCGGCTTTGAGGGAGCCTGGACCGCCAACCCTTTGATCTTCGACAACTCTT

ACTTCACTGAGCT

>Contig363

AAGACAAGAGAAACAATGGCTCCCATGCTCAAGCGGATCGCCCAGGACGAGCCCAAGAAG

GCGGCGTACGTGACCTTCCTCGCCGGCTCCGGCGACTACTGGAAGGGCGTGGTGGGCCTG

GCCAAGGGCCTCCGCGCCGTCAAGTCGGCCTACCCGCTCGTGGTGGCCGTGCTCCCCGAC

GTCCCCGAGGACCACCGCCAGAAGCTGCTCGCCCAGGGCTGCCTCGTCCGCGAGATCGTG

CCCGTCTACCCGCCGGAGAGCCAGACCCAGTTCGCCATGGCATACTACGTCATCAACTAC

TCCAAGCTCCGCATCTGGGAGTTCGTGGAGTACGAGAGGATGGTGTACCTGGACGCGGAC

ATCCAGGTGTACGACAACGTCGACCACCTCTTCGACCTCGAGAAGGGCAGCTTCTACGCC

GTCAAGGACTGCTTCTGCGAGAAGACGTGGAGCCACACCAAGCAGTACGAGATCGGCTAC

TGCCAGCAGTGCCCCGACAGGGTCGTCTGGCCGGAGCGCGACCTCGGCGTGCCCCCGCCG

CCGCTCTACTTCAACGCCGGCATGTTCGTGCACGAGCCCAGCATGGCCACCGCCAAGGCC

CTGCTCGACAAGCTCGTCGTCACCGACCCCACCCCCTTCGCCGAGCAGGACTTCCTCAAC

ATGTTCTTCAGGGACGTGTACACGCCCATTCCGCCCGTCTACAACCTCGTCTTGGTCATG

CTCTGGAG

>Contig367

CGGCACGAGGCTCGCTCTCCGCTCCATCTGGTCCGATTCGATTCGATTCGATCTCGGCTT

CCTCTCCAAGCCACGCGAGACCAGCCATGGCCGCCACGATCCAGTCCGTGAAGGCCCGCC

AGATCTTCGACAGCCGCGGCAACCCCACCGTCGAGGTTGATGTGTGCTGCTCAGATGGAA

CCTTCGCCAGGGCCGCCGTTCCCAGCGGTGCATCAACTGGTGTCTATGAAGCTTTGGAGC

TGAGGGACGGTGGATCTGACTACTTGGGCAAGGGTGTTTCCAAGGCTGTTAACAATGTGA

ACTCCATTATTGCACCCGCTTTGGTCGGCAAGGACCCTACCGCTCAAACTGAGCTCGACA

ACTTTATGGTTCAGCAGCTTGATGGAACCAAGAATGAGTGGGGTTGGTGCAAGCAAAAGC

TTGGTGCTAATGCAATCCTGGCTGTATCACTAGCTGTTTGCAAAGCTGGAGCCAGCGTCA

AGAAGATTCCACTGTACCAGCACATTGCCAACCTTGCTGGCAACAAGCAATTGGTTTTGC

CCGTTCCTGCGTTCAATGTCATCAATGGTGGATCCCATGCTGGAAACAAGCTTGCTATGC

AGGAGTTCATGATCCTTCCTACTGGAGCTACCTCATTCAAGGAGGCAATGA

>Contig370

CTGTCTCAGTTTACAAGTTTCATCACTGAAGCTTTACTTTGTGTAATGCTACACATTAAC

CTTGTTTTCTTATTAGTTTTTATTGCACCATGGTATAACCTTATATCGAGTACTAATTAT

CATTCTCTAAGTCCTTTCATCTCTAGGTGAAAAATATTGGATTCACAGGCACACTACGGC

TACTCTTTAAGCCTCTGGTAGCCGAACTCCCATGCTTTGGAGCTGTTTGCGTTTCTTTGA

GAGAGAAGAGCAAGGTGGATTTTACCCTCAAAGTTGTTGGTGGCGAAATGACAGCAATTC

CTGGAATTTCTGATGCAATTGAGGGAACAATACGTGATACCATCGAGGACACACTGACAT

GGCCTAATCGCATAATTGTCCCCATTGTGCCAGGAGATTATAGTGATCTGGAGCTAAAAC

CTGTTGGATTATTAGAAGTAAAACTTGTGGAAGCTAGGGATTTGAAGAACAAGGACCTCG

TTGGGAAGTCTGACCCTTTTGCTGTGCTATACATACGTCCACTGAGTGCAAAAACGAAGA

AAAGCAAAACAATAAACAATGATTTGAACCCCATCTGGAATGAACACTATGAATTTGTGG

TGGAGGACTCATCTACCCAGCACCTGACTGTGAAAATTTACGACGACGAAGGGCTCCAGC

CGTCAGAGATTATTGGCTGCGCTCGAGTAGACTTATCGGATGTTACGCCTGGAAAGGTCA

AGGATGTTTGGTTGGAACTTGTGAAAGACCTGGAAATTCAGCGTGATAAGAAACCTCGTG

GTCAGGTCCACCTAGAGCTCCTGTACTACCCTTTTGAGAAACAAGAAGGGGTTTCCAATC

CTTTTGCTGGTCAGATCCAGTTAACTTCTTTGGAAAAGGTCCTCAAGACGGAATCTAATG

GATATGATGTCAACCAGAGGAAAAATGTTATTACGAGAGGAGTCCTTTCAGTAACTGTTA

TATCTGCAGAGGACATACCAGCAATGGATGTTGATGGGGAAGGCTGACCCATTTGTTGTC

CTGTACCTGA

>Contig371

ATTGGGCTCGCAATTTTGGTTCCTTGCCTTAGCCTCTTCCTGCACATAGAGAGAGCATCG

ATAGCAAGCTGATTAGGCTAGGTAGATAGATTAGCTAGCCTCTGCTGCGGCTCTGACCAG

AGAACTATACAAGCTTGATTAGGAGGAGATCGAAGCCAAGATGATGCGCGGGAAGGACCA

GAGGCCCGCGGCATTCTCCCCGATGAGGGAGTCGGTGGCGGCGGCGGTGCAGGAGGAGGT

GTGGGAGGTCCGGCCCAGCGGCATGCTGGTGCAGAAGCGCACCCCGGACTCGGATCCCCA

CCATCCGCGTCAAGGTCAAGTACGCCGGCGTGTACCACGAGGTGTACATCAACTCCCAGG

CCTCCTTCGGGGAGCTCAAGAAGCTCATGTCCGAGAAGACGGGGCTGCACCCGGACGACC

AGAAGGTGGTGTACAAGGACAGGGAGCGCGACTCCAAGGCCTTCCTCGACATGGTCGGCG

TCAAGGACCGCTCCAAGATGACGCTGCTCGAGGACCCCACCGCCCAGGCCAAGCGCCTCA

TCGAGGAGCGCCGGAATGCCAAGGCCCAGCGCGCCGCCAAGGCCGTCTCGCGCGTCAGCC

TCGACGTCGACAAGCTCGCGTCCAAGGTGTCGGCGCTGGAGACGATCGTTAGCAAGGGCG

GCAAGGTGGTGGAGGCCGACCTGGTCACGCTCACCGAGGCGCTGATGAGCGAGCTGCTCA

AGCTAGACGCCATCGTCGCCGAGGGCGACGTCAAGGACCAGCGGCGGATC

>Contig373

GGCACGAGGCTTGGCATACAAGAACGACAGATCAGTCGGTCAGTCAGTCCAGTCCCCTGT

TCCTGTGCGAGTGGCGAGTGAAGTGACGGGCGTGGTTTCTTGGGAGTTTGGAGCCGGAGG

AAGAAGAAGATGGCAGCCGCGCCGCCGCCCAAGGCCGACGAGCTGCAGCCGCATCCGCCC

AAGGAGCAGCTGCCCAGCGTCTCCTTCTGTATCACCAGTCCGCCGCCATGGCCCGAGGCC

GTGATTCTAGGATTTCAGCATTTCATAGTCATGCTGGGCACCACCGTCATCATACCAAGC

GCGCTTGTTCCTCAAATGGGAGGTGGAAATGAGGAGAAAGCTCGGGTAATCCAGACGCTC

TTGTTCGTCGCCGGCATAAACACCTTACTGCAAACCTTCTTCGGGTCTCGCCTCCCCGTC

GTGATGGGCGGCTCCTACACCTTCGTCGCGCCGACCATCTCCATCATCTTGGCTGGACGC

TATGACAATGAGACAGACCCTCACGAGAAATTCCTACGGACGATGAGGGGGACACAAGGC

GCTCTCATAATCGCCTCCACGATCCAGATCATCCTTGGCTTCAGCGGCCTCTGGCGCAAT

GTTGTCAAATTGCTGAGCCCATTATCTGCAGTTCCTCTTGTTTCACTTGGTCGGATTTGG

GCTTTATGAACTCGGCTTCCCAGCGGTAGCCAAGTGCGTGGAAATTGGTCTGCCAGAACT

CATCCTGATGGTCGCATTTTCTCAGTATTTGCCTCATGTGCTGCATTCTGGGAAGGGTGT

CT

>Contig374

CACTATGATTGATGGTTTGGGTGTTGCTGGCTGGGGAGTTGGTGGTATTGAAGCAGAGGC

TACAATGCTTGGGCAGCCGATGAGCATGGTGTTGCCTGGTGTGGTTGGGTTCAAGTTGAC

TGGAAAGCTAAGGAATGGTGTCACTGCTACCGACCTTGTTCTGACTGTTACCCAAATGCT

AAGGAAGCATGGTGTTGTTGGCAAATTTGTCGAATTCCATGGTGAAGGTATGGGCAAACT

GTCTTTGGCTGACAGGGCCACAATTGCTAACATGTCACCAGAATATGGAGCTACCATGGG

CTTCTTCCCTGTAGACCATGTGACATTAGATTATCTCAGATTGACTGGCCGAAGCGATGA

AACTGTGTCAATGATCGAAGCATATCTACGAGCTAATAATATGTTTGTGGACTATAATGA

GCCTCAATTAGAAAGAGTTTACTCTTCGTATCTTGCGCTGGACCTTGATGAGGTAGAGCC

TTGCATTTCTGGCCCGAAGAGGCCGCATGACCGTGTCACTTTGAAGGACATGAAATCAGA

TTGGCATGCTTGCCTGGACAACAAAGTTGGTTTCAAGGGCTTTGCGGTGCCGAAGGAACA

ACAGGATAAGGTTGTGAAATTTGACTTTAATGGACAACCTGCTGAACTGAAGCATGGCAG

TGTTGTTATAGCAGCAATTACTAGTTGCACAAACACATCAAACCCCAGTGTTATGCTTGG

TGCTGCCCTTGTCGCAAAGAAAGCTTGCGAATTGGGTCTCGAGGTCAAACCATGGGTAAA

GACAAGCCTTGCCCCTGGATCTGGGGTTGTCACTAAATACTTGCTAAAGAGTGGCCTTCA

AGAATATTTCAACAAGCAAGGATTCCATCTTGTTGGATATGGGTGTACCACTTGCATTGG

CAATTCTGGTGAACTCCATGAATCTGTGTCAGCTGCCATCACAGAAAATGATGTTGTTGC

TGCTGCTGTGTTGTCGGGCAACCGTAACTTTGAGGGGCGTGTGCACCCTTTGACTCGGGC

CAATTACCTTGCTTCACCACCTCTGGTTGTTGCATATGCACTTGCTGGCACTGTTGACAT

TGATTTTGAG

>Contig375

ACGAGGTCTCGTCTTATCCGATCTTCCTGGCACAACGTTCTACCGTCGCGAATCGATCAT

GGAGCTACCGACCAAAGACCAAAGGTACAATCGATAATCTACAGCTGAAGCATACATAAA

CTTAAGACAAATTACTGCTCTCGTAAATTGTATTCTGTTCTGACATCGTTTACACAGGAA

TTCGTAATCACTGGAACCTATTTAAGTTACTCTCATAGTTGTGGTAAAATTATTCGTGTG

ATTCGCTGTTGCATTCGTGTGATTCGCTGTTGCATATAGTACCACATCACAGCTCAAATT

TGCACTTCAATAACAATCGGCGGTCCAAACGACCAAATCGCTTTGGTATCAGATTCAATA

TATTTTATTTTTTCCTCAGAATATGGTATATGTGTCAGGTTGTTATTGTGTCTAAATTTA

TGTTAATATATTTGATGATCACAATTTGATCTTGGTCTCCTATTTCAATTTGTAAACTCT

TACCTAATGTAGCCTGAACAGCTGTATGATCGGATTGTATAGTACACAAATGAAATCTAC

AGTTATCTTCCCTGGACCTGGACATCATATTTGTTCTAATCGCCATTGGCTGAACCACTA

TGAAGAAGCCTGCCTTGATTTTGGAATATGTGCTCAGCAGAAGAATCTAACCTGGGACGT

GGACCTCAATTCCGGTCATTGTAGGCCCAGCAGACTTTCAGAAACTGGTTAAGATGTGCT

CTTCATCAGTAAATTATGTGATATTTGATTCTTCTGCA

>Contig377

TCTAGGGTTTCCCAACCACCCCCGCAACCGCAAACCCCCACCCAGGCACGACTCGATCCG

AAAACCACCCCACCACCACCGGCGATGTCGACGGCGGCGGCGCAGGGCGGCGAGAAGCCG

GCGCTCAGGAAGCCGGTGTTCGTCAAGGTGGACCAGCTCAAGCCGGGCACCAACGGCCAC

ACCCTCATCGTCAAGGTCGTCAGCGCCAACCCCGTCCCTGGCCGCGTGCGCACTGGTGCG

CCCGCCTCCTCCTCCGCCCGCGCCCCCCGCATCGCGGAGTGCCTCGTGGGCGACGAGACC

GGCTCCATCATCTTTACTGCCCGCAACGACCAAGTTGACCTGTTGAAGGCTGGTGCCACA

GCCATATTGCGCAACGCTAAAATTGACATGTTCAAAGCTTCGATGAGGCTTGCGGTGGAC

AAGTGGGGGCGGGTAGAAGCTACAGAACCTGCCAGCTTTACTGTGAATGAAGAGAACAAC

TTGTCGCAAGTAGAGTACGAGCTGGTTAACGTGGCCGAGTAATTGTTGAAGTTTCCCTGC

CCTTTTCCCGCCATAAAAACGAAGCACTATGAATGAATATTATCAAGCCGTTGGTAAGCT

TGTTTATTTCTTATGGTGGTAGTAGATAAGATCAAGATTGTATCACTTGAGCTAGAATGT

TCGATGGATCAAACTGAGCTTTCCATGCATCTTACTGGATTGGGATTCCTCTTGGCACTG

CTTGTATTGCT

>Contig380

GGCCGCAAGCCGTCCGCCTCGCTCTTCTCCCGAGCCTGGTCCTCATCCTCTCGCCTCTCT

CCCCCCACACAGATTTAGCAATCTAGAGTTAGCGCCTCCGTCCATCTCGTAGCTGCTCCG

ATGGCGATCAAGAGGACCAAGGCCGAGAAGAAGATCGCGTACGACCAGAAGCTCTGCCAG

CTGCTCGAGGAGTACACCAAGGTGCTCATCGCCGTCGCCGACAATGTCGGCTCCAAGCAG

CTCCAGGAGATCCGCAAGGGTCTCCGCGGTGACTCCATCGTGCTCATGGGCAAGAACACC

CTCATCCGCCGCTGCATCAAGGTGCACTCGGAGAAGACCGGCAACAAGGACTTCCTTGAG

CTCAGCAACCTCCTCGTCGGTAACGTTGGCCTCATCTTCACCAAGGGTGACCTCAAGGAG

GTTCGCGAGGAGGTCGCCAAGTACAAGGTTGGTGCTCCTGCTCGTGTTGGGCTAGTTGCA

CCTGTTGATGTGGTGGTTCCCCCTGGCAACACTGGTCTGGATCCCTCCCAGACGTCCTTC

TTCCAGGTGCTTAACATTCCCACCAAGATTAACAAGGGTACTGTGGAAATTACTATCCCA

GTGGAGCTAATCAAGAAAGGTGACAAGGTGGGCTCCTCTGAGTCTGCCCTGCTTGCCAAG

CTTGGTATCCGCCCCTTCTCATATGGACTGGTCATCTGCAATGTCTATGACA

>Contig382

GCACGAGGCTCATCTCTTCTTCCTCCTGCCGGCCTCCCTCTCGGCTGGCTGGCTCTTTGC

CACGAGCGATCACCACGAAGAAGAAGGCAGAAGGCAGAACCTAACCTCGGAGATGGCGAC

AATCATCATGAGAGTCGACCTTGACTGCGACCGATGCTACAAGAAGATCAGAAAGGTCCT

CTGCAAGCTCCAAGACAGAGAAAACATCAAGATGATCTCGTACGACGAGAAGAGCAACAC

GGTGACTGTGTCCGGCTCCTTCGACGCCGAGGAGGTCGCCGACAGGCTCTGCTCCGATGC

TGGCAAGGTGATCACCGACGTACAAGTCGCCAGGGGGAACCAGATGAACCCCAGTGCCAA

GGTGGCGCCCAAGCAGCACGGCAAGGAGGGCCACGGCCCCGGCCCCGGCCCCCAAGCGCA

CGGTCACGGCCACGGCGCTGGGCCCCAAGCGCACAGTTACGGCGGCAAGCCGGAGAAGAC

CAAGCACGTCAAGTTCGGCATGGATGACGACGAACGAATCGATCGGCACGGCCCCGGACC

GCAAGTGCACGGTCACGGCGGCAAGCCGGACAAGACCAAGCACGTCCAGTTCGGCATGGA

GGACGACGAACGAATCGATCGGCGCGGTCACCACGACCAAGGCCACGGACAAGGACATGG

ACATGGACACGGCAATGGCCACGGCGGAAAGCCGAAGGTGGTAACTACCACGGCGATGTC

ACGGAATGAGGCCCCGCGCGGCCAGCAGCCGGCGTCCATGGCGGCCATGGCGCCGATGCG

GATGCCGACGCCGGCGCCTAGCATGACGATGATGCCGCAGGCGATGGCCACGCCGTCCAT

CTGGCCGGCCGCCCCCTGGGTGGGGGTACAGCGCACCCGCATA

>Contig383

GGCCTCACGTGCCGCTCGACGACGCCCAGAAGATCACCGACGACACCCGCATCCGCGCAT

CCATCCCCACCATCAAGTACCTCCTCGAGAAGGGTGCCAAGGTCATCCTGGCCAGCCATC

TGGGCCGCCCAAAAGGTGTCACCCCCAAGTTCAGCTTGAAGCCTCTTGTTCCACGCCTGT

CTGAGCTCCTTGCACTTGAAGTTGTGATGGCCCCTGACTGCATCGGTGAAGAAGTTGAGA

AATTGGCTGCTGCTTTGCCAGATGGCGGTGTTCTACTCCTAGAGAATGTGAGATTCTACA

AGGAGGAAGAGAAGAACGATCCTGAGTTTGCTAAGAAGCTTGCATCAGTTGCTGACCTTT

ACGTAAATGACGCTTTCGGCACTGCACATAGGGCTCATGCTTCAACCGAGGGTGTAACCA

AGTTTTTGAGGCCTTCTGTTGCTGGCTTCCTCATGCAGAAGGAACTTGACTATCTTGTCG

GAGCTGTTGCCAACCCAAAGAAGCCATTTGCTGCCATTGTTGGTGGATCAAAGGTCTCAT

CTAAGATTGGTGTGATCGAGTCTCTGCTGGCCAAGGTTGATATCCTCATCCTTGGTGGTG

GTATGATCTTCACATTCTACAAGGCCCAGGGATTACCTGTTGGAAAGTCCCTTGTGGAGG

AAGACAAACTTGAACTGGCAACTTCACTGATTGAAACGGCAAAGTCCAAGGGTGTTAAGC

TCTTGCTTCCGACTGATGTCGTTGTGGCTGACAAGTTTGCAGCAGATGCCGAAAGCAAGA

TTGTTCCTGCCACTGCTATCCCTGATGGTTGGATGGGTCTGGATGTTGGCCCAGATTCCA

TCAAGACTTTTGCAGAAGCCTTGGACACCACCAAGACTGTTATCTGGAACGGTCCTATGG

GAGTCTTT

>Contig385

CGCTGGGTGGGTGCGCAGATGGGCAGCGTGGACGCGGGCGCGGCGATGGAGAAGATCCGG

GCGGCGGGGCTGCTCAGGACGCGCGGCCTCATCGGCGGCAAATGGGTCGACGCCTACGAC

GGGAAGACCATCGAGGTGCAAAATCCAGCAACTGGCGAGGTCCTAGCAAATGTAGCCTGC

ATGGGCAACAGAGAAACAGCTGATGCAATAACTTCTGCTAACACTACATTTTATACTTGG

AGCAAACTCACTGCAAGTGAAAGGAGCAAGGCACTAAGAAAATGGCATGACCTACTTATG

TCACACAAGGAAGAACTTGCACTTCTTATGACACTGGAACAGGGGAAGCCTATGAAAGAA

GCCCTTGGGGAGGTGAACTATGGTGCAAGTTTCATAGAATTTTTTGCTGAGGAAGCAAAG

CGTGTGTATGGTGATATTATTCCCCCAACTCTAGCTGACCGCAGACTATTGGTTCTGAAG

CAGCCTATTGGCGTAGTTGGAGCTATTACACCATGGAATTTTCCTTTAGCAATGATAACC

AGAAAGGTTGGACCAGCATTGGCTTGTGGATGCACTGTTGTTGTCAAGCCATCAGAGTTC

ACACCTCTGACAGCATTAGCTGCAGCAGACCTTGCTCTTCAAGCTGGAATACCAGCTGGT

GCACTAAATGTTGTGATGGGTAATGCTCCTGAGATAGGTGATGAACTAATGCAGAGCATG

CAGGTCAGAAAGATTACGTTCACGGGTTCAACAGCTGTTGGCAAAAAACTGATGGCTGGA

TCAGCAAACACTGTGAAAAAGGTTTCTTTGGAGCTCGGTGGGAATGCGCCTTGCATTGTT

TTCGATGATGCAGATATTGATGTTGCCGTTAAAGGCAGTCTTGCTGCCAAGTTTCGGAAC

AGTGGACAGACGTGTGTATGTGCAAACAGGATATTGGTGCAAGAAGGTATCTATGAAAAA

TTCGCAAGTGCATTTGTCAAGGCTGTTCAGAGTTTGCAAGTCGGTAATGGGCTAGAAGAG

AGTACATCACAGGGTCCTCTAATCAATGAAGCTGCTGT

>Contig386

GGTAAAGTAAGTTATTCCATGACCGACAAGATATCTATATATATATTTCTACAAGGCAAG

GGAACCAACCTTATTTCTTCTACTTACTCCATAAGGGAGATGGATATGATTAGCTTTCTA

ACGCGAACAATATAATTAATTATAAGCTACTGATGGATCCGACTCACTCACTCCCTCCCT

ATACACATCAGTAGTAGTCTTCCTCTTCTTCTCTCTGCTCTCTGTGGGGGATAAATATAT

AGATGGATGGTGGTGTATATATGCTACTGCTACTGGCTCTGGGGTCTATGAATCAGTGGC

CGTTGTCGGTGGATCCGTAGCCGTAGGGGTTCTTGCTGGCCCAGTTCCACAGGTCTCTGC

ACATCTCCTCCACCCCATACTTGGCCTTCCACTTGAGCTCCTTCTCAGCTTTGGCGGTGG

CAGCGTACACAGTCTCGGCATCTCCAGCTCTTCTTCCAGCATAAACCAGAGGGATTTTCT

TTCCGCAAGCCTTCTCGAATGCAGCAACCATCTCCAGCACGGACGTCCCCTTTCCAGTGC

CCAGATTGTACACTTCGCACCCTATTCTGTCAGAATCTTCGTAGAGCTTCCTCAAGGCGG

CGATGTGGCCGTCAGCCAGGTCAACAACATGGATGTAGTCACGTACCCCTGTTCCATCCT

TGCTGTTGTAGTCGGTCCCATAAACCGTCAGCGCAGGCCGCCTCCCGACAGCGACTTGCT

GGACATAGGGCATCAGGTTGTTGGGGACTCCAAGGGGGTCTTCCCCGATGTAGCCGCTCG

GGTGAGCGCCGACTGGGTTGAAGTACCTCAGCAGTATGATCTTCCAGTCAGGGTCCGAGC

GCTGGAGGTCACGGCAGATGTCTTCAATGACAAGCTTTGTTCTGCCGTAAGGGTTGGTCG

CCGAGAGCGGGAACTCCTCGGTGCAGGGCACCTCCTTGGGCCACCCGTACACAGTGGCTG

ATGATGAGAAGACAAGCTTCTTGCATCCATGTGCTGCCATGACTTGGAGG

>Contig387

GCACGAGGGTTCCATTTCCTACGTTTCTTCCTCGAGTCAAGTGGCTCTTGATTGATCCCA

CCACCAGCCGCACCACCAACGTCTTCTGTCCGTAGCTCCTTGGTGCCTAGCTGCTGTGTT

CACATCACATCTCCATCGATGAAGACGAAGCAGGCTTCCAAGGCCAAGGCGGCGCCGTCG

CCGGGGAAGGAGGAGGAAGCAGTTGCATCGGGTGGTTTCCGCAAGGGGCCATGGACGGAG

CAGGAGGACATGAAGCTGGCGTGGTTCGTGCGGCTCTTCGGCGAGCGCCGCTGGGATTTC

TTAGCTAAGGTGTCAGGTTTGCAAGGTGGCGGGTGACCCATGCCGTGCCATGCATGCGCC

ATGCATGCATGCATGTCTGGGACCAAAATATGATGTAGCCATGGTCCTTTGTGTGCTAAC

GCTCTCGGTCTCCGCCGGCACCTCTTGCTCTTGTGTTTGTCTTGGCGACAGGTCTTAACC

GGACGGGGAAGAGCTGCCGGCTCCGGTGGGTCAACTACCTGCACCCGGACCTCAAGCGCG

GCCGGATGAGCCCCGAAGAGGAGCGCCTCGTCGTCGACCTCCACGCCCGCTGGGGCAACC

GCTGGTCACGCATCGCCAAGGCCATGCCGGGGCGCACCGACAACGAGATCAAGAACTACT

GGCGCACCCACACCCGCAAGCTCCACAAGGACACGCGCGCCTCTGCTGCTTCGGCCTCTA

CGACCACGTCCACGTCCATGTCGGCGGCGTCTCCGGCCACCACGTCCAGCTCCTCCTCTT

CAACGATCGACAACGAC

>Contig388

AGAAGGAGCACGGCGTCAAGAGCGTCTACGTCTGGCACGCCATGGCCGGCTACTGGGGCG

GCGTCAAGCCGTCGGCGGCCGGGATGGAGCACTACGAGTCCGCGCTGGCCTACCCGGTGC

AGTCGCCGGGCGTCACCGGCAACCAGCCCGACATCGTCATGGACTCGCTCTCCGTGCTCG

GCCTCGGCCTCGTGCACCCGCGCAAGGTCTACAGCTTCTACGACGAGCTCCACGCCTACC

TGGCCGCCTGCGGCGTCGACGGCGTCAAGGTGGACGTGCAGAACATCGTGGAGACCCTCG

GCGCCGGCCACGGCGGCCGCGTCGCGCTCACACGCGCCTACCACCGCGCGCTCGAGGCCT

CCGTCGCCCGCAACTTCCCCGACAACGGATGCATCTCATGCATGTGCCACAACACCGACA

TGCTCTACAGCGCCAAGCAGACCGCCGTCGTGCGCGCCTCCGACGACTTCTACCCGCGCG

ACCCCGCGTCGCACACCGTCCACATCTCCTCCGTCGCTTACAACACGCTCTTCCTCGGCG

AGTTCATGCAGCCCGACTGGGACATGTTCCATAGCCTGCACCCGGCGGCGGAGTACCACG

GCGCGGCGAGGGCCATCGGCGGCTGCCCCATTTATGTCAGCGACAAGCCCAGGAACCACA

ACTTCGACCTCCTCAAGAAGCTGGTGCTCCCCGACGGCTCCGTGCTCCGCGCACAGCTCC

CCGGCAGGCCCACGCGCGACTGCCTCTTCTCCGACCCGGCGCGCGACGGTGCCAGCCTGC

TCAAGATATGGAACATGAACAAGTGC

>Contig389

ACTCAAGGGAAGTGAATCACACCGGGTGATCGTAACATATTCCATTCAGGTGGGCAGTAG

AGATGCTGGGGGCTAGGAGATCGTTCTCTAGGCTCGCCGCGGCGGTTAGGACGCCGGTGG

ACGTGCCGAGAATGCCGGCGTTCGACCACGTGCCGCTGCCCTACGACGGGCCGAGCGCCG

TCGAGATCGCCAGGAAGCGCGCCGAGTTCCTCAGCCCCTCCCTGTTCCATTTCTACTCCA

AGCCCCTCAACATTGTGGAGGGGAAGAAACAGTACCTCTACGACGAGCACGGGCGGCGCT

ACCTGGACGCGTTCGCCGGCATCGCCACCGTGTGCTGCGGCCACACCCACCCCGAAATCA

TCGACGCCATCACCGCGCAGGCCAACCGCCTGCAGCACTCCACCGTGCTCTACCTCAACC

ACGCCATCGCAGACTTCGCCGAGGCGCTGGCCGCCAAGATGCCCGGCGATCTCAAGGTCG

TCTTCTTCACCAACTCCGGCACGGAGGCCAACGAACTGGCCATCATGATGGCGCGGCTGT

ACACCGGCTCCCACGATATCATATCTCTCCGCAACTCCTACCACGGCAACGCCTCCGGCA

CCATGGGTGCCACCGCACAGAAGAACTGGAAGTTCAATGTTGTTCAGAGTGGCGTGCACC

ATGCCGTCAACCCGGACCCCTACAGAGGCGCCTTCGGCTCGGACGGCGAGAAGTACGCGC

GGGACGTCAAGGAGATCATCGAGTTCGGCACCACCGGCCACGTCGCCGGCTTCATATCCG

AAGCTATCCAGGGTGTTGGTGGGATCGTGGAGGTGGCGCCTGGCTACCTGCCCCTGGCGT

ACGACGCTGTGAGGAAGGCCGGCGGCCTCTGCATCGCCGACGAGGTCCAGGCGGGCTTCG

CGCGTGTC

>Contig390

GGGAAGCTTCTCCACCAGAGTCCCCCAAGCCCATGAACGACTAGTCGCTGCGAAGCACCA

ACCCTAGAACCCAACCCTGCTCTCCCCCTCCTCGGCTCCTCCCCCTCGCGAGCGGCGACG

AAGAGGCCATGGATACCTAGATCCAGGGGATGGCGACGCACGGCGGCCGCTACGTGCTCT

ACAACGTCTACGGCAACCTCTTCGAGGTCGCCTCCAAGTACGCCCCGCCCATCCGCCCCA

TCGGCCGAGGCGCCTACGGCATTGACTGCGCGGCGGGTAGTTCGGATACAGGAGAGGAGG

TTGCGATCAAGAAGATTGGAAATGCGTTTGACAACCACATCGACGCCAAGCGGACGCTGA

GAGAAATAAAGCTTCTTCGCCACATGGACCATGAGAATATTCTTGCCATAAAGGATTTAA

TACGCCCCCCAAGAAGAGATGATTTTAAGGATGT

>Contig391

TTGGCAGGCACACCATGTCCATCTCCACCGCCTCCCGCCGCGCCCTCAGCCGGATCGGCG

GCGCCCTCCGGCGGTCCTTCTCGTCGTCGTCATCGTCGGTGCCGGACGCCTCGGGGTACC

ACGTGTCCGGCGGGCCGAGCTTCATGCGCGCGGCCGTGTTCTGGGAGCCCGGCCGCCCGC

TCACCATGGAGGAGTTCCGCATGCCGCGCCCCAAGGCCGGCGAGGTCCTCGTCAAGACCA

AAGCTTGTGGAGTCTGCCACTCTGATCTCCATGTCATGAAAGGCGAACTCCCTTTCGCCA

GCCCTTGTGTCGTTGGCCATGAGATCACCGGGGAGGTTGTCGACCATGGCATGCACACGC

CCGCTGAGATCGTCAATAGGTTCCCAGTCGGCAGTCATGTTGTTGGAGCCTTCATAATGC

CCTGTGGGAATTGCTTTTACTGTGTGAAGGGCCAGGAAGACCTCTGTGAGTCTTTCTTTG

CATATAATCGTGCTAAAGGAACACTATACGATGGTGAAACCCGACTGTTTCTACGCAGCA

ACGGAAAACCAGTGTACATGTACAGTATGGGCGGGCTTGCAGAATACTGTGTTGTGCCGG

CCAATGCACTAGCAATTCTTCCTAACTCGTTGCCAGACACAGAGTCAGCCATTCTAGGAT

GTGCTGTGTTCACCGCATATGGCGCTTTGAGGCATGCTGCTGAAATGCGTGCCG

>Contig392

GCACCACCACAAGGACCAGGCGCCGGCGGCGTCGGGCCCCAACCAGATCTTCAAGATCTA

CTGCCGGGCCTCGGAGGACTACTGCCTCGCCGTCCGCGACGGCGAGGTGGTGCTCGCCCC

CGTCAACCCCAAGGACGAGACCCAGCACTGGCTCAAGGACATGCGCTTCAGCACCACCGT

CAAGGACGAGGAGGGCATGCCGGCCTTCGCGCTCGTCAACAAGGCCACCGGCCTCGCCGT

CAAGCACTCCATCGGCCAGTCCCACCCCGTGAAGCTGGTGCCGTTCAACCCGGCGTACGA

GGACGCGTCGGTGCTGTGGACGGAGAGCAAGGACGTGGGCAAGGGCTTCCGCTGCATCCG

CATGGTCAACAACACCCGCCTCAACTTCGACGCCCTCAACGGCGACAAGGACCACGGCGG

CGTGCACGACGGCACCACCGTCGTGCTCTGGGAGTGGTGCAAGGGGGACAACCAGTGCTG

GAAGATCTGGCCCTGGGCCGAGGCGCACGCCGCCGTCGAGTCCGGCGCCACCATGGGCAA

CAACGCCCACGCCATGGGCGGCGGCCCCCCCGTGCATGCCGTGCGCGTCTTCTGCAAGGC

CGGCGAGGACTACAGCCTCACCGCCCGCAACGGCACCGTCTGCCTCGCCCCCACCAACCC

CAGGGACGACTACCAGCACTGGATCAAGGACATGAGGCACAGCAACAAGATCAGGGACGA

GGAAGGGTACCCTGCGTTTGCGCTGGTGAACAAGGTCACCGGCGAATGCATCAAGCACTC

CACCGGCCAGGGCCACCCCGTGAAGCTGGTGCCGTACAACCCGGCGTACCAGGACGAGTC

GGTGCTGTGGACGGAGAGCCGAGACGTGGGCAAGGGCTTCCGCTGCGTCCGCATGGTGAA

CAACATCTACCTCAACTTCGACGCCTTCCACGGCGACAAG

>Contig393

TCGGCCGAGGGTTTGCCTTTATTTATAAATATTATACTACTTGCGAAATGCCATTATTCC

TCACTGGATATAGGGGCAGATGCAGCAACATCCGCTGAGATACAAGGCCCACAGCACTCG

ATAAAAGGAAAATATCTCATCAAGGTACAAAAAGAAGATAATCCGGCAATCTCCCCTTGC

CCTAACCGGTGTTCTGGAGACCGGCAGCAATGCCCTTCATGGTCAAGATGAGGGTATCCT

CCAGCCCCGGCGCGTACTCACTCGCCGGGTTCAGCGTCACGAGCTCAGCAGCCGGCTTGC

TCGTGTCCATCACCTCCTTGGACAGATGGGGGCGCAGCGCAACATGGTAGTCTGGGTCAC

GGATCCTCTTCAATGTGTATGCCTGGCAGACGTTCATGGTGGTGATGTACGCGTCACGGA

GGCGGAGCCGCTGCTTCAGGTAGGGATCACCTTCAAGAAGATCCTTGTGCCCAGCAACCT

GAAGAAGCAGCTTCTGGGTCTCCTCATAGTTGGCCCTCAGCTTCTCACCCAGGGGCTGTA

GCCCCTCTGAAACTAGGAGCCTGTAATACAAGGCAGCAATGCCAGGATTACCCTTGGCGA

ACACCATCTCAACAAGATCGATGGTGACCCTGAAGAATGGCCACTCGTTGTACATCTCCT

GGAGCATGTGGAAGTTCCTGATGTCCTTCTTGAGGATATGCTTGAAGGCACCACCAAAGC

CCAGCCAGACCGGGA

>Contig394

GAGGCTTGGTTTATCTCATGGGTTTCTCCTTGGTCATCTGCAATCACATGGCCTTGATTT

CCCCAAAAACATCAGCGTCGTTGCTGTGTGTCCCAAGGGGATGGGCCCGTCGGTTCGGAG

ACTGTATGTTCAGGGCAAGGAAGTAAATGGTGCTGGCATCAACGCTAGCTTTGCTGTCCA

CCAGGATGTTGATGGAAGGGCCACTGATGTTGCTCTTGGATGGTCGGTTGCACTAGGATC

CCCATTCACCTTTGCTACTACTCTAGAACAGGAGTACAAGAGTGATATCTTTGGGGAGCG

AGGAATTTTGCTGGGCGCTGTCCATGGCATTGTGGAGGCTCTCTTTAGGAGATACACAGA

GCAAGGAATGGACGAGGAGCTGGCATACAAAAGCACCGTGGAGGGCATCACTGGAATTAT

CTCTAAGACCATCTCAAAGAAGGGGATGCTTGAGGTGTACAACTCTTTGAGTGAGGAAGG

CAAAAAGGAGTTCAACAAGGCATACAGTGCATCATTCTACCCTTGCATAGACATACTCTA

CGAATGCTACGAAGATGTTGCCTCTGGAAGTGAAATCCGGAGTGTTGTGTTGGCTGGTCG

GAGGTTTTATGACAAGGAAGGCCTTCCTGCTTTCCCTATGGGCAAAATCGACCAAACTCC

CATGTGGAAGGTCGGCGAAAAGGTGCGCTCGACCCGGCCAGATGGTGACCTTGGCCCGCT

CCACCCCTTCACGGCTGGAGTTTATGTTGCACTTATGATGGC

>Contig395

TCGGCCGAGGCTCGGTCGGCCAGTGTGACGGACTCGTCGCGGTGGAGGGGGGAGAGGGGG

CTGAGCTCGCCGTCGATTCGAGGGGAGAGGAGGCGGGAAGAGAGGGGCGGCCACCCCGGG

GAAACCGAACCCTATCGGCCGCGGCCCCCCCTCCATCGACCGCCAGTCGCCGCCGGCCAT

GGCAGGGGCGGCGCCGGATCGGGCGGCGCTGACGGTCGGCCCGGGCATGGACATGCCGAT

CATGCACGACAGCGACCGCTACGAGCTGGTGCGGGACATCGGCTCCGGCAACTTCGGCGT

CGCCCGACTCATGCGCGACCGCCGCACCATGGAGCTCGTCGCCGTCAAGTACATCGAGCG

CGGGGAGAAGATAGACGAGAATGTCCAGCGTGAGATAATTAACCATAGATCACTGAAACA

TCCCAACATCATTAGGTTTAAGGAGGTTATTTTAACACCGACCCATCTTGCCATTGTCAT

GGAATATGCATCTGGTGGTGAGCTTTTTGAGAGAATATGTAAAAATATACGATTCAGTGA

AGATGAGGCTCGCTACTTCTTCCAGCAGCTTATATCCTGGAGTCAGCTACTGCCATTCGA

TGGTATCGGTTATTTACACAACCTATCTTTCACCTATTGTTACTATGTGTTCATATGTTG

>Contig396

GGCAGATGAAGCCCGAGCCTAAGCCAATGCCGAGCCGATGCCAAAACCAGAGCCAAAGCC

AGAGGCCCATAAAGCCAGAGCCTAAGCCGATGCCAAAGCCGGAACCGAAGCCAGAGCCGA

TGCCAAAGCCGAAGCCGGAGCCGCCTCCGAAGGGCAAGCCGCCAATGACTGACAATTGAT

GTGATACTCACATATGACAGTGGGAGGAGGAGATCGACCCCGTCCGGAGCCACGATGTGT

TATTTCTAGAATAAGTGGCGTAGTATCCGGCTAGCGAG

>Contig397

GCCGGCAGGGCCGCGTCAGGGTCGCACGGCCGCAGCGCCGCGGGCGAGCCGCGCGTCGCC

TGGAGGGCTCGTTCGGTGGCTCTGAGCGGCGTGAATCTCGGTACACACGGGTGAATTTAC

TGAATTACTAGTAGCAGTTAGCTGTGGTAGATTGGTGGGTGTAATCCAGGCTTCGAAGAG

GATGTAATCCAGAAACACGACTGGTCGTGAATTAGTGGTTTGGTAGTACTAGTGCTGATG

CACGTGTAATTATGGCGAATTTACGGCACCCTTGAACAGAGCTTTGCGAGGATCTTTTTT

ACTGCTAGTTCATATGGTGCTTTTTTTTCCCTTTGGTTGCGAGAATCGTGCTGTGATGTT

TTCTGCTGGATTTCTGTTTGCT

>Contig398

TCGGCACGAGGCTCCCGCACGCTTCTCCTCCGCCTCGAGCAGCTCAGGGACGCGCGCAGC

CATGGCGACGGAGACCGAGACCTTCGCCTTCCAGGCCGAGATCAACCAGCTCCTCTCGCT

CATCATCAACACCTTCTACTCCAACAAGGAGATCTTCCTCCGCGAGCTCATCTCCAACTC

CTCCGATGCCTTGGACAAGATCAGGTTCGAGAGCCTCACGGACAAGAGCAAGCTCGATGC

GCAGCCCGAGCTCTTCATCCACATCATCCCCGACAAGGCGACCAGCACGCTCACGATCGT

CGACAGCGGCATTGGCATGACCAAGTCGGACCTCGTCAACAACCTCGGGACCATCGCCAG

GTCGGGGACCAAGGAGTTCATGGAGGCACTCGCTGCCGGCGCTGACGTGTCCATGATTGG

GCAGTTCGGTGTTGGGTTCTACTCCGCCTACCTCGTCGCCGAGAGGGTCGTCGTCACCAC

CAAGCACAACGACGACGAGCAGTACGTGTGGGAGTCTCAGGCCGGTGGCTCCTTCACCGT

CACTCGTGATACGTCTGGGGAGCAGCTCGGCAGGGGTACCAAGATGGTGCTCTACCTCAA

GGACGACCAGATGGAATACCTTGAGGAGCGCCGCATCAAGGATCTAGTTAAGAAGCACTC

TGAGTTTATCAGCTACCCCATCTCCCTGTGGACCGAGAAGACCACTGAGAAGGAAATTTC

TATCAAGGAAGTCTCTCATGAGTGGAACTTGGTCAACAAGCAGAAGCCTATCTGGATGAG

GAAACCAGAGGAGATCAACAAGGAAGAGTACGCAGCTTTCTACAAGAGCTTGACCAATGA

CTGGGAGGAGCATTTGGCTGTCAAGCACTTCTCTGTTGAGGGTCAGCTTGAGTTCAAGGC

TGTCCTGTTTGTGCCCAAGAGGGCCCCCTTCGACCTCTTCGACAACAAGAAGAAGGCCAA

CAACATCAAGCTGTACGTGCGCCGTGTCTTCATCATGGATAACTGTGAGGAGTTGATCCC

TGAGTACCTGAGCTTTGTCAAGGGCATTGTTGATTCAGAGGACCTTCCCCTGAACATCTC

CCGTGAGACTCTCCAACAGAACAAGATCCTCAAGGTCATCAGGAAGAACCTTGTCAAGAA

GTGCATTGAGCTCTTCTTTGAGATTGCTGAGAACAAGGAGGACTACAACAAGTTCTACGA

GGCCTTCTCCAAGAACCTCAAGCTTGGCATCCACGAGGACTCCCAGAACAGGACCAAGAT

TGCTGAGCTTCTGAGGTACCACTCCACCAAGAGTGGTGATGAGCTGACGAGCCTCAAGGA

CTATGTGACCAGGATGAAGGAGGGACAGAACGAGATCTACTACATCACTGGTGAGAGCAA

GAAGGCTGTGGAGAACTCTCCCTTCCTTGAGAAGCTGAAGAAGAAGGGCTATGAGGTCAT

CTACATGGTTGACGCCATTGATGAGTATGCCATTGGTCAGCTCAAGGAGTTTGAGGGCAA

GAAGCTTGTCTCTGCCACCAAGGAGGGTCTGAAGCTTGATGAGAGCGAGGACGAGAAGAA

GAAGCAGGAGGAGCTCAAGGAGAAGTTCGAGGGGCTGTGCAAGGTCATCAAGGAGGTGCT

GGGCGACAAGGTGGGAGAAGGTCATCGTCTCTGACCGTGTTGTGGACTCTCCTTGCTGTC

TGGTGACTGGTGAGTATGGGTGGACCGCCAACATGGAGAGGATCATGAAGGCCCAGGCCT

TAAGGGACTCGAGCATGGCTGGCTACATGTCGAGCAAGAAGACCATGGAGATCAACCCTG

AGAACGCCATCATGGACGAGCTCCGCAAGCGCGCCGACGCCGACAAGAACGACAAGTCTG

TCAAAGACCTCGTGATGCTGCTCTTCGAGACCTCCCTGCTCACCTCCGGCTTCAGCCTGG

AGGACCCCAACACCTTCGGCACCAGGATCCACCGCATGCTCAAGCTTGGCCTGAGCATTG

ATGAGGATGACGAGGCGCCAGAGAACGACACCGACATGCCGCCCCTGGAGGACGACGCCG

GCGAGAGCAAGATGGAGGAGGTCGACTAAGCGCCAAGGCTGCCTGTCGGCTGTCGTCGTC

AGATGTCCATGCCCTGCACCGCCTAGTTGTTTTTATCTTAGTGTCGACGTTGGATGGTTT

TGGCACGTTTTAGCTGTATCGGTTTTGTCTTTTGGCTGGCCAAACATTAAGCGCGGTTAG

CTGTTTGT

>Contig399

CACACCGACACCCCATCCACTTTATCTCAGTAGAGAGGTTTAGAAGCAGTGAAGCACAAG

TGCAGCAAGAGCTGCGAGAAGAAGGAGCGAGCAGCAATGGCGAGGCACAGCCTCCTTGCC

GTGCTCCTCGTCGGGCTGGTCGCGGCCTCCGGCTTCAGCCAGGCGGCCGCCGCTGGCCGG

GGCCTCGCCGAGAAGCTCCCCGAGCCGGAGCCCAAGCCGACGCCGTACCCGGAGCCCAAG

CCGCAACCCAAGCCAGAGCCAATGCCTAAGCCTGAACCCATGCCAAAGCCAGAGCCTAAG

CCTCTGCCTAAACCTGAACCCATGCCAAAACCAGAGCCTAAGCCTCTGCCCAAACCTGAA

CCCATGCCAAAGCCAGAGCCCAAACCGGAGCCGAAGCCGGAGCCGATGCCAAAGGATGAA

GCCTGAGCCTAAGCCGATGCCAAAACCCGAACCGAAGCCAGAGCCCAAGCCCGAGCCGAT

GCCTAAA

>Contig400

GAGGCCGCCCACGGTCTAATCCGATTCAAACGCACCGCTTCCTCGTGTCCGAACTAAGCC

CCAATCCCACCACCACCGCCGCTGGTGCTCGGCGCGATGGCGCAGAAGGACGCCGCCAAT

GGCAACGGCGCCACCACGCGCCCGCCGCCCACTCCCTCCCCCATCCGCTTCTCCAAGTTC

TTCCAGGCCAACATGCGGATCCTGGTCACCGGCGGAGCTGGCTTCATCGGCTCGCACCTC

GTCGACAAGCTCATGGAGAACGAGAAGAACGAGGTCATTGTTGCTGACAACTTTTTCACT

GGGTCAAAAGACAACCTGAAGAAGTGGATCGGACACCCAAGATTTGAACTCATCCGTCAC

GATGTCACCGAACCACTGCTCGTGGAGGTTGACCAAATCTACCACCTTGCTTGCCCTGCC

TCACCAATCTTCTACAAGCACAACCCTGTCAAGACCATCAAGACGAATGTCATTGGAACC

TTGAACATGCTTGGACTTGCAAAGAGAGTTGGAGCTAGAATATTGTTGACTTCGACCTCT

GAAGTTTATGGTGATCCCCTTGAGCATCCTCAGACGGAGGCGTACTGGGGCAATGTTAAC

CCAATTGGAGTCAGGAGCTGCTACGATGAGGGTAAGCGTGTAGCTGAGACACTGATGTTC

GACTACCACAGGCAGCACGGCATTGAGATCCGTATTGCCAGGATTTTCAACACCTATGGA

CCTAGGATGAACATTGATGATGGCCGTGTTGTTAGTAACTTCATTGCTCAGGCCATTCGT

GGTGAAGCACTGACTGTCCAGAAGCCTGGAACACAGACTAGGAGCTTCTGCTACGTCGCT

GATATGGTCAATGGTCTTATGAAGCTGATGAATGGAGACAACACCGGACCCATTAACATT

GGGAACCCAGGTGAATTCACCATGCTGGAACTTGCCGAGAATGTGAAGGAGTTGATCAAC

CCAGAAGTGACAGTGACGATGACTGAGAACACTCCTGATGACCCTCGCCAGAGGAAGCCT

GACATCACCAAGGCCAAGGAGGTTCTCGACTGGGAGCCCAAGGTCGTCCTGCGCGACGGC

TTGGTGCTCATGGAGGACGATTTCAGGGAGCGCTTGGCAGTGCCCAAGAAGACCAAGGCC

TAAGCCCCCTCCCCGCGTTTGGCGTACAACATCACCAATTTGCCAGAGCATACTCATAGT

TGGGTGGGTTCGTCAATATTCGTTGCTGCGTTTCGTTACGGAATTTGAGTTCCAATAAAC

CAATTACACCTTCGTTGTTCACTTGAAAGATTGTATTATT

>Contig401

CGGCACGAGGGCCAGAGCTCCGCGGACAAGAGAGTGACCACACCCGCGCGAACACGTAGG

CGGAGCGGTACAAGATGCCAATGGAGAGGTCGGTGTCGCTGGCCGAGAGGAGCAAACGAC

CTCCGGTGCCACAGCGCCTCCTACGCCGTCTCCTACGCGCCCACCAAGGTGCAGCGCGCC

AGGAGCACCACCTCCCTGTCGCGCCCCGTGGCGGCGACCGCCGTGCAGCGGAGCGGCAGC

ACGAGGACCGTCTCCGGCGCCGGCCCCACCCCGGGGCTGAACCTCCGGTGCTACAGCGCC

TCCTACGCGGCCTCCTACAAGCCTTTCTCCGACGGCGCCGCCCAGGCCAAGGGGCCCAAC

GCCGCCACCACCACCGCAGCCACCTGGTCCTCCACCGGCCGCCGCTCCCTCAACCTGAGG

AGCTACACCCCCTCCTTCTCGGCATTGGTGGACAACGAAAGACGGACGAGGCGGAGGCGG

AGCTGCAGAGGAAGAAGCGGCTGGTGGCCTACAAGGTGTACGACGTGGAGGGCAAGGTGA

AGGGGTCGGTGCGGCGCACGTCAAGTGGATCAAGGTCAAGTGCTCCCGCGCCGTCTACGG

CTGGTGATGAGCCGGCGATCCGTCCATCCAGGTTGCTCTGCCTCCGTCTGCTC

>Contig402

CGGCACGAGGCGGGGAGGTCCAATATCCCGGCGAACAACTCCGCCCTCATCGCCATCATC

GCCGATGAGGACACTGTCACTGGGTTTTTGATGGCTGGGGTTGGCAATGTTGATCTGCGC

AAGAAAACAAATTACCTTCTTGTAGATAACAAAACGACAGTGAAACAGATTGAAGATGCA

TTTAAAGAGTTTACTGCAAGGGAGGATATTGCTATTGTGCTCATCAGCCAATATGTCAAA

ACGGTACTGCAATATTTGCTTGGCTGGCTGCTGTTGAATCATGGCAGTTTCTTGAAACAG

TTTGGATAAGTCTCAGTCGACTGAGACTTCATTATGTCTTAGTCGAATTCCTTTAAGCTT

GCATATACGATTCGTAAAAGAAAATTCTTCAGTTTTTTTTTCTTATCTACTTTGTCACTT

GACTGAGACTTGATCTAGCCATTCTTGATACGATTTCTCAGCTAGGCACATCAACCCGTT

ATGCATCAACATGATATTCGTTATGTATACAACTCCACGCTGTGCTACTTAAACTCCATT

GATCCGGGGTGATCAAA

>Contig404

GGCACGAGCCGGCAGCGGCGGCCGCGGGGGACGAGGAGCCCCTGATCCGGCAGGTCGTCG

GCGCCGCCGACCCCCTCGACAACGACCTGGAGCTCGACTCGCAGTTCCTCGGCTTCGTGC

AGCGGTTCGGGAAGACCTACAGGGACGCGGAGGAGCACGCGCACCGGCTCTCCGTCTTCA

AGGCCAACCTCCGCCGCGCGCGCCGGCACCAGATGCTCGACCCGTCCGCCGAGCACGGGG

TCACCAAGTTCTCCGACCTCACCCCGGCCGAGTTCCGCCGGACCTTCCTGGGCCTCAAGA

CCACCCGGCGGTCGTTCCTGCGGGAGATGGCCGGGTCGGCGCACGACGCGCCCGTCCTCC

CCACCGACGGCCTCCCCGAGGACTTCGACTGGAGGGACCACGGCGCCGTCGGCCCCGTCA

AGAACCAGGGTTCGTGCGGGTCGTGCTGGTCGTTCAGCGCGTCCGGGGCGTTGGAGGGAG

CCAACTACCTGGCCACGGGCAAGATGGAGGTGCTCTCCGAGCAGCAGCTGGTCGACTGCG

ACCATGAGTGCGACCCAGCAGAACCTGATTCATGCGATGCTGGATGCAATGGTGGGTT

>Contig405

CGGCACGAGGGTTCCCTGGCTGCAAACCCTTGTCGTATCAAGGTTGAAGTTCCCCACGTT

AGTCTCCCGGACTTACGAAATGAAATTACTCGGCGAAGAGGAGGCTCTCTCTGTCTTCTG

CAGTGCTGCCTTCGGTCAGGAGTCTGTTCCTCAGACTGCTGACAAGAAACTGGTTAAGCA

GGTTGCTGCCGAGTGCAGAGGGCTTCCTCTAGCTCTCAAGGTTATCGGCGCATCCTTGCG

TGATCAGCCTCCTATGATATGGTTGAGCGCGAAGAACCGCTTGTCACGAGGAGAGTCTAT

ATCGGACTCACATGAGACCAAACTCCTCGAGAGAATGGCAGCAAGTGTCGAGTGCTTGTC

GGGAAAGGTTAGGGAATGTTTCCTTGACCTGGGTTGCTTCCCAGAGGATAAGAAGATCCC

TCTTGATGTGTTGATCAATATTTGGATGGAGATACATGATCTTGACAAGCCAGATGCTTT

CGCCATCCTCATGGAGCTATCGAACAAGAACCTGCTTACCCTAGTTAATGATGCACAGAA

TAAAGCTGGAGATTTATACAGTAACTATCATGACTACTCAGTGACGCAACATGATGTGTT

GCGTGATCTGGCACTTCACATGAGCGGTCGTGATTCTCTGAACAAAAGGAGGCGGCTAGT

GATG

>Contig406

GGCACGAGGCCATCCACCAAATCCCCAAAACCCTAGCAGTGAAATCGACTCGATCGAAAA

AGTTTTTCGTCTCCCTCTCGCGGAATCAGCAGCGCACCGGTCGATCCCCAGCGGAGCAGC

AGCAGAGATGGCGCAGTCCGGCAACAACGACGCCGCCCCGATCAGCACCCAGCCCGCGGC

GGAGGAGGTGACGGTGGAGAGGACGCCCGAGGAGGAGGAGGCCAGGCTGAGGTACCTCGA

GTTCGTGCAGCAGGCGGCGGCGCAGGCCGTCGTGCTGGCCGCCGCGGCCTACGCGTACGC

CAAGCAGGGCGCCGGCCCGCTCCGCCCCGGGGTCGACCACGTCGAGGGGACGGTCAAGGC

CGTCGTCGGCCCCGTGTACGACCGCTACCACGCCGTGCCGCTCGACCTCCTCAAGTTCCT

CGACCGCAAGGTCGACCAGTCTGTCCAGGAGCTGGATCGTCGTGTCCCGCCGGTCGTGAA

AGAGGTGCCGACCTATGCTCGCTCTGCAGCAGCTGAGGTGCAGAAGACAGGCATAATGGG

CACAGCAACTGGCCTGGCCAAGACGGCCATTGCTCGTGCTGAGCCAAAGGCCCGTGACTT

GTATACCCGCTATGAGCCTGTGGCGGAGCGCAAGGCTGCTGAAGCCTGGGCCGCCCTGAA

CCGCCTCCCGCTCGTCCCCTCGGTGACCCGTGCTGTCCTCCCCACGGCAGCACAGCTCTC

AGCCAAGTACAACTCTGCCGTGCTTGATGGCGCGAAGCGTGGCAACTCCGTCGCCACCTA

CCTCCCGCTCGTCCCAACTGAGCGCATTGCAAGGGTTTTCGCCAACCCGGCCGCCGACAC

TGCGCCTGCCACAGCTCCTGAGATGCAGCCCATCCCGACGCAGTAACAATAAGAGCTACC

CGGAGTTCTATTATTACCTAATAGCTGTAGGAGTCAGTATCTCGTTGTAGTGTCTTGTTT

GGCTTGTGGTGGACAGTGGTATTATGTGTGGATGGTCACTGTGGTATCCGCCTGTGTATG

GAGGCGAGAATGCTGCTGCTGTGCGGCAACACCTGCTTTGCGTTTTAGTTTGTGCTGCAT

GAACCACTGTGTGTAATGCTTTGAATCAAAGTTACATGAAACACCATCTGTAAATTCATA

ACTCTTTTT

>Contig408

GGCACGAGGGCTTGCCCCCCTTCATGTTGTTGCATTGCCCTCGGGGAGACGAGGTCATTA

GTTGATCACCAGCAGTCGATCGGTTGGGGATCAATCGAACAGAAGGCAGTCGGTGATCTC

TTGATTGATTTGGACGCCATGAAATTCGGAAAGTGGCTCAAGCGGCAGATCGAGCAGAGC

CTCCCGGCGTGGAAGGACGAGTTCCTGAGCTACAACGAGCTCAAGCCCGTCATCGGCGCC

GTCTCGCCGGCCGAGTTCGTCGCCCGCCTCGACGTTGAGATCGAGAAGATCAACGCCTTC

TTCATCGAGCAGGAGGAGTTCTTCATCATCACACACCGGGAGTTGCAGGGGGCGATCAGG

AGCGCGCTGGAGAGGAAGCCGGCGGTGCCGGCGTCGGCGCACGAGGCGGAGATCGCGGCG

ATCCGGAGGGAGATCGTCAACTTCCACGGCGAGATGGTGCTGCTGCTCAACTACAGCAGC

GTCAACTACATCGGCCTGGCCAAGATCCTCAAGAAGTACGACAAGCGCACGGGCGCCGGG

ATCCGGCTCGCCGGGATCGAGACCGTGCTGGTGCAGCCCTTCTTCA

>Contig410

GGCACGAGGCAAACAGCAGCATACTAGATTTTGATTCCCCCTGTGACAAGCTCAGCTCAG

CGCAGCGCAAGATGGAGTACCAGGGACAGCAGCAGCGTGGCCGCGTCGACGAGTACGGCA

ACCCGGTGGCCGGACATGGCGTCGGCACCGGCATGGAGACGCACGGCGGCGTCGGCACCG

GCGCGGCCGCCGGTGGGCATTTCCAGCCCATGAGGGACGAGCACCAGACCGGCCGTGGGA

TCCTGCACCGCTCCGGCAGCTCCAGCTCCAGCTCGTCTGAGGATGATGGCATGGGCGGGA

GGAGGAAGAAGGGCATCAAGGAGAAGATCAAGGAGAAGCTCCCCGGTGGCCACGGTGACC

AGCAGCACACCGGTGGCACCTACAGACAGCAGGGTACTGGCATGGTCGGCACCGGCGGCA

CCTACGGGCAGAAGGGTCACACTGGGATGACCGGCACCGGCGGCACCTACGGGCAGCAGG

GTCACACTGGGATGACCGGCACCGGCGGCACCTACGGGCAGCAGGGCCACACTGGGATGA

CCGGCACCGGCGGCACCTACGGACAGCAAGGCCACACCGGGATGGCCGGCACCGGGGCGC

ATGGCACCACGGCCACTGGTGGCACCTACGGGCAGCCGGGCCACACCGGGATGACAGGCA

CGGGGGCGCACGGCACCGGAGGCACCTACGGGCAGCACGGCACTGACACCGGCGAGAAGA

AGGGCATCATGGACAAGATCAAGGAGAAGCTCCCAGGCCAGCACTGAGCGCTGAGGAGAG

CCCGCGGCCGCCACTTCTGAGAGTGGAGGTGCCGGTCGACCACCGTTGCAGAATCAATAA

TAAGATCGCGATACGATACAATAAAATTCCACCATACAACGTGAGCCTAGTTCACCTAGC

TCACTTGCGTGTTGGAGGAGCCACTGTATCTAGGCTCAAGTTTACGTGAACAAACAGTGT

TTTGAGTTTTTCGTCTGTTTATTACACTGTATAATCTTGTAAGTTTCCTGTGGTTAAACC

CTGTATGTACGCTTTA

>Contig414

GATTTCTAAGGGAATCAAGATCAGCATCTGTGCAAGATGGAGCACGGCCAGGCGACTAAC

CGCGTCGACGAGTACGGCAACCCGGTGGCCGGACATGGCGTCGGCACCGGCATGGGGGCG

CACGGCGGCGTGGGCACCGGCGCGGCCGCTGGTGGGCATTTCCAGCCCACGAGGGAGGAG

CACAAGGCCGGAGGGATCCTGCAGCGCTCCGGCAGCTCTAGTAGCTCCAGCTCGTCTGAG

GATGATGGCATGGGCGGGAGGAGGAAGAAGGGCATCAAGGGGAAGATCAAGGAGAAGCTC

CCCGGTGGCCATGGCGACCAGCAACAGACCGCTGGCACCTATGGGCAGCAGGGTCACACC

GGAATGACCGGCACCGGGGCGCATGGTACAGCAGCCACTGGCGGCACCTACGGGCAGCAA

GGACACGCCGGAGTGACCGGCACGGGGACGCACGGCACCGACGGCACTGGCGAAAAGAAG

GGCATCATGGACAAGATCAAGGAGAAGCTGCCCGGACAGCACTGAGCGCGGCCCGCGGCA

GCTACTTGAGGAGTTGGGGTGCCGAGCTGGCCACCTTTGCAGAATAATAAGATGGAGATA

CAGTAGAACTTCCCGAAATAAAGTGAGCTAGCTCACTCGTAATATCCGAGTTCTGAGTTT

AGTGGACTTGAATTTGGGTTGCTTGC

>Contig415

GGCACGAGGCCTTTATGTCAAGGTTAGCATGGATGGTGCTCCTTACCTCAGGAAGGTGGA

TCTCAAGATGTATAAGAACTATAAGGACCTCTCGCTGGAGCTGGAGAAAAAGTTCAGCGG

CTTTACTGTTGGTCATGGTGAATCGACTGGAAAATCGGGAAGAGATGGATTATCTGATTG

CCGGCTGATGGACCTTAAAAGCGGCACTGAACTTGTGCTCACTTATGAGGATAAGGATGG

TGATTGGATGCTTGTTGGTGATGTTCCATGGCGAATGTTCACAGACAGCTGTAGGAGGAT

GAGGATCATGAAGGGGTCAGATGCAGTGGGCCTCGCTCCGAGGGCCGCCGAGAAGAGTAA

GAACCAGAAGTAGCAAGAACGATGAACCCGATCTGTTTCCTTTCCTGCGATCGCATCTTA

CAAGGGTGATGGACTTTATGTGTGATGAGAAAGGGATTTACTTATATATATAACCTACGT

GTATTCTCTTTGTTATTTCCTTGTATGTTCGTTCGGAACAAGCATGTTAGATTGTGTAAG

CTGATGTGTGTGTATCTACTACTACTGCACTACTTTGAT

>Contig416

CGGCACGAGGGTGGTTTGCTTGATGGAGTGGGTACTCAACAGCAAGCACTACACAACCAA

GGTTATATCGGCAGTGGTTGTCGTCGCAGCGGGCGTCGGGATTTGCACCGTCACCGATGT

GGAGGTCAATGCTAAGGGCTTCATTTGCGCTTGTGTGGCTGTGTTCTGCACGTCGCTTCA

ACAGATTACAATTGGCTCCTTTCAGAAGAAGTACAACATTGGGTCATTCGAGCTGCTGAG

CAAAACTGCACCAATACAGGCAGTGTCACTTATTATACTGGGTCCCTTTGTGGATTACTA

CCTAAATGGGCGTTGGCTATTGAACTACGGTTTTTCAACAGGAGCAACTGTAAGTTCTCA

CTCCTGCCACATAATTTTCAGTTAATGAAACAATGCCTTGTATCTTCTGCTATAGATTCG

TGCCAATGTTAACTTCAGTATTTGTATTTATGACATTTTCCATTCCTCTACTGCTCAGTA

CCCCTCTGTAATAAGTTTGGAATTTCCTTTTCCAATTCATCTGATCCTGTCATGGAGCTG

TAACAACACTGATTTTTCTCTTATTTGTTTTGTATGTTTTGTATAACATCCTTCTTAAGA

AACGCGTAATTTATATTATATGACTTGCGGTTTGCTGTTAGCCACAACAATACTTCTAGC

TTGTGGTTATAAATG

>Contig418

TCGTCGGCGACGACGAGGTCACCTCGCACCGCTTCACGCCCGCAAGGATGTCGCACGTCT

CCTCGATCAACCCGGACGACTTCGACTGCGTCAGCGAGCCCATCATCAGGAGCGCCAGCG

TCAACGGCGACCACCTGCGCAGCAGGAGCGTCAACTCCAACGCCAACGCCGCCGACGCCG

ACATGCAGTTCTCCATCAGGTCCCTCCGCTCCTCCAGCATGTCGCACGAGATGGTCGAGG

TCTCCACGGTCCCCGTCCTCATCGACGGCGCCTCCTCCAGGAAGTTCACCAGGACCGCCA

GCCAGCAGAGGAGCGTCATCATCGAGGACGCGCCGCCGTCGTCGCCCACAGACGACGAGG

CCGCCGCCAACGGCGACAAGGACAAGGGGGAGCTGCCCGAGGTCGTGGAGAAGAGGTGGA

AGGTGCTGGTGTGGAAGACGGCCGTGTACCTCATCACCCTCGGCATGCTCATCGCCCTCC

TCATGGGGCTCAACATGTCCTGGACCGCCATCACCGCCGCTCTCGTTCTCCTCGCGCTCG

ATTTCACCGACGCACAGGCCTGCCTCGAGAAGGTGTCCTACTCGCTGCTCATCTTCTTCT

GCGGGATGTTCATCACGGTGGACGGGTTCAACAAGACCGGCATACCCAACACGATGTGGG

AGCTGGTGGAGCCCTACTCGCGGATCGACAGCGC

>Contig419

GGCACGAGGCTCCGAACGGCAGCTCGGGTCGTCGACCCGAGAGAATCTGCGAGGAGCAGC

AGCGGAGAGGAAGAAGACGGTGGTCTCTCTCCGCCATGGCCAGCTCGTCTGCCGACCCGG

AGAAGCTCATGGCCAAGGCCGACAAACTAACCAAACTGAGCTTTACAAGATGGAATGCTG

ATTGGAAGAGTGCTACCGCCTTGTACGAACAAGCTGCAATTGCTTATAGATTCAGAAAGG

ACAATGAGAAAGCAAAGGATGCATTTGAGAAGGCTTCAAAAGGACAAGAAATGATCTCAT

CACCGTGGGATGCTGCTAAGCATATGGAAAATGCTGGTGCTTTAGCAAAGGAGCTTGGAC

TATGGAATGAAGTTTCGGACTTTTATCGCAGAGCATCAGAATTTTACCGTGAATGTGGAA

GATCACAACCTGCCTCTGATGCTCTTGCAAAGGGTGCCAGTGCCTTGGAAGATAAAGCTC

CAGAAGAAGCAACTAAATTGTATGATGACGCTTGCACACTTCTAGAAGAAGATGGAAAGG

AGCAGATGGCTTTTGACTTGTACCGTGCTGCTGCAAGTTTATATGTGAAGCTTGAGAAGT

ACTCGGATGCCGCTGCATTCCATTTAAGACTTGGTTCAGCTGCTGATAAGTGCAATGCCG

TCAACAGCCAATGCAAGGCCTATCTGAGTGCAATCATTATCTACCTTTATGCACATGATT

TTCAACAAGCTCAGAAATGCTACAATGATTGCT

>Contig421

CGGCACGAGGGAATTCCTCCCACCCTCGCCGTCTCGCCGCCGTCACCACCGCAGCTAGAT

AAGAGAAAAAAAGAGAGAGAAGAACTCGTCGGGAGGGCTCGAGATCTGTGTTGGGAGAGG

GGAATTCTTGAGGTCGGAATCGGAACAAGCGGGCGCGCTCGGGATCGGGGTTACCATACA

ATTTTTCCCAGGAACCTTTAGTGAATGTGCTAATGGAGTCAAAGGGTGGCAAAAAGTCTA

GCAGTAGTAGTTCCCTGATGTACGAAGCTCCCCTCGGCTACAGCATTGAAGACGTTCGAC

CTGCTGGAGGCGCCAAGAAGTTCTCTGCTGCATACTCGAACTGCGCGAAGAAGCCATCCT

GATATCGTTTTTGGCATCCCCTTCCCGTAGTTTAGGATTTTTATGCAATTTTATTCTGAC

TCTTTTCTCCCACCAATCTCTCTGGCTTGCTGCTTCATCTGTTCCTCTCTGCTCTGCTTT

CTGACTCAAAACTGCAACAATGGCTGCCCCGACCTCTGCGATCGGATTTGAGGGCTACGA

GAAGCGCCTTGAGATCACCTTCTCTGAGGCATCAATCTTTGCTGACCCTCATGGTCGTGG

CCTGCGCGCCCTCTCCAGGGCCCAGATTGACTCTGTTCTTGATCTTGCACGGTGCACCAT

TGTGTCCGAGCTCTCCCACAAGGACTTTGACT

>Contig422

GAGGCTTCGGGCCTTCACATGGATCGGCTATGAGCTGGCACGTCCGGATGAAGATCGCGC

TCGACACGGCCAGGGGATTAGAGTATCTTCATGAGCACTGCAATCCACCAGTCATCCATA

GGGATCTGAAATCGTCTAATATACTCTTGGATTCAGACTTCAATGCTAAGATTGCAGATT

TTGGCCTTGCAGTGACAAGTGGGAATCTTGACAAGGGGAACCTGAAGATCTCTGGGACCT

TGGGATATGTAGCTCCCGAGTACTTATTAGATGGTGCGGCATTCCTTTTGTGCTAAAATT

TTACTTCTATTTGTGTTACGAAATTTAAGCTCTGAACCGCAAAATTCTCCTTTCACCATG

TAACTATTTAGCGGCCGTGTCAATTTCAGGGAAGTTGACCGAGAAGAGCGACGTCTACGC

TGTTTGGAGTTGTTCTTCTAGAGCTCTTGATGGGGAGGAAGCCTGTTGAGAAGATGTCAC

CATCTCAGTGCCAATCAATTGTGTCATGGGCCATGCCTCAGCTAACCGACAGATCGAAGC

TACCCAACATCATCGACCCAGTGATCAAGGACACAATGGACCCAAAGCACTTATACCAAG

TTGCGGCGGTGGCCGTTCTATGCGTGCAGCCCGAACCGAGTTACAGACCGCTGATAACAG

ACGTTCTCCACTCCCTTGTTCCTCTGGTACCCGCGGATCTCGGGGGAACGCTC

>Contig423

CGGCACGAGGGCCCACCTTCCTCTCCCCTCGCACCCCCTTTCCCTTCCAGCCCCGGGCAG

CGGATCTACCTGCCGCCGCCTCGATCCCGCCTCTCGGCGACCTTGCGCAGTCGCCGTCGC

CGCCGCCCTTCTTACTCGGGCCCCGGGGCGTCCTGCGGCTGACTCCACCCCGTCCCAGTC

TTCGTCCGGCCACCGGATCTCCTCCCTCCCTCTGAGTCTGAGGGTGGGGATGGGGCCTTC

GAAATTTCCGGGCCAGATCCGCCCCTGGCAGGGTCATGCCTGCTGTTGTGTTCCTGGATT

CCTTCTTGTGGATGATATGTGTATGATTCGGGTGCTGTCTACTCTTGCGTGGTTACAAAT

TTCGGCTTCTTTTACAAGGGATGATGATGATAGCTTGGTGCTTTTTTTATTATGATTTTG

AAGGAAGGACAGAAGCCGAACCATGGGGAAGAGGAAGGCGGCCGCTAAGCCACCCCCTAG

GAAGCGGATGGACAAGCTCGATACCGTCTTTTCCTGCCCATTCTGCAACCATGGGAGCAG

CGTTGAATGCCGGATCGATCTGAAGAATCTGATTGGTGAGGCCAATTGTCAAATCTGCCA

GGAAAGCTTCAGCACCACTGCAAATGCGCTCACTGAAGCTATTGATGTATACAGCGAATG

GATCGATGAGTG

>Contig424

CGGCACGAGGCCTCAGTGTATCTTATCCGCTTCCTTTCCAGAGCGGCGGCGGAGGACAAG

AAGGTGAAGGGCGGCGCGGCGCTCTCCGACTTATGCCTCCACACCACCTACGTGTCGTCG

TTCGTCTGCGCGGTCGCCGGCGGCTACCGCGCCCTGCGCGAGCGCTCGAACTGCTGGTGG

AGGGAGAGCATTCGTGGAGGAGGCAGGGTGAGTATGCCGTGCGCCAGCGCCGTCGTCGTC

CGCAGCACGGTCGCGGATGAAGCTCTGGATGGTGCGCGCCGCCACGACCATGTTGCTCTG

GACCTGCGTCATGCAGCTCACCGCCGTCGGTGAGACGTGGGGCCCCCAGGTTCTCAAGGG

ATGGCCCTCCCGCCTCACGGCCCCCGAGGAGGCTGCCGCCGTGCGCCCGGCCGTCATCGA

GAGGGCCGCGTTGCCATCGCGCAAAACATAGATTTCATCAACCTGGGAATGGGATGATAC

AGATTGGTGGTTGGCGTCGATTTTGCTCGCTTCACTGTGTAACATCGAGTCTCCACTGCC

AGTCCTCTACGATCTAACCAATCAGGCCTCCATCGACGTAAGCTTCTTAATCTCCGATAT

CCTGCTCCGTGTCTGATTTGGATTGAATTCGTGACCGTGTCCTACTGTCCTTCTCCAACA

CAAATCAATGGATTGCCTGACCCGGATTGGACTTCCCTCCTATAAGAAGGAAGGGTCCTC

GACCGGAATCTCCATCAGGTTGCGTACTTGGACCGGAAAATCCATCAAATCTCGGAATAC

TTGGACGAGAAAAGCATCATGTCGCCTTTGACTTCGCCGGAGGTAGAAGAACATCCGTCG

AGCCGTCTAATATCGCGGCTACAGCAAAGGTTCAAGGTGATCAGGTACGGCTCATGGGCT

GCTACAACTATTAGTGGGACAAAAGGGTTGTGCATGCACATGCTTATTGATTGAAGGCAT

AATTATGGGAGATGGTGCATGGTCCATATGAATAGATGGAACCCGTCGAGAGAAAAGAAA

AAGAAGAATAAGAAGAAAATTGTTCTTCGTAAACAAGTACTCCCTCCATCCCACTTATGT

TGGGACGGAATACATATTACATCATATGTAACTGGAGAATGGCGGCATGTCACGATCAGG

CAAGAACTCTGATGCCGGTGTGCACTTGCTGAATATAGATGATTGTGATGGCCTTATTCC

TTGGATGTGTAGGGAGCTT

>Contig425

CGGCACGAGGCTTTTCTCTCCATATTTTCATCTGAAGATAAAGCACGTGTGAATATACGT

GACGCGCGTACTCTCCCCGGTCCTGCTATTATCCACTTCGTCGTCGTCCTCGTCGTCGGC

GAGATCCCGCGCTTGATGCGGCCGTGAAGCCACACCAATGGCCGCGCAGCCGCCCAGCTC

CTCGCCGCAAGATGGTTCCGGCGGAGGCGGATCCTCGGAAAATCTGGTACTCCAAGCCCC

ACAAGCAGTGCGGGAGGATTACGTGCAGAACGCCGTCAATTTCCTCGCGCATCCGAAGGT

GAAGGGTTCACCGGTGTCCTACAGGTACTCTTTCCTGGAGAAGAAAGGGCTCACGAAGGA

GGAAATCGACGAGGCCTTCCGTCGGGTTCCTGATCCACCGACAAGCAGTACGGATGCTGC

GGCTGTTGGTTCGCAACAAGCAAGCAATCCAAATCAATCTGCCGGGGTGCAGCCTTACGC

GTCAGTTCAGTCGCCACAAGCACCAACTGTCTCTGTCGCCACTGGTCATATTGTCCCACA

AACGCAGACACAATTTAGCTGGTATCATACGCTCCTTGGTGCTGGTATTTTCCTTGGAGT

TGGAGCCAGTTCAGTTGTCGTCATCAAGAAATTGTTCCTGCCTAGGCTAAAATCTTGGAC

CCGTAGAGTCGTTTCTGAAGGAGATGA

>Contig426

CGGCACGAGGATCAGTTCTCGATTTCGATTCCGGGCTCAGAGAATTTCCCAGGCGAGCGA

GATGGCGACGGCCGGGAAGGTGATCAAGTGCAAAGCCGCGGTGGCATGGGAGGCGGGGAA

GCCGCTGTCCATGGAGGAGGTGGAGGTGGCGCCGCCGCAGGCCATGGAGGTGCGCGTCAA

GATCCTCTTCACCTCCCTCTGCCACACCGACGTCTACTTCTGGGAGGCCAAGGGGCAGAC

CCCCATGTTCCCTCGGATCTTCGGTCATGAAGCTGGAGGCATAGTGGAGAGTGTTGGAGA

GGGTGTGACTGAGCTTGCCCCTGGTGACCATGTCCTTCCTGTGTTCACTGGGGAGTGTAA

GGAATGCCCACATTGCAAGTCTGCGGAGAGCAACATGTGTGATCTGCTCAGGATCAACAC

CGACAGAGGTGTCATGATCGGGGATGGCAAGTCACGCTTCTCTATTGACGGGAAGCCGAT

TTACCATTTCGTAGGGACTTCCACCTTCAGTGAGTATACTGTCATGCATGTTGGTTGTGT

TGCCAAGATCAACCCTGAGGCCCCCCTTGATAAAGTCTGTGTTCTTAGCTGTGGTATTTC

CACTGGTCTTGGT

>Contig427

GAGGCCGCAGCAGCCGCCGCCAGCAGCTGCGCAAAGGCTCCACAAACCCTAACCCTAGCT

CCAAGCTTAGCTTCCATCCTCTCGATCTAGCTAGAGCCATGGCGCCGACCAAGGGCGAGG

GGCCGGCCATCGGCATCGACCTCGGCACCACCTACTCGTGCGTCGGCGTGTGGCAGCACG

ACCGCGTCGAGATCATCGCCAACGACCAGGGCAACCGCACCACGCCCTCGTACGTCGCCT

TCACCGACTCGGAGCGCCTCATCGGCGATGCCGCCAAGAACCAGGTCGCCATGAACCCCA

TCAACACCGTCTTCGATGCGAAGCGTCTTATCGGCAGAAGGTTTGCTGATGCCCCTGTCC

AGAGTGATATTAAGATGTGGCCGTACAAGGTTATTCCTGGCCCAGCAGACAAGCCTATGA

TTGTAGTGCAGTACAAGGGTGAGGACAAGCAGTTCTCAGCTGAAGAAATTTCTTCCATGG

TCCTCATCAAGATGCGTGAGATCGCCGAGGCTTACCTAGGAGTAACTATTAAGAATGCTG

TTGTCACCGTGCCTGCTTACTTCAATGATTCCCAGAGGCAGGCAACCAAGGATGCCGGTG

TTATTGCTGGTCTTAATGTGATGCGTATCATCAACGAGCCAACAGCAGCTGCAATTGCTT

ATGGTCTTGACAAGAAGGCTACCAGTGTCGGTGAGAAGAATGTGCTTATCTTTGACCTTG

GTGGTGGCACTTTTGATGTCTCTCTCCTTACCATTGAGGAGGGTATTTTTGAGGTGAAGG

CCACGGCTGGTGACACTCACCTTGGTGGGGAGGACTTTGACAACCGTATGGTGAACCACT

TTGTCCAAGAGTTCAAGAGGAAGCACAAGAAGGATATCAGTGGAAACCCCAGGTCTTTGA

GAAGGCTCAGGACTTCTTGTGAGAGGGCGAAGAGGACCCTTTCTTCCACTGCCCAAACCA

CCATTGAGATCGATTCACTCTTCGAGGGAGTTGATTTCTATTCCACCATCACCAGGGCCA

GGTTTGAGGAGCTCAACATGGACCTCTTCAGGAAGTGCATGGAGCCAGTGGAGAAGTGCC

TGAGGGATGCCAAGATGGACAAGAGCACTATCCATGATGTTGTGCTTGTTGGTGGTTCCA

CCAGAATCCCCAGAGTTCAGCAGCTGCTGCAGGACTTCTTCAACGGGAAGGAGCTCTGCA

AGAGCATCAACCCTGATGAAGCTGTTGCCTACGGTGCTGCCGTCCAGGCTGCAATCCTCA

GCGGTGAGGGGAACGAGAAGGTGCAGGACCTTCTCCTCCTGGATGTCACCCCACTGTCTC

TTGGTCTTGAGACTGCTGGAGGAGTCATGACTGTGCTCATCACCAGGAACACCACCATCC

CCACCAAGAAGGAGCAGGTCTTCTCCACCTACTCCGACAACCAGCCTGGTGTGCTCATCC

AGGTGTTTGAGGGTGAGAGGACAAGGACCAGGGACAACAACCTGCTCGGCAAGTTTGAGC

TCTCTGGCATCCCGCCTGCGCCCAGGGGTGTTCCCCAGATCACTGTCTGCTTCGACATTG

ATGCCAACGGTATCCTGAATGTCTCTGCTGAGGACAAGACCACTGGCCAGAAGAACAAGA

TCACCATCACCAACGACAAGGGCAGGCTGAGCAAGGACGACATTGAGAAGATGGTCCAGG

ACGCAGAGAAGTACAAGTCTGAGGACGAGGAGCACAAGAAGAAGGTTGACGCCAAGAACT

CCCTGGAGAACTACGCCTACAACATGAGGAACACCATCCAGGACGAGAAGATCGCCTCCA

AGCTGCCTGCTGACGACAAGAAGAAGATCGAGGACGCCGTCGACGCCGCGATCCAGTGGC

TTGACGCCAACCAGCTCGGTGAGGTTGACGAGTTTGAGGACAAGATGAAGGAGCTGGAGG

GCCTCTGCAACCCCATCATCGCCAAGATGTACCAGGGCGCCGGTGCTGACATGCCCGGAG

GCATGGACGAGGACGCGCCGGCTGCGAGCGGCGGTGCTGGCCCCAAGATCGAGGAGGTCG

ACTAAGTTAGTCGCTTTTATATAGTGAGAAATACACTATACACCATTTTATTCAGTCCTG

GGTGCCAATCATATCACCTGGGCTGGAAGTTTTGTTGTACTTTTTGGTCTGTAGCATCAC

GAGACTTGTGTGTGTCGTGAACAATGCAATGATGCATGTTTTGGCTCATTGTTGATTTCA

GCTTAATGCTTATTATTTGGACT

>Contig428

GGCACGAGGTGTCCCCATACGCCTCGCCTCTTGAGCGCGCTCTTGAAGGAAGGCTCGACA

CCTCTGCCTTCTGCTCTCCTCGGCTCTGCATCCGTGAGAGCTGTTGCATTGCTGCAGCTC

GCGAGCGCCCGGGTCGTTAGTAGTTCTTTCACCACAGCCCGGATCATGGAGTTCGACGAG

CGCTGCCTCAAGGTCCAGGACCCCAAATTTGATTGCCTCCTCTTTGACCTGGACGACACT

CTGTACCCGCTGAGCTCGGGGATCTCATCACACGTCAAGACTAACATTGAAGCTTACATG

GTTGAGAAGCTGGGTATTGAGGAGAGCAAGATCGAAAACCTTGGCAACCTGCTGTACAAG

AACTATGGCACCACGATGGCGGGCCTCAGGGCAATTGGCTACAACTTCGACTACGATGAG

TACCACAGCTTTGTCCATGGAAGACTGCCCTACGACAACATCAAGCCCGACCCCGTTCTC

AAGCAGATTCTCAAGAACATGCGCATGCGCAAGCTCATTTTCACCAACGGTGACATGATC

CATGCCGTGAGAGCCCTCAAGAGGCTGGGCCTGGAGGACTGCTTCGAGGGGATCATCTGC

TTCGAGACCCTGAACCCGCCGTGCCTGCTGACGCCGTGCGACCAGGCGCCGGAGATCTTC

GACATCGCCGGCCACTTCGCCGGGTTAGGCAGCGCCGACGACCTGCCCAG

>Contig429

GCCGCTTCGCTCGCTTGCCTTCTCTCGCCGTCTCCGCCTCCTCCTCCAGTCCTCCGTCCC

ACCCCTCGACGATTCCTCGGCGATAGGTGGATCCAGCGGCTGAGCGCGGCCAGGATCTCG

ACGGAGGCGCTGGAGCGGGGCCAGAGCCGCGTCATCGACGCCTCCCTCACCCTCATCCGC

GAGCGCGCCAAGCTCAAGGGCGAGCTGCTGCGTGCTATGGGCGGTGTCAAAGCTTCTGCA

ACACTCTTAGGAGTACCCCTTGGGCACAACTCGTCTTTCTTGCAGGGGCCTGCGTTTGCT

CCTCCTCGCATAAGGGAGGCCATTTGGTGTGGAAGCACCAACTCTAGCACAGAAGAAGGC

AAGGAATTAAATGATCCAAGGGTGCTAACTGATGTCGGTGATGTCCCCATACAAGAGATT

CGTGACTGTGGTGTTGAAGATGACAGATTGATGCATGTAATCAGCGAGTCTGTCAAAACA

GTAATGGACGAAGATCCTCTTCGGCCGTTGGTCTTAGGAGGCGATCACTCGATATCTTAT

CCAGGTGTTAGGGCTGTGTCTGAAAAGCTTGGC

>Contig430

GGCACGAGGGTGATACATAGACTGAGCACCAAGAAGACCTCGCAACGATGAGGTCTCTCG

GCCGCACTTCGTTGTGCTTTCACCTGGCCCTGCTTCTCCTCCTTGCCGTGGTCAGGGCAG

ATGATCCTAGCCTCGCCGATGGAGGGAGGCTCATTGACCCCAAGCCTTCCCCAAAACCCA

TTCCAAAACTGATGCCTCAACCCAACCCTAAAATGGCCCACGACTCCTGCAATGCCGGAA

CCCAACCCAATACCGGACCCAGATGCCAAGCCACAACCAAAACCAATGCCTCAGCCTGAC

CCGAAGCCAACTCCTGAAAAGCCAAAGGCTGAACCTCAGCCAGGCTCATTGCCGGTGTAT

TCACTAGACGACATTGGTTCCGACAGGAAAAGCCAAAGCCTAAACCTCAGTCAGGGTCAT

TGCCGGTGTATTCACTAGACGGTGTTTCCGACAGGACGAACCCAAAGCCCAACCCGCAAC

CTAACCCTAGGACAAACCCAGCAAAGCCGGACCCAGAAGTCAAGCCACAACCACAG

>Contig431

CGGCACGAGGCTCAGATGTCTTGGTCAAAGTCCTGTGCAAGGTGAACCACAGCGACCTGA

GGCAGCTCCTCCTGGTGTCGAAGCCAGTCAGCGAAGCAACAGTGGTTGCTAAGGAGCTGC

ATTTCGCCTTCGCGACGCCGTCGAAGGCCTCCGCGGATGGGGAGGAAGAAGACGATGGCC

CCGGGGCGCCCAAGCAACACAGGGTTGCACGGTCGCGCTGCCGGGGCATGAATTTGGCCG

GCGTCGCCGTCAATCTGTCGGAGTCGTTCAGCAGCTTGATGTCAGAGGTGTAGGTGTACA

TACCACGGCACGAAATTTCCCCGCTTGTACTTGTCCATCGGAGCGCTCTTTTTTCTTCAT

AGGCCACGTCGAACAGAAACACTTTAGTTAGTGATATGTGTAGTGATCGATCATCATATG

TGTTAGTAGTAAGTTATATATGGTCATTCTTCAATGCTGCGATTGAAGAGGACACGCTGT

TTTTTTTCTGTGTACTGTATGTAATAGTGCAAGTTATATGTAAGTGTCTGAGCGTTGGAG

CTCATCGGAAGAGTATTTAGTGTAAGTTATATGTAAGCGTCAGAGTGTTGGAACTCATTA

GAAGAGAAGTCTCTTCTCACTTTGTGTATGCTGTA

>Contig433

TAAAACTGTCATCACTCATCTTGACAAATGTTAAAAAAGCCGTTGCTTTGGGGATAACCC

GGTAAGGCCGGAGTTTTATCTCGCCACAGAGTAAATTTTGCTCATGATTGACAGCGGAGT

TTACGCTGTATCAGAAATATTATGGTGATGAACTGTTTTTTTATCCAGTATAATTTGTTG

GCATAATTAAGTACGACGAGTAAAATTACATACCTGCCCGCCCAACTCCTTCAGGTAGCG

ACTCATGAGTAAACCGTTCAAACTGAATTCCGCTTTTAAACCTTCTGGCGATCAGCCAGA

GGCGATTCGACGTCTCGAAGAGGGGCTGGAAGATGGCCTGGCGCACCAGACGTTACTTGG

CGTGACTGGCTCAGGGAAAACCTTCACCATTGCCAATGTCATTGCTGACCTTCAGCGCCC

AACCATGGTACTTGCGCCCAACAAAACGCTGGCGGCCCAGCTGTATGGCGAAATGAAAGA

GTTCTTCCCGGAAAACGCGGTGGAATATTTCGTTTCCTACTACGACTACTATCAGCCGGA

AGCCTATGTACCGAGTTCCGACACTTTCATTGAGAAAGATGCCTCGGTTAACGAACATAT

TGAGCAGATGCGTTTGTCCGCCACCAAAGCGATGCTGGAGCGGCGTGATGTGGTTGTGGT

GGCGTCTGTTTCCGCGATTTATGGTCTGGGCGATCCTGATTTATATCTCAAGATGATGCT

CCATCTCACGGTCGGTATGATTATCGATCAGCGCGCGATTCTGCGCCGACTGGCGGAGCT

GCAATACGCTCGTAATGATCAAGCATTCCAGCGTGGTACTTTCCGCGTTCGTGGCGAGGT

GATAGATATCTTCCCGGCAGAATCGGATGACATTGCACTTCGCGTGGAACTGTTTGACGA

GGAAGTGGAACGATTGTCGTTATTTGACCCGCTGACCGGGCAGATTGTTTCCACTATTCC

ACGTTTTACCATCTACCCGAAAACGCACTACGTCACACCGCGCGAGCGCATCGTACAGGC

GATGGAGGAGATCAAAGAAGAGCTGGCCGCCAGACGCAAAGTGCTGTTGGAAAACAACAA

ACTGCTGGAAGAGCAGCGGCTGACCCAGCGTACCCAGTTTGATCTGGAGATGATGAACGA

GCTGGGCTACTGTTCGGGGATGAAAACTACTCGCGCTTCCTCT

>Contig434

GAGGGTGGCTTTCGCGGCCGCGGTGGTCGAGGTGGCCAAGAGGTTTTTCGACTGGGTGGT

GTCTGGCGATTGGATGAGCTGGTGGCCGTTCTGGCGCCCCGACCGCCGCTTGCAGCGGTT

GATCGATGACGCCGACGCTCACCCCAAGGACCCTGCCAAGCAGAGCGCGCTGCTCCACGA

GCTCAACAAGTTTAGCCCAGAAGATGTCATCAAAAGAATCGAGCAAAGAAGTCATGCGGT

TGATAGCAAGGGGGTTGCAGAGTACCTTCGAGCTCTTATTCTCACAAACGCTATAGCTGA

TTACCTACCAGATGAGCGGTCTGGGCGTTCAGCAACACTACCAGCTCTGTTGCAAGAATT

GAAGCAACGTGTATCTGGGGAGGACAAGCCTTTCTCGAATCCTGGGATATCTGACAAGCA

ACCATTACATGTAGTAATGGTTGATCCTAAAGCTACTGGTAGATCAACTCGGTTTGCTCA

AGAGATTTTCTCGACTATCTTGTTCACAGTTGCTGTTGGATTTATGTGGGTAATGGGTGC

TGCTGCGCTTCAAAAGTATATTGGTAGCCTAGGTGGAATAGGCGCATCTGGTGTCGGTTC

CAGTTCATCATATTCTGCAAAAGAGTTGAA

>Contig436

CGGCACGAGGCAGCACTACAGTCGAGTTGACTAGTGCAGATTTCCTCCAGCTCCAGCTCC

GGCTCCCAGATCGAGACGGCGGCGGCGGCGGCGATGGGCGCGCACCCTTACGTGCCGGCG

GGCCTGGACCTCCCGGGCTACGTGCCGCTGCGCCTCACCCAGCTCGAGATCCTCGGGGCC

TACCTCGGCACCTCCCTCTTCGTCCTCGTCGCCGTCTGGCTCCTCTCCGGGAGATGCCGC

AGGTTGTCCGGGACCGACCGCCTGCTCATGTGCTGGTGGGCGTTCACCGGCCTCACCCAC

ATACTCATCGAGGGGCCCTTCGTCTTTACCCCCGATTTCTTCACCAAGACCAACCCCAAC

TTCTTCGACGAAGTCTGGAAGGAGTACAGCAAGGGTGACTCCAGGTACGTCGCCAGGGAC

ACCGCCACCGTCACCGTCGAGGGAATCACGGCCGTGCTGAAAGGCCCTGCCTCGCTGCTC

GCAGTCTATGCTATCGCGTCGCGAAAGTCCTACAGCCACATCCTCCAGTTTGCCGTGTGC

CTCGGTCAGCTCTACGGATGCATCGTCTACTTCACCACCGCCTACTTGGACGGCTTCAAC

TTCTGGGCCAGCCCGGTCTACTTCTGGGCATATTTCATCGGCGC

>Contig437

CGGCACGAGGATCAACTGCTCCAAGGATGTCAAAATTCAAGGCATCATTGGACCTTGTAC

TTCCCTGGAGAAGAAAGGTCCTCTATCCTCAGATACAGTTATAGGTCAGGGGAACACTAG

CGCTTGGAAGATGTGTGGTCTAGATAGGAAAACATCACTATGCATAGTATTTGATATGGC

CAAAAAAGATGCCCCGGACGCAATTGGTCAATCACAAAATAATCTGTTCTACTTCCAATT

CTTAACCTATTATCAGCATCATGACGGGCAAATGAGATTGCGATCGACTACAATTTCAAG

AAGATGGGTTGCTGGTTCTGGCAGTGTGCAGGAGTTAATAACTGGCTTTGACCAAGAGGC

AGCAGCAGCAGTCATGGCACGCTTGGTCTCATTTAAGATGGAAGCTGAGGTTGATTTTGA

TCCAGTAAGATGGCTTGACCGAGCATTGATTAGTTTATGTTCGAAATTTGGAGACTATCA

GAAGGAAGCACCTTCATCTTTCAGTTTGTCCCCGCGTCTATCAATTTTTCCACAGTTCAT

TTTTAATTTGAGACGTTCCCAGTTTATTCAGGTTTTCAACAATAGTCCAGATGAAACTGC

ATATTTCAGGATGATGTTAAACAGAGAGAATGTAGCGAATGCAGTTGTGATGATCCAGCC

TTCACTTATATCATATTCATTTCAGTCAGGTCCCGAGCCTGTTCTATTGGATGTAAGCGC

AATTGCTGGTGACAGGATACTTTTGTTGGATTCTTATTTTACTGTTGTCATATTCCATGG

GATAACGATCGCACAGTGGCGAAAGGCTGGTTACCAACATCAAGAAGGCCACGAGGTGTT

TGCCCAGTTGTTGCAAGCCCCACAGGAGGAAGCTGATTCAATAATCAAGGAGCGGTTTCC

TGTGCCTCGTTTGGTTGTGTGTGATCAATATGGTTCTCAGGCTCGATTCTTGCTGGCAAA

GCTGAACCCATCCGTGACATATGACTCCGACACTCCTCCGCCTCCTGGGGGGGATATGAT

ATTCACAGATGATGCGAGCTTCCAGGTCTTCATGGAGCATCTCCAGCGGTTGGCAGTTCA

GTAGAGCATGGACGTGCAGTAATTTTGCACCAATGGCCTCTTCTCTCATGTACAGCAAAG

AAAACCTTTTCGTGTGTGTGTATCACTGTTATACCGGTTGCCACGTACTGTAGGCCGACA

TACCGCTGTTAGCTCCGCATCTGTACATTGCCATGGACTGTAAGGTTTATGTCGTATATC

CCGAGATGGAGCATTCATAAATTGTGCTCTTAGCTCAACATATGGTTGATCATTGCTGAC

ACCAAAGAAGATTGTCATCGTCTGGGTGTTCAAGCTTACTGGTGAAGAGGATTTTTGGAG

TCCCATGTGTTACTAGCGATGCGTCTAGATTTATTGATAGCAGGTGTTTGCTTAAAAAAT

GTGTGACAATTGATGTTTTATGGTGG

>Contig439

CGGCACGAGGCAAAGACTAGAACAGAAAGAGATCTCCAAGCACGCGACGATATGTCAACG

CCGGCGATGAAGATCGGCCCGTATGGCGGCCCAGGAGGTAATCACTGGGACATTAATGCT

CTCAATCCGCCCAAAAGGCTTGTGAGCATCCAGATTTGGAGCACTGATTCCTCCTGCGAA

GGGGCCGGCGGGGTCGTCAACGGCATCTCGTTCAGCTATCTTGACATGGCAGGGCAACTA

ATCCCCTCGGGTCCATGGGGCTCCCAGACCGGTAAACCCCACGTGATTAACATTGGTGAA

GACGAGCGCCTGATCACTGTCTACGGCACCAGCGATGGCAAGTTTGTCACCTCGCTTAAA

TTTGTCACTGACCGGGCTTCTGATCCGTATGGGCCATACGGGGCACCGTCTACCATAAGT

ACTTTCAGCTTCCAGGGCGGCTCCATCCTCGCCTTCTTGGGCCGCTCCTCAGAGAAACTC

AATGCCATCGGCGCCTACAAACTTGGGGTTTGATCCATCTATCCATCATGGCCAGCATAC

GTGTCTGCTGACTTCCGTATGTTTGGCTTCGGTGGATACATGTGTTCGACCTATATCTAC

TGCTTGTATTTGCAGCTAAATAAAATGGGGATCCCTCGCTGCTGCATCCTATGGATGGAT

GGATCCCTGCCGTCCGTGCGTGTGTGCGCGTGCTCGCTCTTTGTTTCCATATATATTGTA

TCGTCCTAGCCTGTGTAACTATAAGATGAATAAAAGTCTCAGAAACTAAGTACTCCCTCC

ATAAAGAAATATAAGAGTAGTGATCTAGACGGTAAAATATTCTTCGCGGAGGGAGGTTT

>Contig443

CGAGGGAAGCCCGGCACCTCCGGCCTCCGCAAGAAGGTTACAGTATTCCAGCAGCCTCAT

TACCTCGCGAATTTCGTTCAATCAACATTTAATGCCCTTCCAGCTGACCAAGTAAAAGGT

GCCACCATTGTTGTCTCCGGCGATGGGCGCTATTTCTCAAAGGATGCTGTTCAGATCATT

GCAAAAATGGCTGCTGCTAATGGAGTAAGACGTGTCTGGGTTGGACAAGACAGTCTCTTG

TCAACTCCAGCTGTATCTGCTATCATCCGTGAAAGAATTTCTGCAGATGGCGCAAAGGCT

ACTGGTGCCTTCATTTTAACAGCCAGCCACAACCCAGGTGGTCCAACCGAGGACTTTGGA

ATCAAATACAACATGGGGAATGGTGGACCCGCCCCTGAGTCTGTTACCGATAAGATCTTC

TCTAATACAAAGACAATTACTGAATACCTCATTGCGGAGGACCTTCCAAATGTTGATATT

TCTGTGATAGGTGTCACTTCCTTCACTGGACCTGAAGGCCCTTTGGATGTCGATGTCTTT

GACTCTGCTACAGATTACATCAAGCTAATGAAGACAATCTTTGACTTCGAGTCTATTAAA

AAGCTTCTGGCATCTCCAAAGTTCTCGTTCTGTTTTGATGGCCTTCACGGTGTTGCTGGA

GCTTATGCAAAGCGCATGTTTGTGGATGAGCTCGGCGCCAGTGAAAGCTCACTGTTGAAC

TGTGTTCCAAAGGAAGACTTTGGAGGTGGTCATCCAGACCCTAACCTCACCTACGCAAAA

GAACTGGTTGATCGGATGGGTCTTGGGAAAACCTCAAATGTTGAACCCCCTGAATTTGGT

GCCGCAGCTGATGGGGATGCTGACCGCAACAT

>Contig444

GGCACGAGGTCACCGCCAGACGCCGTTTCAATCATGGCACGCCGAGGATTAATGGAGCAG

GACCTAACCAAGCTTGATGTCACCAAGCTCCACCCGTTGTCACCCGAAGTCATCTCGCGC

CAAGCGACGATCAACATTGGTACAATTGGTCATGTGGCCCATGGAAAGTCTACTGTTGTG

AAAGCTATATCTGGAGTTCAGACTGTTCGCTTCAAGAATGAGCTTGAACGTAACATCACT

ATCAAGCTGGGCTATGCTAATGCAAAAATCTACAAATGCGAGGATGACAGATGTCCACGA

CCAATGTGTTACAAGGCTTATGGAAGCGGAAAAGAAGATACTCCTGCCTGTGATGTTCCT

GGGTTTGAAAACACTAGGATGAAGCTCCTGAGACATGTTTCCTTTGTTGATTGCCCGGGC

CACGACATTCTCATGGCTACAATGCTTAATGGAGCAGCTATCATGGATGGAGCCTTGCTT

TTGATAGCAGCAAATGAAAGCTGCCCACAACCCCAGACATCTGAGCATCTTGCAGCTGTT

GAGATCATGCGTCTCCAACATCTCATAATTTTGCAGAATAAGATCGATCTTATCCAGGAA

AGCGCAGCAATGAACCAGCATGAAGCAATCCAAAAATTTATCCAGGGCACGATAGCTGAA

GGTGCTCCTGTTGTGCCAATATCTGCCCAGCTGAAGTACAACATTGATGTGATCTGTGAA

TATATTATTAAGAAAATCCCCATTCCGGAAAGGAACTTCACTTCACCTCCCAACATGATT

GTTATTCGCTCCTTTGATGTCAACAAGCCTGGTTCAGAGGTTGATGAAATCAGGGGTGGG

GTAGCAGGTGGCA

>Contig445

GGCACGAGGACCGCAGCACGCACCACGCGCCCCCCCCCCCCACCCTGTGATCCCTCGCCG

TGGTCTCGCCGCCGCGCCGACCCCTCCACCGGCATTCGATTCGGAGCCCAGCTCGTCCGC

CGGCGCGGAGCCATGGGCACGGTTGTGGATGCTCCCGCAGTTGTGACTGAGAAGGAGGTG

GTTGCTGAGAACATGTTGGGTGACAAGAAAGTCACAGTAGTATTTGTTCTTGGCGGTCCT

GGAAGTGGAAAGGGCACACAGTGTTCCAACATTGTTGAACACTTTGGATTCACCCATCTT

AGTGCTGGAGATCTTTTGCGCGCGGAGATTAAATCTGGCTCTGAGAATGGAACTATGATT

GAGAACATGATAAAGGAGGGAAAGATTGTTCCGTCGGAGGTGACTATAAAGCTCTTGCAG

CAAGCCATGATAAAGAATGAGAATGATAAGTTCCTCATCGACGGGTTTCCAAGGAATGAA

GAGAATCGTGCTGCGTTCGAGAATGTTACAAAAATTTCGCCTGCATTTGTGCTATTCTTC

AATTGTTCCGAGGAAGAGATGGAAAGACGTCTTCTGGGACGCAATGAGGGTAGAGTTGAT

GACAACATCGAGACTATCAGGAAGAGATTCAAAGTTTTTGTTGAATCCAGTTTGCCTGTG

ATTGAGTACTATGACGCGAAGGACAAGGT

>Contig446

AGGCTTTCTCTTTCTGTCCCGCAAACCCCCGAGAGATCACAGCATGTTCGGCTTCGGTCA

CCACGGCCACCATGGCCAGAACCCCCCGGCCCACGCTCCGGCCGCCGCCGGAGGCAACCA

GCCCACCTTCAAGATCTTCTGCAAGGCCGACGAGGGCTACTGCCTCTCCGTCCGTGACGG

CAACGTCGTCCTCGCCCCGTCCAACCCCCGTGACGAGCACCAGCACTGGTTCAAGGACAT

GCGCTTCAGCGCCCAGATCAAGGACGAGGAGGGTAACCCCGCCTTCGCCATCGTCAACAA

GGCCACTGGCCTCGCCGTCAAGCACTCGCTCGGCCAGTCCCACCCGGTGAAGCTCGTGCC

ATTCAACCCGGAGTACCTCGACGAGTCCGTGATGTGGACGGAGAGCGGCGACGTCGGCAA

GGGCTTCCGCTGCATTCGCATGGTGAACAACATCCGCCTCAACTTCGACGCCCTCAACGG

TGACAAGGACCACGGCGGCGTGCACGACGGCACCACCGTCGTTCTCTGGGAGTGGGCCAA

GGGCGACAACCAGAGCTGGAAGATCCTCCCCTGGGGCGAAGAGGCCTACGCCGGAGGCAG

CGCCAACGCCCCCCGCGGCGGCTCCTCAGAGCCCACCGTGCGCATCTTCTGCAAGGCCGA

CGACGGCTTCAGCGCCACCGTCCGCAACGGCACTGTGGTCCTCGCGCCCACCAACCCCCG

CGACGAGTACCAGCACTGGTTCAAGGACATGAGGCACAGC

>Contig447

CTGCCTCAAATTATAAATGGACATCTGCTGAACTTTGAACAAAACGAAGCAAGGTTGATA

GCGCGACAAGGCATATCCGTCCTGTATTCAGGCTAATTATTGATGCTCAGATCCACTCAC

AGGAGAATGCAAAAGGCTGTTTTTAGATTAGAAGTGGCCTTCATTGGAACAGAAAAATAA

AAAAAATGAGGGCATAAAGTTTCATCATCATCCCTCAAGAGCAGAGGCACCCGATATGAT

CTCAGTGAGCTCAGTAGTGATAGATGCTTGACGTGTCCTGTTGTATGTCAGTGTAAGACG

GTCAAGCATTTCACCGGCATTTCTGCTGGAGCTGTCCATAGCAGACATACGGGCACCAAG

CTCACTGCACGCATTCTCTAGAACAGCATTATACATAACACAAGAAAACTGGAACTCGGT

AAGATTCTGCAGGATTTCTGATTTTGTTTCACCTCCTTCAATTTCATACGAATCCAGCTG

ACCAACCTTCCCGCCAGATTCTGATTCTTTCTCCATAACCTCTGGGGACAGTATTGTGGC

CAGTGTAGGCCTAAATGAAATGACAGACTGAAACTTGTTAAAAACAACTCTTATAGCATC

ATATTCAACATTTTTCAAGATATCATCTGCCAGCACAGCAACCTGTGTGTAATTGATAGG

GTTTTTCTGCAACTCAGATACTGTCATTTCAATATGCTTCCTGGAGTCACGGATGAGTTG

AACTTTCCCCTTTTCTCCTAATATGACATACTTGCTTTCTTTCTCTGGACCAGAAGTCAT

TTTCTGGAGTGCTTTGCTGACTTTAACAGATGTAGAATTGATACCACCACAGAGACCCTT

ATCAGATGTAATGGCTACAATAACATTCTTCTTGACATTTACACTAGGAGCATCCCCAAG

AAGAGCTGTGAATGGCTGCCATAGACCACGTGAATTCTCAGTTCTTGTTTGAACAGCACG

AAGCTTTGATGCTGCAACCATCTTCATTGCTTTCGTAATCTTTTGGATGTTCTTGACACT

TTTCATGCGGTTCCTCACAATTTGTGTTGAGATCGACCGTGCACCAAGCGGAGCAATCTG

CGACTGGGAGGCAGCCCGGGAGAGCGCAGCCATGGTGGCTGCCGGGGAGGGGGTGTTGAG

GAGGACACGCCGCCCCTCGCGTCTTAGGGCCGCCATCGCCATCGCCATCGCCGGTGGAG

>Contig448

AGGTTTCCGCCGCGATCTCGTCTCGATCCGCACTCTCTATCTGAGATTTGGAATCGGGTG

AAGCCTGCAGCTCCTGCCGACATGGCGCTCGTTTTGCATTCAGGCAGCGGCAACAAGAAT

GCCTTCAAGGCACTTATTGCTGCAGAGTACTGTGGGGTCAAGGTTGAGCTCCCCAAGAAC

TTCGAGATGGGTGTATCAAACAAAACCCCTGAATTCATCAAGATGAACCCCCTTGGGAAG

GTTCCTGTTCTTGAGACTCCTGATGGTGCTGTTTTTGAGAGCAATGCTATTGCACGCTAT

GTTGCTCGCTCAAAGGGTGACAACCTGCTTTGGGGTGGTTCTCTTATTGAATATGCACGT

GTTGAGCAATGGATGGACTTTGCTGCCACAGAGGTTGATCCCAATATCGCAAGGTGGTTG

TACCCAAGGCTTGCTTATAGGGCTTTCAATGCCCAGGATGAGGAATTCGGCATTGCTGGA

TTAAAGAGGGCCCTTGAAGCATTGAACACACACCTGGCATCAAACACATTCCTTGTTGGG

CATTCTGTCACTCTGGCTGATATTGTCATGACATGCAACCTCTACCATGGTTTTGCTCGG

ATCTTGACCAAGACTTTCACATCTGAGTTCCCTCATGTTGAGAGGTACTTCTGGACCATG

GTTAACCAGCCTAACTTCAAGAAGGTCATTGGCGAATTCAAGCAGGCAGAGGTTGTACCT

CCTGTTCAGAAGAAGGCTGCTCCTGCTAAACAAAAGGAGGCCAAGAAAGAGGCTCCCAAG

GCGGCCCCAAAGCCAGCAGTAGTTGAGGCACCAGAGGAAGAGGCACCAAAGCCTAAGCCC

AAAAATCCCCTTGATCTACTGCCACCAAGCAAGATGGTACTTGATGACTGGAAGAGGCTA

TACTCAAACACTAAGAGCAACTTCCATGATGTTGCTGTTAAAGGTTTCTGGGAGATGTAT

GACCCAGAGGGCTACTCTCTGTGGTTCTGTGAC

>Contig449

CTGCTCATCCACGAGGGCGTCAAGGCCGAGGAGGAGTTCGAGAAATCCGGCAAGGTTCCC

GACCCGGAGTCCACCGACAACCCCGAGTTCAAGATCGTCCTCACCATCATCCGCGACGGG

CTCAAGACCGACGCCAGCAAGTACCGCAAGATGAAGGAGAGGCTCGTCGGTGTCTCCGAG

GAGACCACCACCGGCGTCAAGAGGCTCTACCAGATGCAGGAGTCCGGCACCCTCCTCTTC

CCCGCCATCAACGTCAACGACTCCGTCACCAAGAGCAAGTTTGACAACCTTTACGGTTGC

CGTCACTCGCTCCCTGATGGTCTTATGAGGGCCACTGATGTTATGATCGCCGGCAAGGTC

GCCGTGGTCTGCGGTTACGGTGATGTTGGCAAGGGCTGTGCCGCCGCACTCAAGCAGGCT

GGTGCCCGTGTGATCGTGACAGAGATTGACCCCATCTGTGCCCTTCAGGCCCTGATGGAG

GGTATCCAGATCCTCACCTTGGAGGATGTTGTCTCTGAGGCTGACATCTTTGTGACCACC

ACCGGAAACAAGGACATCATCATGGTCGACCACATGAGGAAGATGAAGAACAACGCCATT

GTCTGCAACATTGGTCACTTTGACAATGAGATCGACATGAACGGCCTTGAGACCTACCCT

GGTGTCAAGCGCATCACCATCAAGCCCCAGACTGACCGTTGGGTCTTCCCCGAGACCAAG

ACTGGCATCATTGTTCTTGCTGAGGGTCGTCTGATGAACCTTGGATGTGCCACTGGCCAC

CCCAGCTTCGTCATGTCCTGCTCATTCACCAACCAGGTTATTGCTCAGCTTGAGTTGTGG

AACGAGAAGGCCACTGGCAAGTACGAGAAGAAGGTGTACGTTCTCCCCAAGCACCTGGAC

GAGAAGGTCGCGGCCCTCCACTTGGGCAAGCTCGGCGCCAGGCTGACCAAGCTCACCAAG

TCCCAGTCTGACTACATTAGCATCCCAATTGAGGGTCCTTACAAGCCTGCGGCTTACCGG

TACTAGTGTGTCCAGCATGACTAGCGGCTGGCCTGAGCCTGAGTCGGAGCAGCGGCACCA

ACGGGAACTCTATCAACTATCCTGTTTCCCTTCTATTATCTTACATGCTG

>Contig450

GTTGTCGAGAGGAACTACGCATCCATCCCTTCCGCCCCCAGCTACCTCGCTTGCTCCACG

CGTCGTCTTTGAAGGAGAGGAAGATGACGAGCGTATGGAAGACCAAGGTTCTCCCCGGGC

TCAACAAGATCTTCGACAAGGATGGCAAGAAGGCCGCCGCCGCCGAGTTCTTGAAATCCT

TCAACAAGGAGGAGATTGACAAGGAGATTGAGGACAAGAAGACAGAACTAGAGCCCAAGG

TTGTGGAGACCATTGAAGCATCTCCTCCAGAAATTAAGGGCTTGATAAAGGATAAGAAAA

CATCCAAGATCAAGAAGAACTCAGTTGCCGTCACCAAGTTCCTTGACGATTTGGCCAAGA

TTGATTTCCCCGGAGCCAAGCTGGTGAGCGACGCGGTGGCCAAGTCCGGCACCACGCCCC

TCTCCCCGGCCATCGTCTTCATCTTGGACAAGGTTGCGCCTTTCGTCCCCGCGCCCAAAG

AGGAGCCCAAGGCCGAGCCCGAGGCCGCCGCCCCGGCAGAGGAGACCACCACCCGCGAGG

TCGCCGTGGAGGAGAAGGAGGAGGAGGCCGAGCCCTCCGCCGCACCGGCAGAAGCCGCGC

CGGTGGAGGCAGCCGCGCCTGCTGCTGAGGTAGTGGAGGAGAAGAAAGAGGAGGAGAAAC

CCGCCGAAGCCGCCGCACCTGCCGCCGAGGAGCCAGAGAAGAAGTGATCGGTCGCCGCCG

GCCGGAGTCCAAGAGCAACCCAAACCACCACCTGGCTTGCTGCTGGTAATGAAAACATTT

TAAGTTATTGTGAATTTGTGGTCGCTCTCTGTGTCAAGGTAGCTGCACAAAAGTAAGCAC

GTACGTACGTACACTGCACTTGCCATGCATACCACGCATGCAAACGTACACGCATGGAAC

ATTTGTTATTCTTTCGATGGACCGGTCTCCTCACCGTATGTGTACATTTTACGTGGGGCT

TTCTTGCTTTTTTGTGGTGGTTAAAACCTGTGTAATTATGATTATATTGGTGTGTTAATT

TGTTGGTCATGGTATAAGCAGGAACTTGTGC

>Contig453

AAGGAGACGGGGACAGCACAGCCTCAGCGCACGGAAAGAGAGGGGGGAGAGATTCGCACG

TCGCCGAGATGCTGGCCGTGTTCGACCAGACGGTGGCCAAGTGCCCGGAGGGCCTCCGCA

TGATGAAGGGCTTAACAAGAACCCCCTCGTCCCCAGGATGTTCGGTTCTGTAAATGACAT

ATTTTGCCTGTTCCAAGGACATGTTGAGAACATTGGCAACCTGAAGCAACACTATGGTCT

GAGCAAGACAGCAAATGAGGTTACCATCCTGATTGAGGCCTACAGAACCCTCCGGGACAG

GGGCCCACTCCCAGCTAGCCAGGTCGTGAGAGATCTCAGTGGAAGGTTCGCTTTCATCCT

CTACGATACCGTGTCGAAATCCACCTTCGTCGCTGCTGATGCTGATGGCAGTATCCCCTT

CTTCTGGGGAGTTGAC

>Contig455

TCCTTTCGGTTCTTCGGCCTCCTGAAGAATAGCATCAGCACAACTGCACAAGAGCATCGC

TAGCACGGAAGAAATGGCGGCTGAGACGTTCCTCTTCACGTCCGAGTCCGTGAACGAGGG

CCATCCCGACAAGCTGTGCGACCAGGTCTCTGACGCCGTCTTGGACGCCTGCCTGGCCCA

GGATCCCGACAGCAAGGTTGCCTGCGAGACCTGCACCAAGACCAACATGGTCATGGTCTT

TGGCGAGATCACCACCAAGGCCACCGTCGACTACGAGAAGATCGTCCGCGACACCTGCCG

CAACATCGGCTTCATCTCTGACGACGTCGGTCTCGATGCTGACCATTGCAAGGTGCTTGT

CAACATCGAGCAGCAATCCCCTGACATTGCCCAGGGTGTTCACGGACACTTCACCAAGCG

CCCCGAAGAGATCGGTGCCGGTGACCAGGGCATCATGTTCGGCTACGCCACCGATGAGAC

TCCTGAGCTGATGCCCCTCACCCACATGCTCGCCACCAAGCTTGGAGCTCGCCTCACCGA

GGTCCGCAAGAATGGCACCTGCGCCTGGCTCAGGCCTGACGGAAAGACCCAGGTCACCAT

CGAGTACCTAAACGAGGATGGTGCCATGGTACCTGTTCGTGTGCACACCGTCCTCATCTC

CACCCAGCACGACGAGACCGTCACCAACGACGAGATTGCCGCGGACCTCAAGGAGCATGT

CATCAAGCCGGTGATCCCCGCGAAGTACCTCGATGAGAACACCATCTTCCACCTGAACCC

GTCTGGCCGCTTCGTCATCGGCGGCCCTCACGGTGACGCCGGTCTCACCGGCCGCAAGAT

CATCATCGACACCTATGGTGGCTGGGGAGCCCACGGCGGCGGTGCCTTCTCTGGCAAGGA

CCCAACCAAGGTCGACCGCAGTGGCGCCTACATTGCCAGGCAGGCCGCCAAGAGCATCAT

CGCCAGCGGCCTCGCACGCCGCTGCATTGTGCAGATCTCATACGCCATCGGTGTGCCTGA

GCCTTTGTCTGTGTTCGTCGACTCCTACGGCACCGGCAAGATCCCCGACAGGGAGATCCT

CAAGCTCGTGAAGGAGAACTTTGACTTCAGGCCCGGGATGATCAGCATCAACCTGGACTT

GAAGAAAGGTGGAAACAGGTTCATCAAGACCGCTGCTTACGGTCACTTTGGCCGCGATGA

TGCCGAC

>Contig457

CATTGCAAAGGGCTCTGTTCATGAACCCGACCATCCTTTTGCTTGATGAGCCAACCAACC

ATCTTGATCTTGAGGCTTGTGTCTGGCTGGAAGAGAAATTAAAGAACTTTGAGCGTATAC

TTGTTGCTATCTCGCATTCCCAAGATTTTCTAAATGGAGTGTGCACTAACATCATCCACA

TGCAGAACAAGACCCTCAAGTTATATACTGGAACATTATGACCACGATGTTCAAACTCGC

TCTGAGCTTGAAGAGAATCAAATGAAGCAGTACAAATGGGAACAGGAACAGATAGCTAAT

ATGAAGGAGTACATTGCACGATTTGGTCATGGATCTGCGAAGCTTGCTCGTCAGGCTCAG

AGCAAGGAGAAGACTCTTGCAAAGATGGAGCGTGGTGGTCTCGCTGAGAAGGTTGTCAAT

GACAGGATTCTTGTTTTCCGCTTTACAGATGTTGGCAAACTCCCACCACCAGTGCTGCAG

TTTGCTGATGTCACATTTGGTTACACTCCGGATAATCTCATCTACAAGAACCTTGACTTC

GGTGTTGACCTTGACTCGAGAGTTGCACTGGTCGGTCCCAATGGGGCAGGTAAGAGCACA

CTTCTGAAGCTCATGACAGGTGACCTATCTCCGTTGGATGGCATGGTCAGACGCCACAAC

CACCTACGCATTGCACAATTCCATCAACATCTCACTGAGAAGCTGGACCTGGACATGCCG

GCCCTGCAGTACATGATGAGGGAGTACCCTGGGAATGAAGAGGAGAAGATGAGAGCTGCG

ATTGGCAAGTTTGGCCTGTCAGGAAAGGCACAGGTGATGCCAATGAAAAACCTGTCTGAT

GGGCAGAAGGCCCGTGTCATTTTTGCTTGGCTAGCATTTAGGCAGCCACAGATGCTGTTG

CTTGATGAGCCGACAAATCATCTTGACATTGAGACCATTGACTCACTTGCTGAGGCACTG

AAGGAATGGG

>Contig458

GCACGAGGCTTCGTGTTCCCCCCTCCCCTTCTCGCCTTCTCCGCCCATAATCTTTCTCGG

TCTAAGAAGGATTCCACGGGACAAGGCACAGCGGGAGGATCTCGAAGGGAGAATACGGGG

GAGAAAAATGTTGCTTGGAGCTAAGTGATACCGTCGTATAACGCTAACACTTGTGCGTAA

AATGGAGGATCATCCTGGCCATCCTATTTCCAACTATGATTTCTTGTCAGGGAATGGTTG

TCATACGAAGAAATTAGTTCATAAGAACTACGACCAGGACTCCTCATCAGCCAAGTCTGG

CCGGTCACAACAAGAAGCATCTGCAACGAGTGACAGTAATCTAAATGAGCAACACACCTC

AAGACCCCCATCACAATCTGACAATGACAATGATCATGGGAAGCCCGACCAGCACATGAT

AAAGCCGCTTTTATCTTTGGGGAACCCAGAGACTGTTGCTCCCCCACCAATGATTGATTG

TAGCCAATCATTTGCATATATTCCTTATACTGCTGATGCTTATGCTGGGATCTTTCCAGG

ATATGCCTCACACGCTATTGTTCATCCCCAATTAAATGCTGCAACAAACTCTCGTGTGCC

GCTCCCTATTGAGCCTGCAGCAGAAGAGCCAATGTTTGTTAATGCAAAGCAATACCATGC

AATTCTTAGGAGGAGGCAGATACGTGCTAAACTGGAGGCCCAAAATAAGCTCGTGAAAGC

CCGGAAGCCATACCTTCATGAATCTCGGCACCGCCATGCCATGAAGCGAGCTCGTGGAAC

AGGAGGGCGGTTCCTCAACACAAAACAACTCGAGGAGCAGAAGCAGAAGCAGGCTTCAGG

TGGTGCAAGCTGTACAAAGGTCCTTGGCAAGAATACACTCCTTCAGGATAGCCCTGCCTT

CGCACCTTCGGCATCAGCTCCGGCCAACGTGTCAAGCTTTTCAACAATCAGCATGCTGGC

TAATCAGGAGCGCACCTGCTTCCCCTCGGTTGGCTTCCGTCCCACAGTTAGCTTCAGTGC

ACTGAATGGCAACGGGAAGCTGGCAACGAACGGAATGCACCAGCGGGCTTCCATGATGAG

GTAAAGCAAAGCATCCTCTGGTGCGCTGCCGGTGGCAATTCAT

>Contig459

CTCCCCAGTAGCTAGCCTAGTCTAGGGTTTCCGTAGTTCGGAGAGGAGAGAAAGGGAGAG

ATCCATGGCGGAGACGGAGTACCGCTGCTTCGTGGGCGGCCTCGCCTGGGCCACCGACGA

CAACAACCTCCAGCAGGCCTTCAGCCAGTACGGCGAGATCCTCGACGCCAAGATCATCAA

CGACCGCGAGACGGGGAGGTCCCGTGGGTTCGGCTTCGTCACGTTCGGCAGCGAGGAGTC

GATGCGCCAGGCCATCGAGGAGATGAACGGCAAGGAGCTCGACGGGCGCAACATCACCGT

CAACGAGGCCCAGTCCCGCCGCTCGGGCGGAGGGTACGGCGGCCAGCGCGGCGGTGGCGG

CGGCGACTCCGGCGGCCAGTGGAGGAACTGAGCCTCGAATCTCCCAGTTATCGATCTATC

TACCGTGTCGTCGTCGTCGTCTTCGTTAGCAAGTTATCTTCGGTGTCGTCGTGTTCCTGT

GT

>Contig460

CGGCACGAGGCTTCCTTCTACTTCGAGCGTCCTCTGCGACACTGCAGTGACTGCCTCCGT

GTGCAGGTAGTGTCTGAGAGCGGGGATGGCGACGCACTTCGTGCTCAACACCGGCGCTAA

GATCCCCTCCGTGGGGCTCGGCACCTGGCAGTCCGACCCCGGCGTCGTCGGCGAGGCCGT

CTACGCCGCCGTCAAGGCCGGGTACCGCCACATCGACTGCGCCAGGGCCTACAACAACGA

GAAGGAGGTGGGTTTGGCACTGAAGAAGCTATTTGAAGAGGGTGTAGTCAAGCGTGAGGA

TCTGTTTATCACCTCTAAGCTATGTTGTGGTCATCATGCCCCAGAAGATGTGCCTGAGGC

ACTTGGGGATTCCCTCAATGACTTGCAGCTTGACTACTTGGATCTTTACCTTATCCACTG

GCCATTTAAAATCAAGAAGGGAACAAGCATTGGCAACCCTGAAAACTTCTTACCACCTGA

CATCCCAGCTACATGGGGAGCAATGGAGAAGTTGCATGATGCTGGCAAAGCTCGTGCAAT

TGGTGTGAGTAATTTCTCATCGAAGAAATTGGGTGACTTGCTTGCTGTAGCCCGCGTACC

TCCGGCTGTTGACCAGGTGGAGTGCCATCCTACTTGGCAGCAATCTAAACTCCATACCTT

CTGTCAGTCAACCGGTGTTCACCTCTCTGCGTACTCACCACTAGGTTCACCTGGCTCAAC

ATGGATGAATGGTAATGTCCTCAAAGAACCCGTCGTCCTCTCAATTGCAGAGAAGCTTGG

CAAAACTCCAGCACAAGTGGCGTTGCGCTGGAACATTCAGATGGGTCACAGTGTACTGCC

AAAGAGCGTGAGTGAAGAACGGATAAAGCAGAACCTTGCTGTTTATGACTGGTCTATTCC

AGAAGACTTGCTTGCAAAGTTCTCTGAGATTAAGCAGGCCAGGCTGCTCATGGGCAACTT

CATCGTCAACAAAGACAGCGTTTACAAGACCCACGATGAGCTCTGGGACGGCGAAATCTA

GGACAGCTCCGTAGTCCCGTCGTGGTTGGGAGC

>Contig461

GCACGAGGTCTCTCTTTCCCCCCTCCGCCTCCGCCTCCTCTCGAACTCGTCGAGCGCCGC

GACCGACGCACGCGCGCGCGGCGGCGAGATGAAGGTCTCCGTGAAGACGCTCAAGGGCTC

CAAATTCGAGATCGAAGTGAACCCCGCCGACAAGGTTTCTGATGTAAAGAAGCTCATTGA

GACTTCACAAGGGCAGAATGTGTACCCAGCTGATCAACAAATGCTCATATACCAAGGGAC

AGTTCTTAAGGACGAGACTACGCTGGAGGAAAACAAAGTTGTTGAAAACAACTTTCTTGT

GATAATGCTTAGACAGAATAAGGGCTCATCAAGTGCAGCTCCAGCTAAATCCAAGGAACC

CTCAAATCAGGCACCCCCTACTCAGACAGTGCCTGCTACTCCTGCCTCTCAAGCACCAGC

CACACCAGCACCTCAAGCAGTGGCTGCGCAAGCACCTATTGTACCTGTCAGTGCTCCTGG

TCCAGCTGCCACCGCCTCCCCAGCTCCTGCTGTTGCTGTCTCCACTGAAGCAGAAACTTA

TGGTCAGGCTGCTTCAAACCTTGTCGCGGGAGGCACCCTAGAGGCAACAATTCAGTCAAT

TCTTGAAATGGGTGGTGGAACATGGGACAGAGACACTGTGCTGCGTGCCCTACGTGCTGC

ATTCAACAACCCGGAGCGGGCTGTTGAGTATTTATATTCTGGTATTCCTGAGCCGATGGA

GATTCCTGCACCACCACCAAGTGCCCAGCCAGCTGATCCTGCCCTGGCTTCACAGCAGCT

CAACCTGCA

>Contig463

GCACGAGGGTGAGTCATTAATTAAGGCGAAGGAATGGCTTGTGGATCTTGTTGAGATAAG

TTTGGAAGCCGGCAAGATGAGTGAAGCGGAACTGACTCAATCAGCCTCTAACAAAGGTGG

TCCGGTTACTCCAGTCTCACGGACTAATCTTGCTACCTTCAAAGTGGTTACTAATCGTAC

ATTATTCCGCAAGGCAAAAGAATGGATGGTATATTTTACAGAGGAATGTTTGAAAGCTTG

GCCAGACGCGAGTCAAGATGATCTTCCTCAACTTTTCTGCAAGGATGGTGATCCTGATGG

CACAGTCCCCCGTACAGTGCCTGGTAGCTTGACTAGTCTTGCTGAAAATTCAGCGATTGC

ATCAGTCAAAAAGCCAGATGCTCTATTCTCGCAAACCATGCTTGGTACCTTGACAAATCC

TGCTGAAGACTCTGGGATGTCATCAGTGACAAATGCAGGTGCATATAAACCTTTGAGTGA

AGAATACTTTTCCAGTTCAAGGAAGAATCCAAGGATGGAACTGGCAACTATTAGGCAAGA

TTTTGATCCAAAAAGGCGACGGACAACTCTGACTTCGCAGATTGAAACCGGAACTGAAGA

TCAGCAAGCAACATGAGTGTCTAACTGAATGGTAGCATCGACTGTACCAGCTGCTGCTTT

TGCCTTTGCGAAGACGGTCAATTAGCTACCTACTGGTGTTACTTGACGTTAAGCTACTTG

TTAGGGGCTTGCGAGTCTT

>Contig464

GCACGAGGCTACACTGTTAAAGGAGGTTAATGTTAAGAACTTGTTTGCTGTCTTGCTCGG

TAATAAAACCGCTGTGAATGGTGGGAGCGGCAAGGTCTTGGACAGTGGCCCTAATGATCA

CATTTTTGTGTTTTACAGTGACCATGGGGGTCCTGGGGTCATTGGGATGCCCACCAATCC

ATACGTTTACGGTGACGATCTTGTAGATGTCCTGAAGAAAAAGCATGCTGCTGGAAGCTA

CAAAAGCCTGGTATTTTACCTTGAAGCCTGTGAAGCCGGGAGTGTCTTCGAGGGGCTTCT

GCCGAATGACATCGGTGTCTACGCGACCACCGCGTCGGACGCAGAGGAGAGCAGTTGGGG

AACGTATTGCCCCGGCGAGTACCCCAGCCCTCCGCCCGAATATGACACCTGCTTGGGCGA

CCTGTACAGCATTTCTTGGATGGAAGACAGTGATGTACACAACCTGAGAACTGAATCTCT

CAAGCAGCAATATGACTTGGTCAAGAAGAGAACAGCAGCTCAGGACTCATACAGCTATGG

TTCCCATGTGATGCAATACGGTTCTTTGGACCTGAATGATCAACAACTCTTCTTGTACAT

CGGCTCAAATCCTGCTAACAACAACACTACATTTGTTGAAGACAACTCACTGCCGTCCTT

CTCAAGAGCTGTTAATCAGAGGGATGCTGATCTTGTCTACTTCTGGCACAAGTACCAGAA

ATTGGCTGAGAGCTCCACTGAGAAAAACGATGCTCGGAAGCAATTGCTCGAGATGATGAG

TCATAGATCTCATATTGACAACAGCGTCGAGCTGATTGGAAACCTTCTGTTTGGTTTTGC

GGATGGTCCAATGGTTCTAAAGACTGTTCGCCCAGCTGGCGAGCCTCTTGCTGATGACTG

GAGTTGTCTCAAGTCTATGGTGCGTGCTTTTGAATCACAATGTGGCTCGTTGGCGCAGTA

TGGAATGAAGCACATGCGGTCCTTTGCAAACATCTGCAATGCCGGCATCCTTCCTGAAGC

GATGGTGAAGATGGCTGCTCAGGCATGCACCAGCATCCCAACCAACCCCTGGA

>Contig465

TCTCTTGCGGAGGCCAACGGCAAGGCTTCCTCCCTTGCGCCCTCGATCAGTTCAGCGGTT

CGGCTCCTCGGGACCATTGTTGGTTCGCCGAAATGGAGTCACACAGTGATGACTTGCCAC

CACCACCACCACTCCCGCCAAATGCAGAGCCGATAAAAGCTGAGTCGGCTGATGACTTGC

CACCACCACCACCCCTGCCGCCTATCAAACCTGAAGAAGCAAAGAAGATCTCAAAGCCTA

AGAGGGCCCTGATCGCTCGTCCTGGTTTTGGCAAGAGGGGAAATCCTATACAGCTTGTGA

CAAATCATTTCAAAGTCTCGTTGAAGACGACAGATGAATTCTTCCATCATTACTATGTAA

ATCTGAAGTATGAAGATGACAGGCCTGTTGATGGAAAAGGTGTTGGTAGAAAAGTCATTG

ATAAGCTTGCTCAGACTTATCCATCGGAGCTAGCCCATAAAGACTTTGCCTATGATGGTG

AAAAGAGTCTTTTTACCATTGGTGCCCTCCCACAAATTAACAATGAGTTTGTTGTGGTTC

TTGAAGACGTTTCCAGTGGAAAGACTCCTGCAAATGGCAGCCCTGGAAACGACAGTCCAG

ACAAGAAGAGAGTGAAAAGGCCATATCAGACTAAAACCTTCAAGGTGGAGCTGAGTTTTG

CAGCTAGAATCCCCATGAGTGCTATTGCAATGGCACTCAAAGGCCAGGAATC

>Contig467

CGAGGCTCACCATTTACCAACCCCTCCGAGAGTCGGAAGCAGAGCCGCGATCGATGGCCG

GCACCGTGCCCAAGCTTCCCCTCGCAGCCCCTCCGCTCCGCCGCGCTCGCCTTCGCCCCC

TCCGCCCGCCGCTTCCGCGTCTCCCTCGCCGCCCGCGCCAGGAGCCCCGTCATTGCGATG

GCTTCCGCCAAGGAGGGGAACGGCGCCGTGACGAAGAGGACCACGCTCCATGACCTCTAT

GAGCAACAGGGTCAGTCCCCGTGGTACGACAACCTCTGCCGGCCTGTCACCGATTTGCTG

CCCTACATCGCCAACGGTGTCCGTGGAGTCACCAGCAACCCAACGATTTTCCAGAAAGCC

ATCTCTTCATCAAACGCGTATGACGGTCAGTTCAAGGAGCTTATATCGGCTGGGAAGGAT

GCAGAGAGCGCTTACTGGGAACTCGTCATAAAGGACATCCAAGACGCGTGCAAACTGTTT

GAGCCCATCTACGACGAGACCGATGGGGCTGATGGTTACGTTTCAGTGGAGGTGTCTCCT

AGGCTGGCAAATGACACCCAAGGAACCGTCGAAGCTGCGAAGTGGTTACACAAAGTGGTC

AACCGCCCCAATGTGTACATCAAGATCCCAGCTACTGCAGAATGCGTTCCTTCTATCAAG

GAAGTCATTGCTAATGGCATCAGCGTCAATGTCACTCTTATCTTCTCCGTTGCAAGATAC

GAGGCTGTGATTGATGCTTACCTTGACGGGCTTGAGGCTTCTGGTTTGAGTGACTTATCC

CGAGTGACCAGTGTAGCATCCTTCTTTGTCAGCCGAGTTGACAGTCTGATCGACAAAATG

CTTGAGAAGATTGGAACACCTGAGGCTCTTGCCCTGAGGGGAAAGGCTGCTGTAGCACAA

GCAAAGATAGCAAACCAGCTTTACCTCAAGAAATTCTCTGGCCCA

>Contig470

GTCAAGAAAACCTTGTCCCAGTTGCGGAATTGGTATATGGTGCAATGGGGTCTGCTGAGT

TGGAGAAAGCTGTCGAGAAAGAGTATGAAATGGCTCTTCAAGACAGAGTTATGGAAGAAA

CCAAAGAGAAGAAGAATTCTGTGGAGGCTTATGTTTATGACATGCGTAACAAGCTTTATG

AGAAGTACTCTGATTATGTTACATCGGAGGACAAAGAAACTTTGACGGCTAAGCTTCAGG

AGGTTGAGGATTGGCTGTACGAAGATGGCGAGGATGAGACCAAGGGAGTCTATGTTGCAA

AACTGGAAGAACTTAAAAAGGTTGGTGGCCCTATCGAGGCGCGCTACAGAGAGTGGGAAG

AAAGAGGTCCTGCTATTGAGCAACTAGCGTACTGCATCCGCAGTTTCAGGGAGGCTGCAT

TGTCTAGTGACCCAAAGTTTGAGCATATCGACATATCGGAGAAACAAAAGGTTGTTAATG

AGTGCTCGGGAGCGGAGACTTGGCTGCATGAGAAAAAACAGCAGCAGGATGCTTTACCAA

AGCATGCTGACCCTGCTCTCCTTGTTTCCGACATTAAGAAGAAGGCGGAAGCACTTGACA

GATTTTGCAAATCGATCATGACAAAGCCAAAGCCAGCACCGAAGCCACAGACCCCACCCC

CGGCCGAAACTTCACCACCAGAGGCTCAAACACCAGAGCAGCAGCCAGATGGTGCCACTG

AAGCTAGTGAGCCGGCCAGTGAGGGAGGTGCGTGGGAGCAGCCTGCAGCCGAGCAGATGG

ACACCGATGAACCCGATCCTTCCTCGGCATAGGCTTATTGTTGATCGAAGCAGTTTTTTT

TAATCTCTGCTATTGATTGCGTTTCACGGTGTTGGTAGCGCGGGAAGCTTTTGGGTCTTG

TGGCACGCACGGTTGCGAGCAAGGTTATGTATGATGTGTTGGAACTTTGAGAGAGTTCGG

TAGTGTATGTAGCTGTCTGTCCCTAGACTTGGTTTTCTGGCTTTCTTGTTGGTGGGTGAT

GCAGATGCAGGGTTATCATCTTC

>Contig473

TGATCCATTCAGAAATCATCACATGATATGCAGGTAAGGATGTGCGGATTGGTCATTTTG

ATTTTGTCAAAATATATTTATTTTTTTGTATCTTTTTACCCCTCTGGACATTATTGTGTG

CAGATACAGAAGCGAGCCCCATGTTCCATGGTCTGCTATTACTACCCCATCTGGTGTCTT

CGTTATACTTATGCTTATCGGCTACATAATATATGCTGCGTGGAATCGCTATGATAGCGT

TACGGAAGATTGCCGGAAAATGAAAGCACTGAAAAAACGGGCGGAAGCCGCTGATGTTGC

TAAGTCTCAGTTCCTTGCAACTGTTTCTCATGAGATCAGGACACCCATGAACGGTGTGCT

AGGAATGCTTGATATGCTATTAGACACAGAGCTGAAGTCAACCCAGAGAGATTTTGCGCA

AACTGCCCAAATCTGCGGAAAGGCATTGATATCCCTAATTAATGAAGTGCTTGACAGGGC

CAAAATTGAAGCTGGCAAGTTGGAGCTAGAGTCTGTACCGTTTGACCTTAGGTCCATCCT

TGATGAAGTCGTCTCTCTATTTTCTTCGAAATCAAGAGAAAAGGGAATTGAGCTTGCGGT

ATATGTCTCTGAAAGAGTTCCTGAAATCCTGCTGGGCGATCCTGGAAGGTTCCGTCAGAT

AATTACAAACTTAGTTGGGAACTCAATTAAGTTCACAGAAAGGGGACACATTTTTGTGCA

AGTTCACCTGGCAGACCATTCAAATCTAGCAACTGAACCAAAAGTTGAATCAGTCGCGAA

TGGCATCAATGGACATATAGATGAGACAAGTGTTGTATCCACAAGCGTGCCACACAATAC

ACTAAGTGGTTTTGAGGCTGCTGACAGCAGAAATAGCTGGGAAAACTTCAAGGCTTTGCT

TTCTTATGACACAAATGATATGGCGTATGGAAGTGATTCTGGGAATGTCACTCTTGTAGT

AAGTGTGGAAGATACAGGGATAGGTATACCAATTCATGCCCAAGGCCGGGTCTTCATGCC

TTTCATGCAAGCTGATAGTTCAACATCTAGAAACTATGGTGGGACTGGAATTGGATTGAG

TATCAGCAAATGTCTTGTTGAACTAATGAGTGGTCAGATAAACTTTGTCAGTCGACCCAA

TGTTGGGAGCACATTTACATTCACCGCAGTCCTGCAACGGTGTGAGAGAAATGCTATTAA

TGTCAGCAAGTCTGCTTTGTTGCATCCTCTGCCATCCAGTTTTCAAGGTCTATCCACACT

GCTGGTTGAT

>Contig474

CGACGTGCCGGGGCTGGGCCCCGATGCCTTCATCCCGCCGGAGGAGGTCCGGCGGAGCCG

CTTCTACGACGCCATGGCCGCCGGCGGCGGCAAGTGAACGTACTACTCGATCGTACGCAG

GAGTGTAACACGTAGTAGTCCAGTAGCATTTTGTTTAGTTTCGTCGGTGTGTCGATTATT

GTTTGTGATTGTGGCACCAAGAAAATGCAAGCGCGTGTACATACATATGTGTACCCTGGG

TTTTTACCTAAAAAGAAAGAAAAACTCACAGTTTCTAAAAAAAAAAAAAAGACGATGGAG

TACCAGGGGCAGACCGGCCACGCCACCGACAAGGTGGAGGAGTACGGCCAGCCCGTGGCC

GACCAAGCTCCAGCCGACCAGGGACGACCACAAGACCGACGGCGTCCTGCGCCGCTCCGG

CAGCTCCAGCTCCAGCTCGTCTGAGGACGACGGCGCTGGCGGGAGGAGGAAGAAAGGGAT

GAA
